# Increased functional unit flexibility and solvent accessibility favours oxygen capture in molluscan hemocyanin†

**DOI:** 10.1039/d5ra03248b

**Published:** 2025-06-16

**Authors:** James G. Davies, James A. Platts, Mark T. Young, Georgina E. Menzies

**Affiliations:** a School of Biosciences, Cardiff University Museum Avenue Cardiff CF10 3AX UK MenziesG@cardiff.ac.uk YoungMT@cardiff.ac.uk; b School of Chemistry, Cardiff University Park Place Cardiff CF10 3AX UK

## Abstract

Hemocyanins are a class of copper-based oxygen transport proteins, widely studied for their unique oxygen-binding processes and their role in the molluscan immune response. In this study, we utilised computational simulations to investigate the first functional unit (FU-a) of *Crepidula fornicata* (slipper limpet) hemocyanin, a member of the keyhole limpet hemocyanin family. Using quantum mechanics/molecular mechanics (QM/MM) methods, we designed oxygenated and deoxygenated models of FU-a and conducted molecular dynamics simulations to explore their functional dynamics and oxygen accessibility. We specifically focused on understanding the global and localised dynamics between the two conformational states. By employing principal component analysis (PCA) and modevector analysis, we differentiated the dynamic properties of the deoxygenated and oxygenated states of the hemocyanin. Furthermore, we explored the impact of oxygenation on hydration and tunnel cavity formation. Our results reveal that oxygen entry is mediated by a single bidirectional tunnel, with its permeability tightly regulated by differential histidine-based copper coordination. Importantly, we identified Glu352 as an evolutionary conserved molecular “shutter,” whose conformational changes govern the opening and closure of this tunnel. These findings provide insight into the mechanistic regulation of oxygen transport in molluscan hemocyanins, with implications for understanding their functional versatility and potential applications.

## Abstract 2

Model construction, parametrisation of binuclear copper sites, MD simulation and trajectory analysis of oxygenated and deoxygenated slipper limpet hemocyanin FUa.

## Graphical abstract

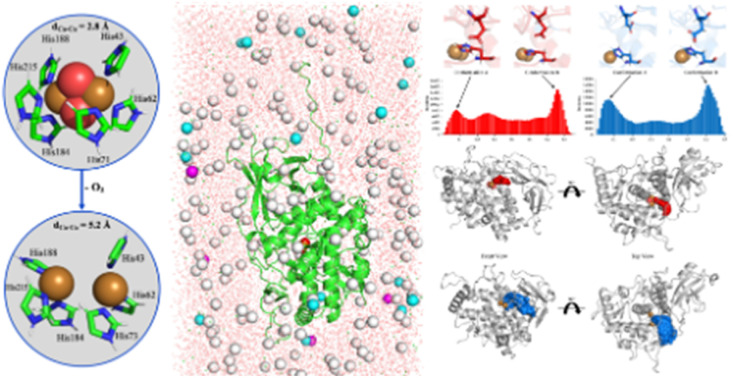

## Introduction

Hemocyanins are large copper-containing multimeric oxygen transport proteins found in arthropods and molluscs.^1^ In addition to their role in oxygen transport, hemocyanins have been implicated in the molluscan immune response, both as phenoloxidase enzymes, where proteolysis or partial denaturation enables tyrosine and derivatives to access the copper–oxygen centre,^2,3^ and as a source of antimicrobial peptides.^4,5^ These functional roles underline the importance of understanding the 3D-structure and dynamics of molluscan hemocyanins, including their subunit arrangement and interfaces, structural transitions and oxygen binding characteristics.

While hemocyanins from arthropods are hexamers,^6^ molluscan hemocyanins are typically either decamers or didecamers comprised of 10–20 subunits.^1^ The most widely studied of the molluscan hemocyanins, the keyhole limpet class, are typically didecamers of 20 subunits arranged with d5 symmetry, where each decamer is made up of 5 dimer ‘plates’ arranged in 5-fold rotational symmetry.^7^ Each dimer plate contains 2 identical subunits but is effectively a heterodimer due to the differential structural arrangement of functional units (FUs) within the two monomers.^8^ Each monomer, approximately 3500 amino acids in length, is composed of 8 FUs (FU-a–FU-h), which contain an alpha-helical core domain containing a Type 3 copper–oxygen centre, and an adjacent beta-structure thought to regulate access to the copper–oxygen centre.^9^ A molluscan hemocyanin didecamer therefore contains 160 oxygen binding sites, which also show cooperativity,^2^ likely arising from interactions between FUs from the same or different subunits,^10,11^ although the precise molecular mechanisms remain to be elucidated.

Oxygen binding to hemocyanins has been extensively studied, and the copper–oxygen centres of each FU are highly conserved across arthropods and molluscs, with 2 copper ions coordinated to 6 histidine residues.^7^ In the deoxygenated form, the Cu ions are in the Cu(i) oxidation state, whereas in the oxygenated form (Cu_2_O_2_), they are oxidised to the Cu(ii) state. Crystallographic studies of horseshoe crab (*Limulus polyphemus*) hemocyanin have demonstrated that upon oxygen binding, the Cu–Cu distance shortens by approximately 1 Å.^12,13^ The oxygen binding structural transition in horseshoe crab hemocyanin has also been studied using molecular dynamics (MD) simulations, investigating the effect of hydration on facilitation of oxygen access to the copper centres,^14^ and how inter-subunit dynamics controls the formation of tunnels for oxygen in the hemocyanin structure.^15^ Most recently, a quantum mechanics/molecular mechanics approach (QM/MM) has been employed to probe the oxygenation process in detail, showing the formation of a stable superoxide intermediate consistent with previous experimental data.^16^

In this work we set out to analyse the molecular dynamics of oxygen binding in a molluscan hemocyanin of the keyhole limpet class, slipper limpet (*Crepidula fornicata*) hemocyanin (SLH), building on our recent work determining its structure using a combination of cryoEM and Alphafold2.^17^ We modelled the 3D-structure of FU-a, generated custom parameters for the Cu_2_ and Cu_2_–O_2_ deoxygenated and oxygenated states, and show that oxygen entry is mediated by a single bidirectional tunnel, with its permeability tightly regulated by differential histidine-based copper coordination. We identify Glu352 as a conserved molecular “shutter,” whose conformational changes govern the opening and closure of this tunnel. We also demonstrate that the deoxygenated state displays increased flexibility and solvent accessibility, proposing that this favours oxygen capture.

## Methods

### Starting structures

Slipper limpet hemocyanin naturally forms large multimeric assemblies, typically decameric and didecameric in size.^17^ The sequence for FU-a was extracted from the SLH1 protein sequence (GenBank accession number WCA44164.1), its signal sequence was removed and the 3D structure (deoxygenated and devoid of copper ions) modelled using Alphafold3.^17,18^ To accurately model the copper–oxygen centre, AlphaFill was employed to incorporate Cu_2_ clusters into both the deoxygenated (FU-a_deoxy_) and oxygenated (FU-a_oxy_) states, with the latter additionally incorporating oxygen.^19^ The resulting coordination geometries were carefully analysed alongside our previously developed, unpublished AlphaFold2-based model of SLH, yielding a root-mean-square deviation (RMSD) of <1 Å. Following this, the structures were further refined using the following QM/MM methods to achieve optimal electronic and structural fidelity.

### Computational methods

Molecular dynamics (MD) simulations were conducted using the GROMACS software package.^20^ Due to the absence of force field parameters for the protein's coordination of the two metal sites, custom parameters were generated using the MCPB.py module of the AMBER suite.^21^ Bond angles, bond lengths, and partial charges were calculated through Gaussian09 software, with B3LYP/Def2-TZVP.^22,23^ Dicopper clusters were assigned overall charges of +1 and +2, respectively, corresponding to the FU-a_deoxy_ and FU-a_oxy_ states, both with singlet multiplicities. Harmonic force constants and atomic charges were derived from DFT calculations using the Seminario method and restrained electrostatic potential (RESP) fitting scheme.^24–27^ The resulting parameters were then integrated with the AMBER's 2019 Stony Brook forcefield (ff19SB) using the LEaP module.^28^ Protonation states of titratable residues were determined at pH 8.07 and a salt concentration of 0.6 mM, simulating seawater conditions typical of the North Atlantic Ocean.^29,30^ The systems were then solvated using the explicit water model TIP3P.^31^ Temperature coupling was applied using the v-rescale thermostat, while Particle-Mesh Ewald (PME) was employed for long-range electrostatics. Simulations were performed under the NPT ensemble with periodic boundary conditions (PBC), at a temperature of 285.43 K and a pressure of 1 atm.^32^ After energy minimisation and equilibration, five independent molecular dynamics simulations were executed for each system, consisting of 500 million steps with a 2 fs timestep, yielding a total simulation time of 5 microseconds per system.

### Trajectory analysis

Root-mean-square deviation (RMSD), root-mean-square fluctuations (RMSF), and radius of gyration (*R*_g_) were monitored to assess protein stability. Principal component analysis (PCA) was employed to extract dominant conformational motions and delineate structural differences between states. The covariance matrix of atomic fluctuations was constructed and diagonalised, yielding eigenvalues and corresponding eigenvectors, with eigenvalues normalised and ranked to prioritise principal motions. Eigenvectors were subsequently extracted, normalised, and sorted by their significance. A dot product matrix was computed for the leading principal components, quantifying subspace overlap and characterising shared conformational dynamics between states. Complementary metrics, including subspace overlap and maximal dot products, provided a comprehensive measure of similarity in dominant motions. Gaussian kernel density estimation was applied to principal component projections, generating contour maps that illustrate the distribution of sampled conformations, offering both quantitative and visual insights into the structural landscapes explored throughout the simulations.

### Binding free energy calculation

Binding free energy was computed using gmx_MMPBSA, selected for its compatibility with GROMACS trajectories and its capacity to evaluate both Poisson–Boltzmann Surface Area (PBSA) and Generalized-Born Surface Area (GBSA) methods.^33^ Calculations were performed on the equilibrated section of the MD simulation (100–1000 ns), focusing on the energetics between coordinating histidines and the active-site cargo. Given the significant electrostatic polarisation within the metal center, the internal dielectric constant was varied between 1.0 and 5.0 to assess its impact on the free energy calculations. For GBSA, a GB-Neck2 model was employed to accurately capture complex solvation environment presented in this study.^34^ PBSA calculations followed similar parameters; however, given that the GB-Neck2 model was too computationally expensive with the available resources, the GB-OBC1 model was used instead.^35^ In all cases, the Δ*G*_binding_ was reported as relative binding free energy, with the contribution of conformational entropy excluded from the evaluation.

### Tunnel formation analysis

Dynamic tunnel formation was analysed using CAVER 3.0.^36^ Every tenth frame from the equilibrated segment (100–1000 ns) of each replicate was extracted, resulting in a total of 90 000 frames for analysis encompassing both FU-a_deoxy_ and FU-a_oxy_. These frames were processed following the CAVER protocol, with copper ions designated as reference points for tunnel identification. To ensure the identification of only sufficiently sized tunnels, a probe radius of 1.5 Å, corresponding to the van der Waals radius of transported oxygen, was applied. A clustering threshold of 3.0 Å was employed to refine the accuracy of tunnel detection. The bottleneck radius was then calculated to determine the maximum probe size that could pass through the narrowest section of each tunnel. Tunnels were further assessed based on throughput, ranging from 0 to 1, where a higher value indicated greater functional relevance of the channel.^36^

### Residue interaction network analysis

Residue interaction networks (RIN) were constructed to examine the alterations in local interaction networks upon oxygen binding. Analysis focused on the equilibrated segment of each replicate (100–1000 ns), with every tenth frame extracted in PDB format. The Residue Interaction Network Generator 4.0 (RING 4.0) was utilised to quantify contact frequencies across the entire protein, incorporating predefined physicochemical properties and geometric constraints in its calculation framework.^37^ These probabilities were subsequently averaged across replicates and compared between states. RIN data was processed using in-house Python scripts, leveraging NetworkX and Matplotlib to visualise the interaction networks, where amino acids were represented as nodes, and their interactions as edges.^38,39^ A key step in the analysis was identifying signal-propagating nodes within a maximum distance of 2 steps from the highlighted residue, Glu352, using a threshold of 0.3 for interaction strength. Contacts were classified as exclusive to FU-a_oxy_, FU-a_deoxy_, or shared between both states.

## Results and discussion

Oxygen capture in molluscan hemocyanins is a highly conserved process driven by copper–oxygen interactions across multiple copper-binding sites. In deoxygenated hemocyanin, each copper ion is trigonally coordinated by three histidyl imidazole nitrogens.^12,13,40–42^ Upon oxygenation, oxygen binds as a peroxide, forming a μ:η^2^-η^2^ bridge between the two copper atoms.^43^ This binding induces a shift in oxidation state from Cu(i) to Cu(ii), stabilising the peroxo-like configuration.

In addition to the peroxo form, oxygen can also bind in the bis-μ-oxo form, which is crucial for studying the flexibility and reactivity of copper–oxygen sites in hemocyanins.^44–46^ In this form, the Cu–Cu distance reduces to approximately 2.8 Å, compared to 3.6 Å in the peroxo form, enhancing the interaction between copper and oxygen.^47^ The isomerisation between the peroxo and bis-μ-oxo forms plays a critical role in oxygen activation and O–O bond cleavage, key processes for both oxygen binding and reactive oxygen species (ROS) deactivation in hemocyanins. Theoretical models indicate that this bis-μ-oxo form is energetically stable, with interactions between Cu 3d and O_2_ π* orbitals contributing to its stability. These models suggest the bis-μ-oxo form offers a favourable pathway for oxygen species activation.^48–51^ Given its stability and the pronounced difference in Cu–Cu distance, we selected the bis-μ-oxo form for our MD simulations. This choice enhances the distinction between the oxygenated and deoxygenated states, while remaining biologically relevant to hemocyanin function.

Previous studies have explored the parametrisation and subsequent simulation of horseshoe crab (arthropod) hemocyanin structures with resolved copper–oxygen sites.^14,15^ We have extended this work by modelling copper–oxygen sites into slipper limpet (molluscan) hemocyanin, using a model where these sites have not yet been resolved structurally. We first aimed to optimise the orientation of the copper–oxygen site in our model with high accuracy, permitting MD simulations.

### Parameterisation of metal sites

**Fig. 1:**
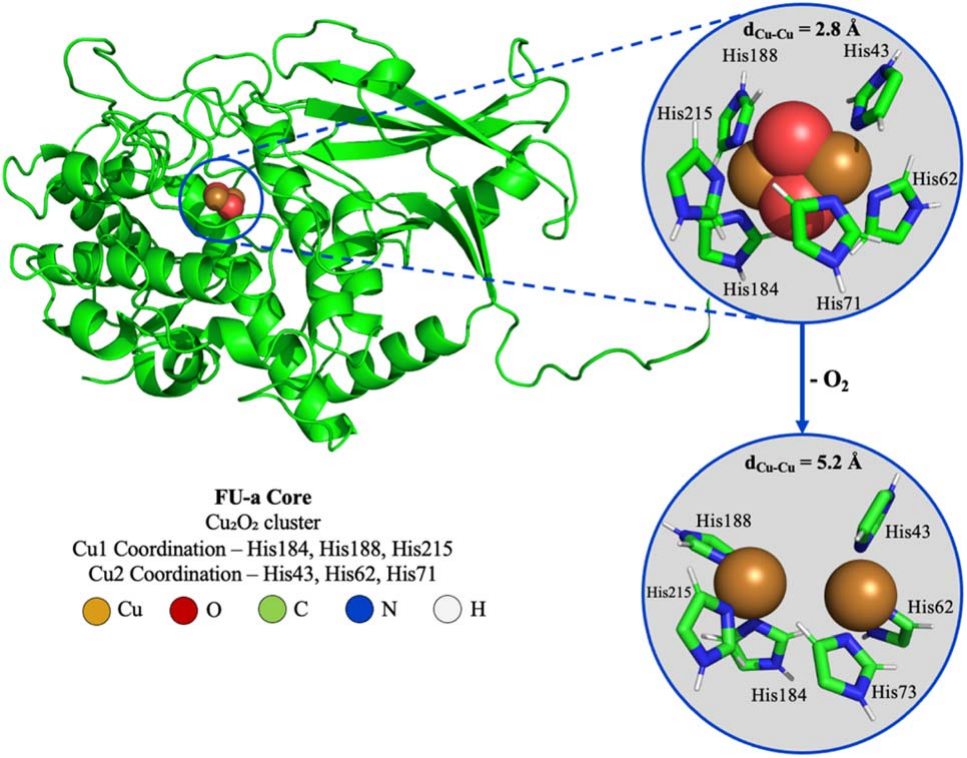
Structural representation of the FU-a core highlighting the Cu_2_O_2_ core and imidazole groups from the coordinating histidines. Circled areas represent minimum energy structures obtained at the B3LYP/Def2-TZVP level of theory of the oxygenated (FU-a_oxy_; top) and deoxygenated (FU-a_deoxy_; bottom) core. Atom colour codes: Cu (orange), O (red), C (green), N (blue), H (white).

To facilitate the simulation of SLH FU-a, we first parameterised two distinct FU-a models: FU-a_oxy_, containing a Cu_2_O_2_ core, and FU-a_deoxy_, featuring a fully deoxygenated Cu_2_ core (Fig. 1), using AlphaFill to incorporate the Cu_2_/Cu_2_O_2_ site.^19,52^ The resulting structures were then parameterised using the MCPB tool and subjected to geometric optimisations at the B3LYP functional level with the Def2-TZVP basis set.^21,23^ Geometrical parameters, including bond lengths and angles for the FU-a active sites, are presented in Tables S1 and S2.† QM calculations reveal that in FU-a_deoxy_, copper coordination forces are distributed relatively uniformly among the coordinating histidines, with minimal deviations in equilibrium bond lengths and force constants across both histidine triplets. In contrast, for FU-a_oxy_, a distinct coordination pattern emerges: two histidine ligands exhibit strengthened interactions with the copper centre, while the third shows weakened association. This asymmetric coordination is replicated at both coordination sites, and results in a reduced interaction involving His71 and His215. This suggests a potential local destabilisation or altered electronic environment at these positions, which could affect overall structural stability, or influence reactivity in the Cu_2_ core.

The stability of both the coordination models was then evaluated by computing the root-mean-square deviation (RMSD) of activate site heavy atoms over time. Mean RMSD values, averaged across the equilibrated region of each MD replicate, indicate greater stability in FU-a_oxy_ (0.52Å ± 0.11) relative to FU-a_deoxy_ (1.00Å ± 0.19). This suggests that oxygen binding enhances rigidity within the active site, allowing ligating residues to adopt a more stable conformation more rapidly than in the deoxygenated state.

Additionally, the minor increase in RMSD variability observed in FU-a_deoxy_ replicates is consistent with the increased unoccupied volume within the active dicopper centre, permitting greater conformational flexibility among ligating residues. To establish which ligating residues contributed most to this difference in coordination stability, equilibrium bond lengths obtained from QM-optimised active sites were compared to those derived from MD trajectories. We observe a mean bond length increase of 1.9% in FU-a_oxy_ and 12.6% in FU-a_deoxy_, with His73 and His62 exhibiting the most substantial elongation for each state respectively (Table S3†). While these changes deviate from the ideal bond lengths predicted by DFT calculations, the standard deviation of the copper-ligand distances remains below 0.15 Å in all cases. This suggests that, despite the slight elongation, the bonds retain a high degree of stability, preserving the active coordination environment of the copper centers.

Moreover, when comparing the inter-copper distance (*d*_Cu–Cu_), a distinct change occurs upon oxygen binding, reducing *d*_Cu–Cu_ from 5.07 Å in FU-a_deoxy_ to 2.84 Å in FU-a_oxy_ – consistent with the copper association observed in other dicopper–oxygen complexes.^13,53–55^ Overall, these results support the stabilising influence of oxygen by way of elevated rigidity within the active site, a concept previously reported in *Limulus polyphemus*.^14^ The observed bond distances accurately reflect the conserved transformation undertaken by the dicopper centre upon oxygenation. While slight elongation of ligating distances is evident, their stability remain intact. This elongation likely reflects the influence of dynamic simulation conditions, which capture temperature, pressure, and solvent effects not represented in prior theoretical studies.

### Active site energetics

Building on the structural observations discussed previously, we next evaluated the energetics of active site coordination in both oxygenated and deoxygenated states of FU-a. Specifically, we quantified the binding free energy (Δ*G*_bind_) between the coordinating histidines and the active site cargo: Cu_2_ (in the case of FU-a_deoxy_) and Cu_2_O_2_ (for FU-a_oxy_) using both MM/GBSA and MM/PBSA end-state methods (Table 1). Accurate implementation of the gmx_MMPBSA tool requires careful parameter definition, particularly the internal dielectric constant (IDC), to reliably assess binding free energies. To enhance the robustness of our approach, as such, we broadened our IDC parameter range, drawing on insights from a previous study that assessed the influence of parameter selection on calculation accuracy.^56^

**Table 1:** Binding free energies from the MM/GBSA and MM/PBSA calculations for FU-a_oxy_ and FU-a_deoxy_ systems

Δ*G*_bind_ (kcal mol^−1^)	FU-a_oxy_	SD	FU-a_deoxy_	SD
MM/GBSA	−17.72	1.35	−1.29	3.04
MM/PBSA	−19.85	1.42	−4.78	3.01

In FU-a_deoxy_ systems, Δ*G*_bind_ values were largely insensitive to variations in the internal dielectric constant (IDC) (Tables S4 and S5†). In contrast, FU-a_oxy_ systems exhibited pronounced sensitivity, with Δ*G*_bind_ values ranging from −72.06 to −17.50 kcal mol^−1^ using MM/GBSA and from −80.31 to −19.85 kcal mol^−1^ using MM/PBSA. Although increasing the IDC resulted in progressively less favourable binding energies, it concurrently reduced variability across replicates. This trend suggests that incorporating greater electronic polarisation improves the stability and convergence of free energy estimates. Although the dependence of binding energy on dielectric constant used indicates that absolute binding energy from MM/PBSA approach may not be reliable, as discussed by Roux and Chipot, comparison of oxygenated with deoxygenated form should allow us to qualitatively determine relative binding energy.^57^ Consequently, an IDC value of *ε* = 5 was selected for both states to ensure consistency and methodological robustness.

Comparison of the final free energy values highlights the notable influence of oxygenation on active site energetics. MM/GBSA calculations revealed a Δ*G*_bind_ difference of 16.43 kcal mol^−1^ between FU-a_deoxy_ and FU-a_oxy_, with the latter displaying more favourable binding (Table 1). Similarly, MM/PBSA calculations indicated a 15.07 kcal mol^−1^ difference, reinforcing the enhanced thermodynamic stability of the oxygenated coordination environment. These findings are consistent with the proposed compaction and increased rigidity of the active site upon oxygen binding and underscore the intrinsic energetic favourability of oxygen coordination – an expected feature for proteins of this nature.

This energetic preference is consistent with prior studies of type-3 copper containing metalloproteins. Saito *et al.* reported a binding energy of −18.9 kcal mol^−1^ for oxygenation in the T-state of *Limulus polyphemus* hemocyanin, a value that overestimates the experimental binding enthalpy range of −11.5 to −6.0 kcal mol^−1^.^58–60^ In contrast, a more modest binding energy of −2.3 kcal mol^−1^ was reported by Fasulo *et al.*, who used the NEVPT2 method to obtain a result which better aligns with experimental observation.^16^ The 15.07 kcal mol^−1^ difference observed in this study falls within the expected range of hemocyanin-like systems, particularly given the use of classical MD-based free energy methods, in contrast to the QM or QM/MM approaches used in previous work, alongside expected differences in protein environment. Nonetheless, the consistent stabilisation observed upon oxygen binding in FU-a_oxy_ strengthens our confidence in the present parametrisation, and mirrors trends reported across both theoretical and biological systems.

### Global dynamics

**Fig. 2:**
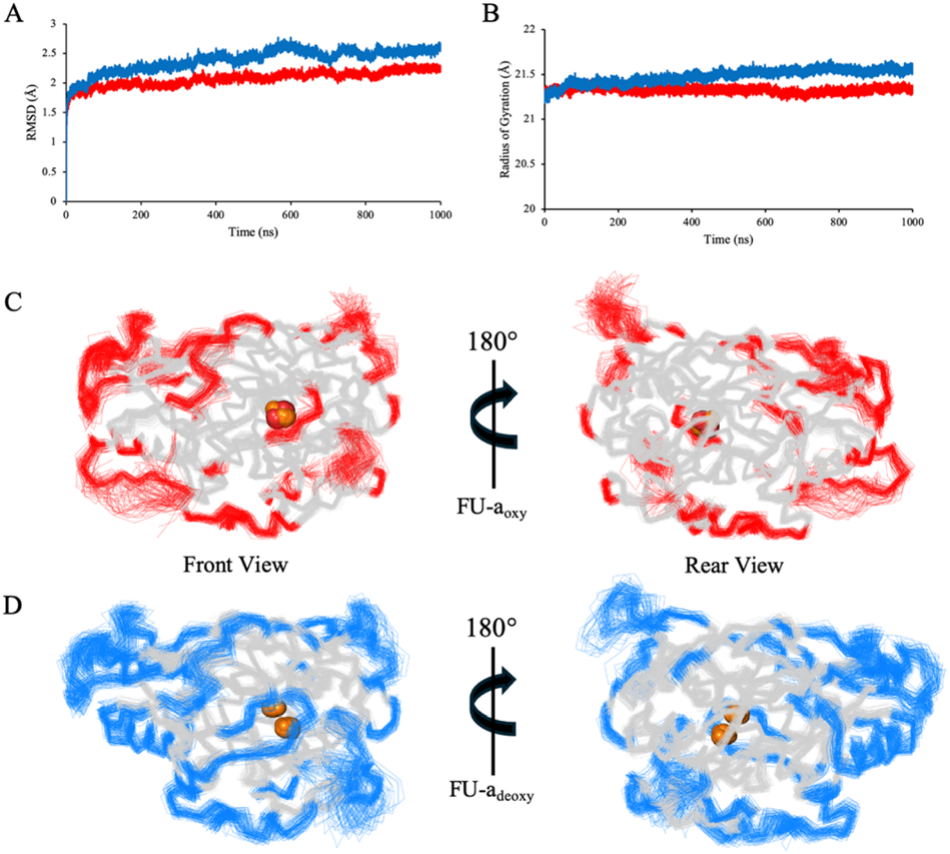
A comparison of global structural stability and residue-level flexibility between FU-a_oxy_ and FU-a_deoxy_ replicates. (A) Average Ca RMSD values for FU-a_oxy_ (red) and FU-a_deoxy_ (blue) calculated over the equilibrated portion of the trajectories. (B) Average *R*_g_ for FU-a_oxy_ (red) and FU-a_deoxy_, indicating a modest increase in structural compaction upon oxygenation. (C) Front and rear views of FU-a_oxy_, with residues exhibiting RMSF values of ≥ 1 Å highlighted in red. (D) Corresponding views of FU-a_deoxy_, with highly mobile residues highlighted in blue.

To gain a deeper insight into the functional consequence of oxygenation, we extended our analysis beyond the active site to examine the broader dynamical features of FU-a. To ensure an accurate representation of core dynamics, only residues 1–407 were included, thereby excluding the highly flexible C-terminal tail which would otherwise link adjacent functional units in higher order hemocyanin assemblies. RMSD and radius of gyration (*R*_g_) were used as real-time indicators of global conformational stability and compactness (Fig. 2A and B). Mean RMSD values calculated over the equilibrated portion of the trajectories were 2.10 Å ± 0.18 for FU-a_oxy_ and 2.46 Å ± 0.21 for FU-a_deoxy_, indicating a modest increase in global stability upon oxygen binding. This trend was reaffirmed by *R*_g_ analysis, which revealed a slight increase in compaction of FU-a_oxy_ (21.33 ± 0.07 Å) relative to FU-a_deoxy_ (21.51 ± 0.11 Å). Collectively, these observations suggest that oxygenation enhances structural compaction and promotes a more ordered global protein architecture, a phenomenon that has also been reported in previous studies of higher order hemocyanin assemblies.^14,15,61^

Root-mean-square fluctuation (RMSF) was employed to assess residue-level flexibility across the equilibrated regions of the simulation and revealed broadly similar patterns between FU-a_oxy_ and FU-a_deoxy_ (Fig. 2C and D). To better resolve subtle localised differences, per-residue values for FU-a_oxy_ were subtracted from those of FU-a_deoxy_. Residues exhibiting absolute differences exceeding ± 0.5 Å were subject to further analysis (Fig. S1†). As anticipated, the largest discrepancies in flexibility were predominantly localised to loop regions, specifically residues Pro46–Ala56, His133–His137, Ser153–Pro154, Ala350, and His374–Phe378. Among these, the loop spanning Pro46–Ala56 was particularly noteworthy, given its apparent interactions with neighbouring lops near the active site. The increased mobility observed in the deoxygenated state suggests a loss of inter-loop connectivity, potentially destabilising the local architecture. Interestingly, His43 and His62 – key residues involved in copper coordination – are positioned near regions of elevated flexibility and are closely spaced in the primary sequence. This suggests a potential link between the local structural dynamics and the coordination of the dicopper center. Together with the observed dynamic shifts in these loops and their proximity to the active site, these findings support a possible role for these regions in facilitating oxygen entry from the solvent environment.

### Principal component analysis

While the analysis above provides valuable insight into structural stability and local fluctuation, they do not capture the collective, correlated motions that often underpin functional dynamics. To explore these broader conformational patterns and directly compare dominant motion profiles between the oxygenated and deoxygenated states, we performed principal component analysis (PCA). This dimensionality reduction technique allowed us to identify the essential dynamical subspaces by isolating the most pronounced motions sampled during the simulation.

**Fig. 3:**
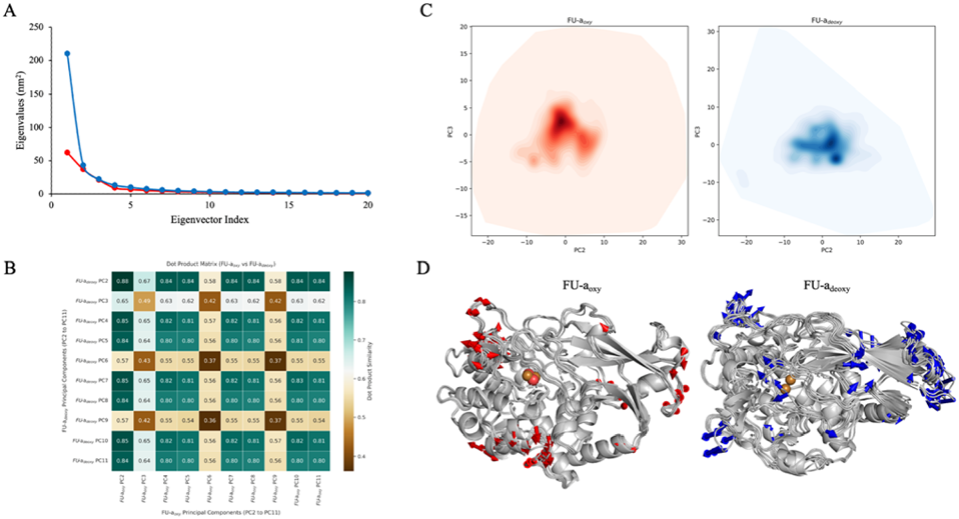
Principal component analysis (PCA) of the collective motions in FU-a_oxy_ and FU-a_deoxy_. (A) Scree plot showing the distribution of variance explained by the top 20 PCs for both oxygenated and deoxygenated states. (B) Subspace comparison based on dot product analysis of eigenvectors. (C) Projection of conformational sampling along PC2 and PC3 visualised as density heatmaps. (D) Structural visualisation of the dominant collective motions represented by PC2 in both FU-a_oxy_ and FU-a_deoxy_. Modevectors represented as arrows depict the direction and magnitude of atomic displacement.

Initial assessment of the variance distribution *via* scree plot (Fig. 3A) revealed that the first principal component (PC1) accounted for a disproportionately large portion of the variance in both FU-a_oxy_ and FU-a_deoxy_. This finding suggested that PC1 primarily reflected motions of the highly flexible C-terminal tail, a region excluded from previous analysis due to its intrinsic disorder. As such, PC1 was excluded from subsequent evaluation to prevent it from dominating the interpretation of the collective dynamics. This adjustment increased the number of components required to explain 95% of the total variance – from 13 to 28 in FU-a_oxy_, and from 5 to 24 in FU-a_deoxy_ – offering a more accurate view of the dynamics involved in the oxygenation process.

To further investigate the dynamic similarity between the oxygenated and deoxygenated states, a pairwise comparison was performed using the dot product matrix of eigenvectors derived from PCA. This analysis quantitively assessed the overlap in dynamical subspace between FU-a_oxy_ and FU-a_deoxy_, revealing a subspace overlap of approximately 47.8%. This result aligns closely with the 45.7% overlap reported in Bux *et al.* (2021), indicating a degree of dynamic similarity between the two systems. However, some divergence from the reported value is expected due to species-specific variations in hemocyanin, as well as the order of assembly analysed.^15^

To directly compare the nature of the collective motions between the oxygenated and deoxygenated states, we calculated the dot product matrix of eigenvectors derived from the PCA. The analysis revealed an average maximum dot product if 0.774, indicating a substantial degree of localised similarity across several dominant PCs, suggesting notable overlap in the collective motions between the two states. The highest similarity was observed between FU-a_deoxy__PC2 and FU-a_oxy__PC2, with a dot product of 0.878, highlighting a particularly well-conserved mode of motion between the oxygenated and deoxygenated states (Fig. 3B and C).

Despite the overall similarity, subtle yet meaningful divergence was observed between the two PCs identified above to be most similar. To explore this further, we extracted the extreme conformations associated with FU-a_deoxy__PC2 and FU-a_oxy__PC2 and visualised their motions using directional modevector analysis. This method enables the simultaneous assessment of both the direction and magnitude of atomic displacement from average structures obtained from MD replicates. While most structural changes occurring in FU-a_oxy_ appeared conserved, though more pronounced, in the deoxygenated state, a notable exception occurred in which a loop region adjacent to the active site exhibited a high degree of displacement, a feature that was distinctly absent in FU-a_oxy_ replicates (Fig. 3D).

This specific loop had previously been highlighted in RMSF analysis, where it demonstrated a heightened flexibility in the deoxygenated state, an effect attributed to disruption coupling with an adjacent loop (Fig. S1†). Taken together, the findings from PCA and modevector analysis thus reinforce earlier observations, suggesting that oxygen binding plays a key role in global stabilisation, with particular impact on the loop spanning residues Pro46–Ala56 where the most pronounced conformational changes were observed.

### Active site accessibility

**Fig. 4:**
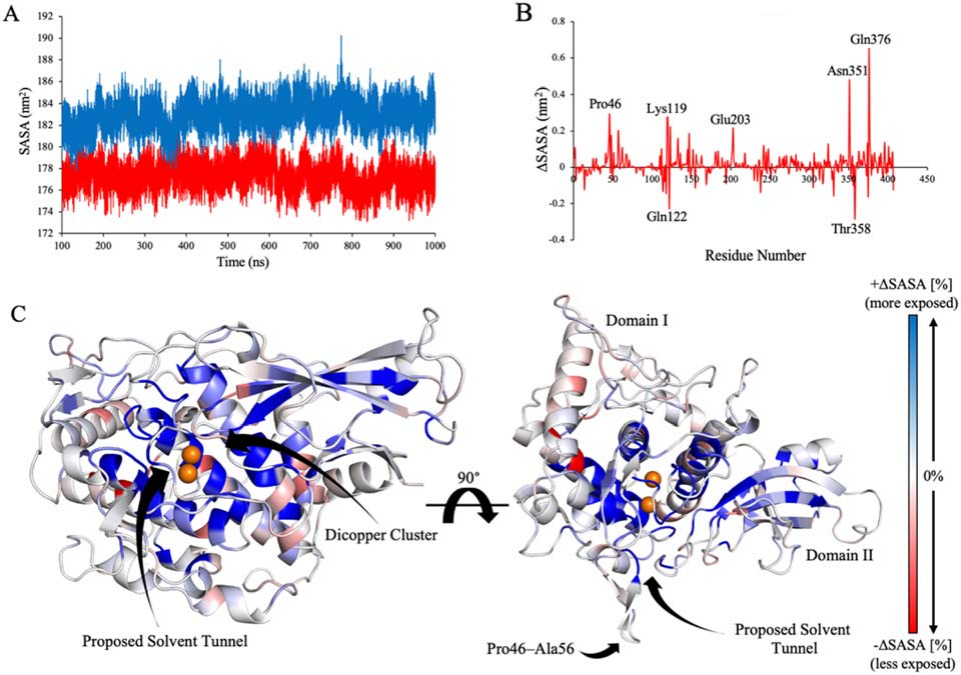
Solvent accessibility analysis of FU-a_oxy_ and FU-a_deoxy_. (A) Temporal profile of total solvent-accessible surface area (SASA) for FU-a_oxy_ (red) and FU-a_deoxy_ (blue). (B) Per-residue differential SASA calculated as SASA_deoxy_ – SASA_oxy_. (C) Structural mapping of per-residue solvent accessibility changes, with the proposed solvent cavity explicitly labelled. Residues are coloured on a gradient scale from blue (highest solvent accessibility) to red (lowest), illustrating the spatial distribution of exposure across the structure.

Building on the observation that oxygen binding induces distinct collective motions, specifically within loop regions proximal to the active site, we next assessed whether these changes in conformational dynamics were accompanied by changes in solvent accessibility. Solvent accessible surface area (SASA) was computed across each of the FU-a_oxy_ and FU-a_deoxy_ ensembles to quantify differences in solvent exposure attributable to oxygenation state (Fig. 4A). Globally, the FU-a_deoxy_ exhibited a higher SASA (1829.96 Å^2^ ± 42.92) relative to FU-a_oxy_ (1772.91 Å^2^ ± 28.12). Although the absolute difference was modest, the temporal consistency of SASA values between groups suggests a more compact and solvent-shielded architecture in FU-a_oxy_, consistent with the enhanced structural stability observed in previous analyses.

To elucidate the localised effects of oxygenation on solvent accessibility, residue-specific SASA differences were calculated between the FU-a_oxy_ and FU-a_deoxy_ ensembles (Fig. 4B). Notably, Gln122 and Thr358 exhibited notable reductions in solvent exposure upon oxygen binding (ΔSASA ≤ −0.2 nm^2^), while Pro46, Lys119, Glu203, Asn351, and Gln376 demonstrated marked increases (ΔSASA ≥ +0.2 nm^2^). Structural mapping of these differences revealed a spatially coherent pattern, with pronounced increases in solvent accessibility concentrated around the active site (Fig. 4C). Among these, Pro46, located on a loop previously identified as exhibiting the greatest conformational divergence between FU-a_oxy_ and FU-a_deoxy_, showed a substantial increase in exposure. Despite this, neighbouring residues within the same loop remained largely unaffected, indicating that the loop may not serve as a general gate for oxygen entry. Instead, these findings suggest that discrete residues within the active site microenvironment may play a more direct role in facilitating oxygen access.

These findings are consistent with prior theoretical investigations that identified solvent-accessible cavities at subunit interfaces within hemocyanin hexamers.^12–15^ Notably, Bux *et al.* (2021) reported a pronounced increase in solvent accessibility in the *Limulus polyphemus* hexamer upon deoxygenation, attributing this change to the collapse of solvent tunnels following oxygen binding.^15^ In parallel, our data reveal localised increases in solvent exposure near the active site, reinforcing the notion that dynamic modulation of solvent-accessible tunnels is a key component of the oxygenation mechanism. The convergence of these observations underscores the importance of conformational flexibility and transient tunnel formation in regulating oxygen transport within hemocyanin.

### Domain rotation

Together, the PCA and modevector analyses revealed both conserved and state-specific collective motions, underscoring the critical role of flexibility in the oxygenation mechanism. To further explore functional transitions at the quaternary level, we next focussed on domain rotation, a hallmark conformational response in hemocyanins linked to oxygen-mediated allosteric signalling. Structural comparison of *Limulus polyphemus* and *Panulirus interruptus* identifies a 7.5° rotation of domain 1 relative to domains 2 and 3, suggesting a concerted shift in the quaternary assembly upon oxygen binding.^12^ Similarly, analysis of *Limulus polyphemus* subunit II reveals a 3.2° rotation between the two trimers within the hexamer, reflecting a rotation at the subunit interface rather than at the domain level.^13^ This rotation is thought to be central to the regulation of cooperativity in higher order assemblies, where the movement of domain 1 induces an allosteric signal which propagates throughout the entire structure, thereby enhancing oxygen affinity cross all functional units. While this transition is well-characterised in arthropod hexamers, species-specific variation may suggest that the mechanism is not universally conserved. In our model, each functional unit consists of two domains – domain I (residues 1–305), predominantly helical, and domain II (residues 306–423), characterised by its β-sheet-rich architecture – rather than the previously described 3 domain structure. These structural differences raise the question of how the allosteric mechanism may vary across species. To address this, we quantified domain rotation in our FU-a models by aligning every 10th frame of domain I to a single FU-a_deoxy_ reference structure. Centre-of-mass calculations were performed to determine the relative movement of domain II with respect to the fixed domain I (Fig. S2†). This analysis revealed a 1.46° inward rotation of domain II towards the active site upon oxygen binding, a motion that was deemed to be highly statistically significant (*P* < 0.0001) (Fig. S3A†).

Intriguingly, Magnus *et al.* and Hazes *et al.* both suggest that domain rotation directly impacts the interaction between Phe49 and coordinating histidines within the dinuclear copper site. Specifically, they describe how the aromatic ring of Phe49 packs tightly against the imidazole ring of His328 in the closed state, restricting His328's movement and preventing it from adopting the optimal position required for oxygen coordination. This restriction is lifted in the open conformation, allowing His328 to adopt a lower energy position, thereby facilitating increased oxygen affinity.^12,13^ However, our network analysis reveals a divergent behaviour in slipper limpet hemocyanin. Specifically, in the FU-a_oxy_ state, the corresponding residue, Phe67, forms persistent interactions with coordinating histidines His62, His184, and His215. Notably, the incidence of these interactions decreases by 40.3%, 46.1%, and 47.0%, respectively, upon deoxygenation (Fig. S3B†). This suggests that, in contrast to other species, Phe67 does not obstruct the histidines from adopting the energetically favourable conformation required for optimal oxygen binding. Rather it appears to play a role in stabilising the dicopper site in the oxygenated state, supporting the structural integrity of the active site without hindering oxygen coordination.

### Tunnel formation

To better understand how solvent accessibility near the active site varies between functional states, we conducted tunnel detection analysis to identify transient pathways that could facilitate oxygen diffusion. A summary of the detected tunnels is presented in Table 2, with the top-ranked cluster identified as the most probable candidate based on its higher throughput and average bottleneck radius. The analysis revealed a pronounced disparity in tunnel formation between the two functional states. The FU-a_deoxy_ system exhibited a 13.6-fold increase in tunnel frequency, with 776 tunnels detected across the simulation ensemble, compared to just 57 tunnels in FU-a_oxy_. This highlights a clear bias in tunnel permeability towards the deoxygenated state.

**Table 2:** Tunnel analysis of FU-a_oxy_ and FU-a_deoxy_ states performed using Caver 3.0 with a probe radius of 1.5 Å and clustering threshold of 3.0 Å. Bottleneck radius (BR) and throughput (TP) values describe tunnel size and accessibility

ID	No_snaps	Avg_BR	SD	Max_BR	Avg_L	SD	Avg_thru	SD
**FU-aoxy**								
1	27.80	1.53	0.03	1.62	15.94	1.50	0.63	0.03
2	24.20	1.53	0.03	1.59	20.57	1.61	0.56	0.03
3	17.00	1.55	0.04	1.63	24.79	2.84	0.51	0.05
4	14.25	1.53	0.02	1.57	29.69	4.83	0.46	0.04
5	7.00	1.55	0.01	1.58	37.01	1.48	0.40	0.01

**FU-a**_**deoxy**_								
1	980.00	1.58	0.07	1.90	17.24	2.79	0.61	0.05
2	875.75	1.57	0.06	1.85	17.90	2.29	0.60	0.04
3	696.25	1.56	0.06	1.82	23.31	2.80	0.53	0.04
4	599.75	1.57	0.06	1.85	26.02	3.11	0.53	0.05
5	458.75	1.55	0.05	1.77	34.88	3.73	0.45	0.05

**Fig. 5:**
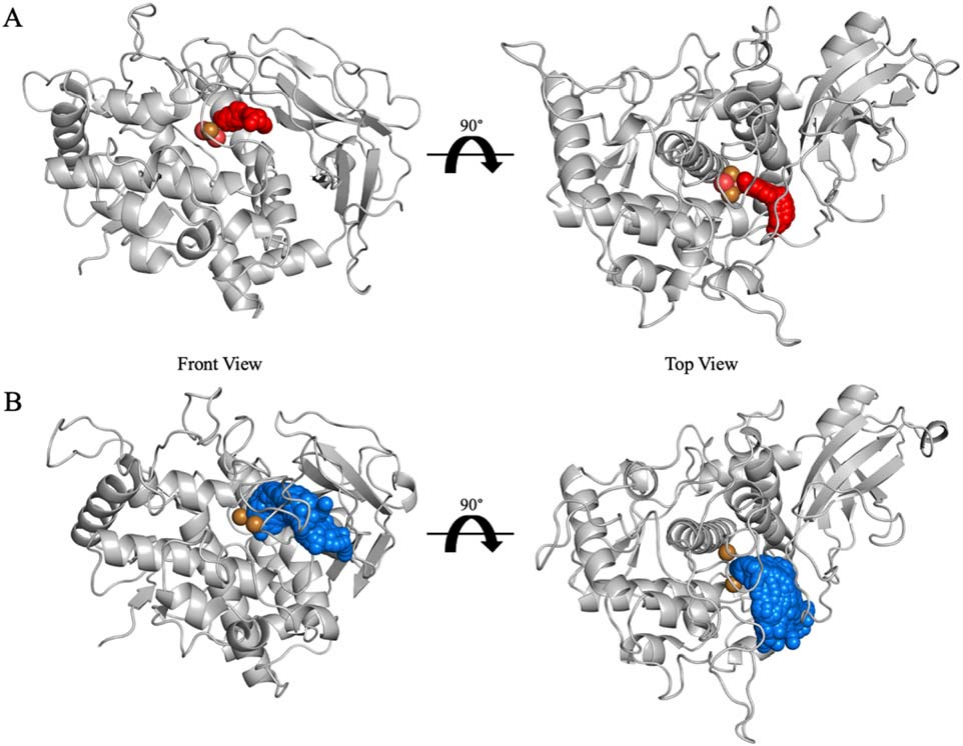
Tunnel analysis in FU-a_oxy_ and FU-a_deoxy_. The highest-throughput tunnel clusters are identified and shown as red spheres in (A) FU-a_oxy_ and blue spheres in (B) FU-a_deoxy_. Minimum bottleneck radius was defined at 1.5 Å.

Bottleneck analysis, which quantifies the narrowest point along the tunnel, showed comparable mean values for each functional states: 1.54 Å for FU-a_oxy_ and 1.57 Å for FU-a_deoxy_, both of which are theoretically sufficient for oxygen transport. However, in FU-a_deoxy_, the bottleneck radii were observed to be more variable, with some tunnels exhibiting radii exceeding 1.9 Å. In contrast FU-a_oxy_ showed little deviation from the mean, with the maximum bottleneck radius observed at 1.62 Å. These results, characterised by a notable increase in tunnel formation frequency and larger cavities in the deoxygenated state, underscores the inherent favourability of FU-a_deoxy_ for oxygen import relative to FU-a_oxy_ (Fig. 5).

Given the crucial role of channel bottlenecks in controlling access to the active site, we next focused on identifying the residues that contribute to bottleneck formation and their variability across the functional states. By analysing the top-ranked tunnel clusters for both FU-a_oxy_ and FU-a_deoxy_, we were able to pinpoint residues that consistently participate in bottleneck constriction, highlighting those most influential in regulating tunnel permeability. Residues that appeared in ≥ 70% of tunnel snapshots were considered crucial, providing a robust basis for identifying key structural determinants of channel accessibility.

The analysis revealed distinct bottleneck profiles between the two functional states. In FU-a_oxy_, where the tunnel adopts a more constricted conformation, the bottleneck region is primarily shaped by residues His62, Leu202, Leu347, and Glu352. Contrastingly, FU-a_deoxy_ displayed a substantially altered residue composition at the bottleneck. While His62 and Leu202 remained involved in both states, the contributions of Leu347 and Glu352 were markedly reduced in the deoxygenated state. Notably, Trp355 emerged as a key residue for tunnel constriction in FU-a_deoxy_, contributing to 71.11% of observed tunnel snapshots.

The consistent involvement of His62 and Leu202 across both states is unsurprising, given His62's role in copper coordination and Leu202's proximity to the active site. These residues, however, likely contribute to constriction in an accessory role, supporting but not directly controlling oxygen gating. In contrast, while Leu347 is positioned near the active site, its internal orientation directs the flow within the cavity rather than obstructing it, making its role in directly mediating oxygen import unlikely. Trp355, on the other hand, occupies a position where its steric bulk could block oxygen entry, with the indole sidechain large enough to potentially obstruct the channel. However, the lack of contribution in FU-a_oxy_ bottlenecks suggests that Trp355 is not crucial for blocking oxygen entry when oxygen is already bound, supporting the idea that its involvement is primarily linked to the deoxygenated state.

Glu352 was the final residue implicated in bottleneck formation in FU-a_oxy_. Like Trp355, it resides on a dynamic loop near the active site but is located on the exterior of the protein, where its carboxyl sidechain may sterically obstruct the channel, potentially limiting oxygen entry. The contribution of Glu352 to the bottleneck is notable, highlighting a minor decrease upon deoxygenation. This reduction is consistent with the expected channel expansion in the deoxygenated state, allowing for greater variability in the residues that contribute to the bottleneck as the tunnel widens. Hazes *et al.* previously proposed that glutamic acid residues function as a molecular “shutter”, regulating oxygen entry in *Limulus polyphemus*, with particular emphasis on the critical role of the negative charge.^12^ To assess the conservation of this mechanism, we performed sequence alignments of the functional units from slipper limpet hemocyanin, keyhole limpet hemocyanin, and octopus hemocyanin (FU-d only) (Table S7†). Additionally, we conducted a broader alignment encompassing all molluscan hemocyanin sequences (Table S8†). Our results indicate that Glu352 was fully conserved across all sequences (Table S6†), underscoring its critical, evolutionary conserved, role in regulating oxygen transport.

Mapping the detected tunnels onto the didecameric hemocyanin structure further contextualises their functional significance. The tunnel is positioned on the protein's solvent-exposed exterior, placing it in direct communication with the surrounding environment. Such an arrangement would optimise access for oxygen uptake and release, supporting the proposed dynamic gating mechanism inferred from residue contributions. Nevertheless, whether this tunnel trajectory and gating mechanism are conserved across all functional units within the oligomer remains unclear and warrants further investigation. Taken together, these findings establish a mechanistic link between active site dynamics, tunnel formation, and residue-specific gating in molluscan hemocyanins, providing new insights into how oxygen access is precisely regulated in response to functional state.

### Driving forces of oxygen release

Our structural analyses of FU-a reveal distinct conformational differences between oxygenated and deoxygenated states, yet the exact mechanism of oxygen release remains unclear, especially given the stability of the oxygen-bound form. Existing evidence supports our findings, indicating that oxygen release in molluscan hemocyanins involves an increase in the Cu–Cu distance at the binuclear copper center.^12,13^ However, these local changes are modulated by allosteric regulation within the multimeric assembly, governed by the surrounding ionic environment.^62–65^

Since oxygen binding restricts flexibility and solvent accessibility within FU-a, dissociation is ultimately driven by external physiological cues — namely, chloride-mediated inhibition of domain rearrangement, acidification, and calcium-dependent stabilisation — that trigger quaternary level rearrangements and transitions.^63,66,67^ These higher-order conformational dynamics enable the cooperative exchange between oxygenated and deoxygenated states. Consequently, a comprehensive understanding of oxygen release necessitates investigation at the multimeric level, as single-unit analyses cannot fully capture the mechanisms underlying allosteric regulation.

### Network analysis

**Fig. 6:**
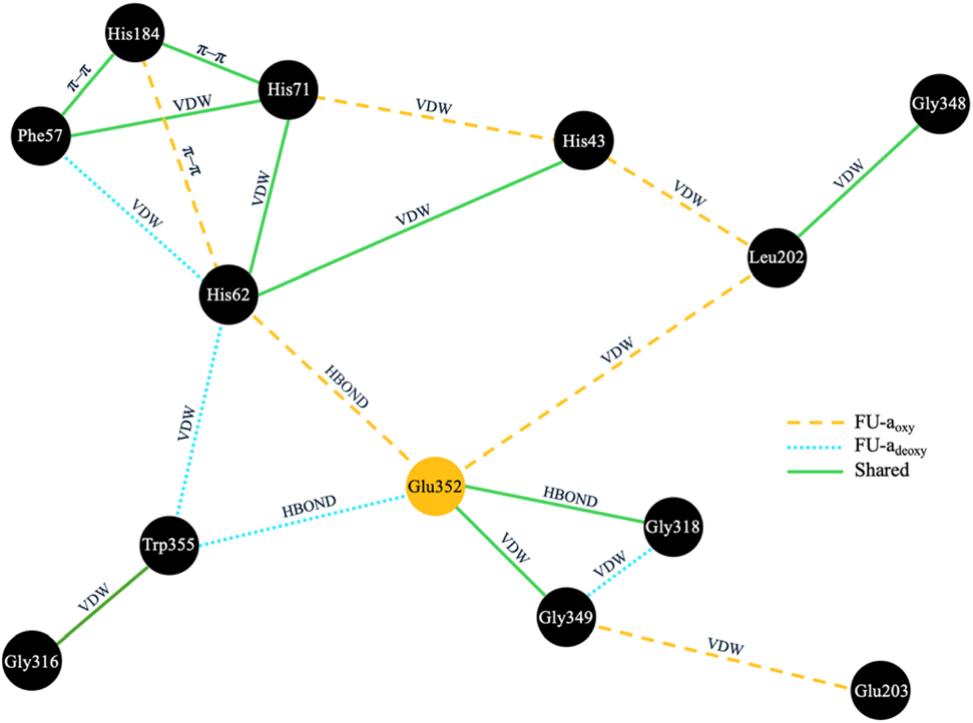
Residue interaction network (RIN) depicting short-range connectivity between coordinating histidines and Glu352 in FU-a_oxy_ and FU-a_deoxy_. Interaction types are annotated, with the colour and style of connecting line indicating if the interaction is unique to FU-a_oxy_ (orange), FU-a_deoxy_ (blue), or shared (green). Interaction types include van der Waals (VDW), hydrogen bonds (HBONDS), pi–pi stacking (π–π) stacking, ionic interactions (IONIC), and π–cation interactions (PICATION).

Given the strong evolutionary conservation and functional importance of Glu352 in regulating tunnel access, we next sought to investigate the structural pathways through which oxygen binding could modulate its conformation. To achieve this, we constructed residue interaction networks (RINs) from molecular dynamics simulations, aiming to map potential signal propagation routes from the oxygen-binding site to Glu352 and identify residues that mediate these transitions (Fig. 6).

RINs were generated using RING 4.0, with every 10th frame sampled from each trajectory to capture the dynamic ensemble. Interaction probabilities were then averaged across replicates to produce consensus networks for each functional state. Given the close spatial proximity of Glu352 to the active site, we hypothesised that signal transmission would likely occur *via* direct or near–direct interactions. Accordingly, we applied a stringent interaction strength threshold (≥0.3) and restricted pathway lengths to a maximum of two residues from Glu352. This focused strategy enabled us to isolate the most plausible short-range communication routes coupling oxygen binding to tunnel gating.

Subsequent analysis revealed distinct signal propagation networks associated with the oxygenated and deoxygenated states (Fig. 6). In FU-a_oxy_, Glu352 is stably coordinated within a network dominated by active site residues His62 and Leu202, as well as loop residues Gly318 and Gly349. This core interaction network is further reinforced by secondary contacts, positioning Glu352 as a central hub within the structural framework. Upon deoxygenation, substantial rearrangements are observed: interactions linking Leu202, His62, His43, and Glu352 are disrupted, resulting in increased flexibility of Glu352, as indicated by elevated RMSF values (Fig. S1†).

To assess how Glu352 dynamics influence tunnel accessibility, we calculated the heavy-atom RMSD of its side chain relative to the starting conformation across structural ensembles of the oxygenated and deoxygenated states, and analysed rotamer distributions (Fig. S2†). In FU-a_oxy_, Glu352 adopts two primary rotamers that retain a consistent orientation relative to His62, with only minor shifts in the carboxyl group (Fig. S4†). As a result, the Glu352 side chain remains conformationally constrained, and the tunnel remains blocked in both cases. In contrast, the deoxygenated state shows greater conformational variability. While the high-RMSD conformation preserves contacts with His62 and occludes the tunnel, the low-RMSD conformation shows a marked reorientation—Glu352 disengages from His62 and redirects its carboxyl group into the active site cavity, permitting tunnel opening. This conformational flexibility is absent in FU-a_oxy_, indicating that oxygen binding triggers Cu–Cu association, which in turn drives the repositioning of His62. This movement of His62 is fundamental for stabilising Glu352 in a conformation that blocks the tunnel (Fig. S4†). Deoxygenation disrupts this coordination network, allowing Glu352 to adopt alternative conformations that transiently open the tunnel, facilitating oxygen diffusion into the active site.

To assess the conservation of this mechanism, we revisited sequence alignments performed across slipper limpet hemocyanin, keyhole limpet hemocyanin, and octopus hemocyanin (FU-d only), as well as a broader dataset of molluscan hemocyanins (Tables S7 and S8†). Unsurprisingly, key components of the rigid coordination network including His43, His62, and Leu202, are fully conserved across all functional units and molluscan sequences, supporting their essential structural roles (Table S6†). Gly349 and Trp355 are likewise fully conserved at the broader molluscan level; however, among the functional units analysed, occasional substitutions of Trp355 with phenylalanine and Gly349 with aspartate are observed. Given their respective BLOSUM62 log-odds scores of +1 and −1, these substitutions are considered conservative and unlikely to compromise network integrity. In contrast, Gly318 exhibits considerable sequence variability across both molluscan hemocyanins and functional units. This divergence suggests that, while Gly318 participates in the network, its contribution likely involves backbone-mediated rather than sidechain-specific interactions. Nevertheless, the overall high degree of conservation within this short signal propagation pathway strongly supports its functional relevance, and suggests that the mechanism of oxygen gating through Glu352-centered network rearrangement is a broadly conserved feature across molluscan hemocyanins.

## Conclusions

This study presents a comprehensive investigation into the structural dynamics underpinning oxygen binding and transport in FU-a of slipper limpet hemocyanin. Our analysis revealed that oxygenation led to an overall increase in structural rigidity, a decrease in solvent accessibility and a constriction of the oxygen tunnel, suggesting that oxygen binding increases protein compactness and restricts flexibility. We identified that deoxygenation triggers conformational changes leading to an increase in the flexibility of the conserved gatekeeper residue Glu352, enhancing solvent accessibility to the active site and facilitating oxygen capture. Together, our findings provide new mechanistic insights into how hemocyanins regulate oxygen binding through dynamic modulation of tunnel formation and gating, and underscore the critical role of conserved residues such as Glu352 in the facilitation of oxygen capture.

## Author contributions

JGD was involved in investigation, formal analysis, methodology, writing – original draft and visualisation. JAP was involved in methodology, supervision and writing – review and editing. MTY was involved in conceptualisation, visualization and writing – original draft and review and editing. GEM was involved in conceptualisation, methodology, supervision and writing – review and editing.

## Conflicts of interest

M. T. Y. has previously been supported by funding from Innovate UK (Technology Strategy Board grants 69001 and 36295). The funders had no role in study design, data collection and analysis, decision to publish, or preparation of the manuscript.

## Supplementary Material

RA-015-D5RA03248B-s001

## Data Availability

Data supporting this article, including pdb, topology and trajectory files for both oxy and deoxy hemocyanin are available at https://doi.org/10.5281/zenodo.15350967.
