# Supplementary material for: Increased functional unit flexibility and solvent accessibility favours oxygen capture in molluscan hemocyanin
Source: RSC Adv. 2025 Jun 16;15(26):20373–84. doi: 10.1039/d5ra03248b (PMC12169132; doi:10.1039/d5ra03248b)
Supplement: RA-015-D5RA03248B-s001 [file RA-015-D5RA03248B-s001.pdf]

Supplementary Information (SI) for RSC Advances.
This journal is © The Royal Society of Chemistry 2025

Table S1. Force constants and equilibrium distances of coordinating atoms to metal centres, as calculated from B3LYP/ Def2-TZVP optimisation of the metal sites.

State	Ligating Bond	Force constant (kCal/mol·Å^{-2})	Equilibrium Distance (Å)
FU-a$_{oxy}$	HIS43 (N) – CU2	90.1	1.990
	HIS62 (N) – CU2	82.7	1.993
	HIS71 (N) – CU2	7.4	2.372
	HIS184 (N) – CU1	93.1	1.984
	HIS188 (N) – CU1	89.3	1.982
	HIS215 (N) – CU1	11.5	2.352
FU-a$_{deoxy}$	HIS43 (N) – CU2	49.9	2.014
	HIS62 (N) – CU2	50.6	2.011
	HIS71 (N) – CU2	49.3	2.018
	HIS184 (N) – CU1	50.4	2.013
	HIS188 (N) – CU1	49.0	2.019
	HIS215 (N) – CU1	50.4	2.012

Table S2. Angles of the metal ion centres and ligating atoms, including force constants, calculated using B3LYP/ Def2-TZVP.

Oxygenation State	Ligating atoms	Force constant (kCal/mol·rad^{-2})	Angle (°)
FU-a$_{oxy}$	HIS43 (N) – CU2 – HIS62 (N)	102.61	97.22
	HIS43 (N) – CU2 – HIS71 (N)	32.44	91.30
	HIS62 (N) – CU2 – HIS71 (N)	44.07	93.73
	HIS184 (N) – CU1 – HIS188 (N)	100.00	97.30
	HIS184 (N) – CU1 – HIS215 (N)	25.83	91.99
	HIS188 (N) – CU1 – HIS215 (N)	42.50	93.51
FU-a$_{deoxy}$	HIS43 (N) – CU2 – HIS62 (N)	2.87	121.86
	HIS43 (N) – CU2 – HIS71 (N)	2.96	118.16
	HIS62 (N) – CU2 – HIS71 (N)	2.98	119.98
	HIS184 (N) – CU1 – HIS188 (N)	2.99	118.33
	HIS184 (N) – CU1 – HIS215 (N)	2.86	122.09
	HIS188 (N) – CU1 – HIS215 (N)	2.95	119.58

Table S3. Mean equilibrium distances and associated variance of coordinating distances from ligating atoms to metal centres, as calculated from molecular dynamic trajectories.

Oxygenation State	Ligating Bond	Equilibrium Distance (Å)	SD (±)
FU-a_{oxy}	HIS43 (N) – CU2	2.03	0.06
	HIS62 (N) – CU2	2.00	0.06
	HIS71 (N) – CU2	2.49	0.15
	HIS184 (N) – CU1	1.97	0.05
	HIS188 (N) – CU1	2.01	0.05
	HIS215 (N) – CU1	2.44	0.14
FU-a_{deoxy}	HIS43 (N) – CU2	2.40	0.14
	HIS62 (N) – CU2	2.33	0.08
	HIS71 (N) – CU2	2.20	0.02
	HIS184 (N) – CU1	2.20	0.02
	HIS188 (N) – CU1	2.27	0.15
	HIS215 (N) – CU1	2.20	0.02

Table S4. Mean binding free energy (ΔG_{bind}) values for FU-a_{deoxy} and FU-a_{oxy} systems calculated using MM/GBSA across internal dielectric constant values ranging from 1 to 5.

Oxygenation State	IDC	ΔG_{bind} (kcal mol^{-1})	SD (±)
FU-a_{oxy}	1	-72.77	1.98
	2	-38.36	1.56
	3	-26.89	1.44
	4	-21.16	1.38
	5	-17.72	1.35
FU-a_{deoxy}	1	-0.92	3.04
	2	-1.15	3.04
	3	-1.23	3.04
	4	-1.27	3.04
	5	-1.29	3.04

Table S5. Mean binding free energy (ΔG_{bind}) values for FU-a_{deoxy} and FU-a_{oxy} systems calculated using MM/PBSA across internal dielectric constant values ranging from 1 to 5.

Oxygenation State	IDC	ΔG_{bind} (kcal mol^{-1})	SD (±)
FU-a_{oxy}	1	-80.31	2.112
	2	-42.53	1.649
	3	-29.93	1.514
	4	-23.63	1.451
	5	-19.85	1.415
FU-a_{deoxy}	1	-4.29	2.986
	2	-4.60	3.003
	3	-4.70	3.009
	4	-4.75	3.012
	5	-4.78	3.014

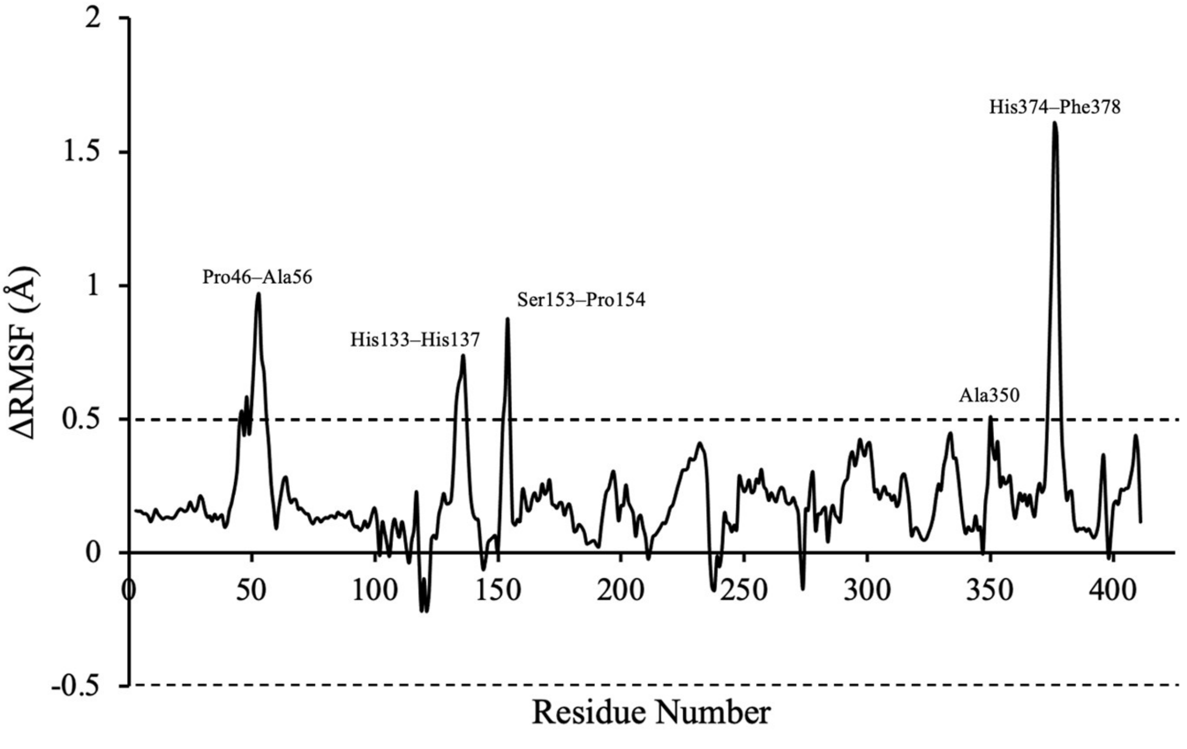

Figure S1. Per-residue differences in root-mean-square fluctuation (RMSF) between FU-adeoxy and FU-aoxy systems, calculated as ΔRMSF (FU-a_{deoxy} – FU-a_{oxy}). Residues with absolute RMSF differences exceeding ±0.5 Å are highlighted. The dotted line denotes the ±0.5 Å threshold used to define significant deviations. Major fluctuations are observed in labelled regions.

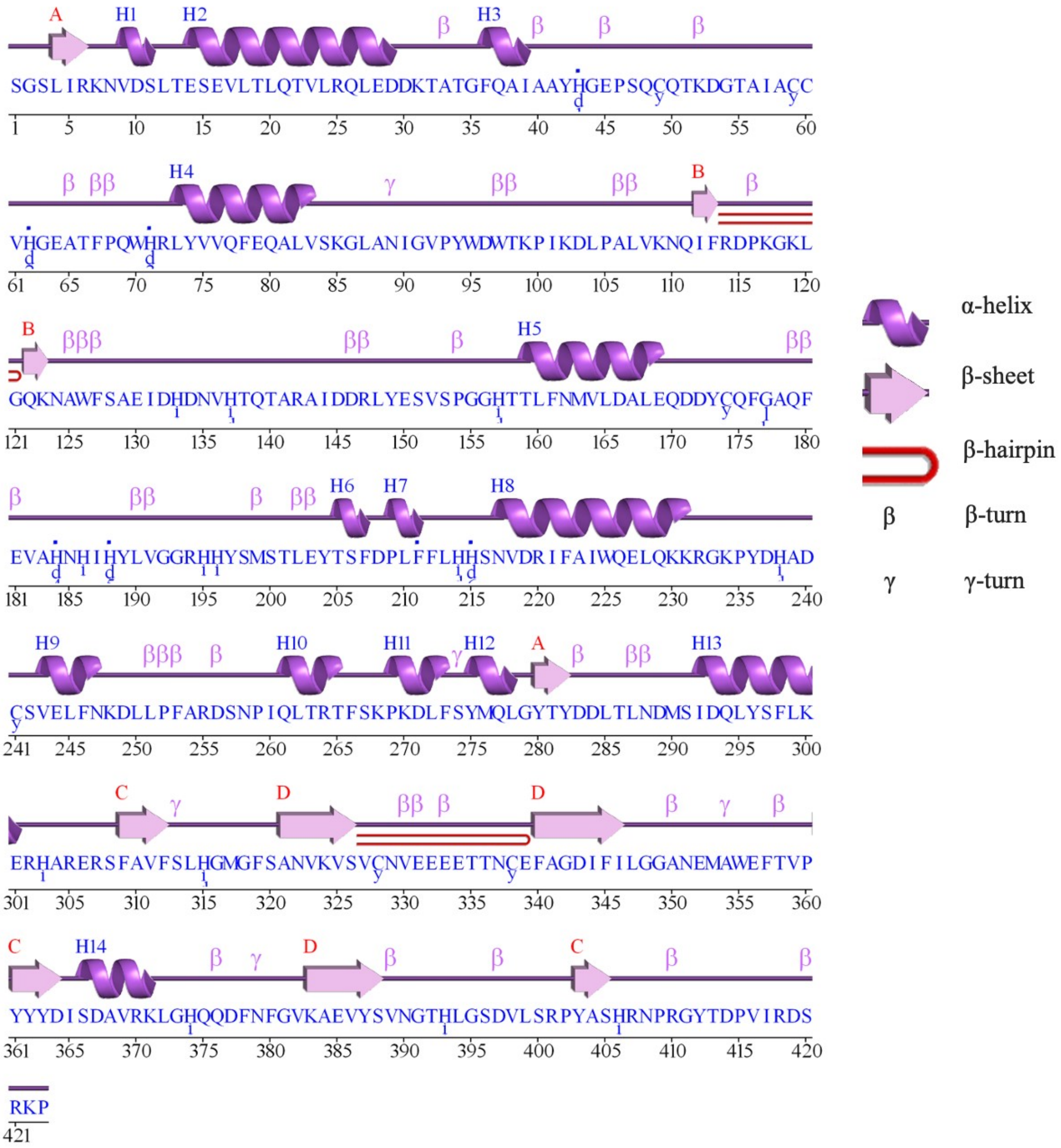

Figure S2. Two-dimensional secondary structure map of the FU-a hemocyanin domain. The structure is divided into two domains: Domain 1 (residues 1–305) and Domain 2 (residues 306–423), as indicated along the sequence. α-helices are depicted as coils, β-sheets as arrows, β-hairpins as loops, and β-turns (β) and γ-turns (γ) are explicitly labelled.

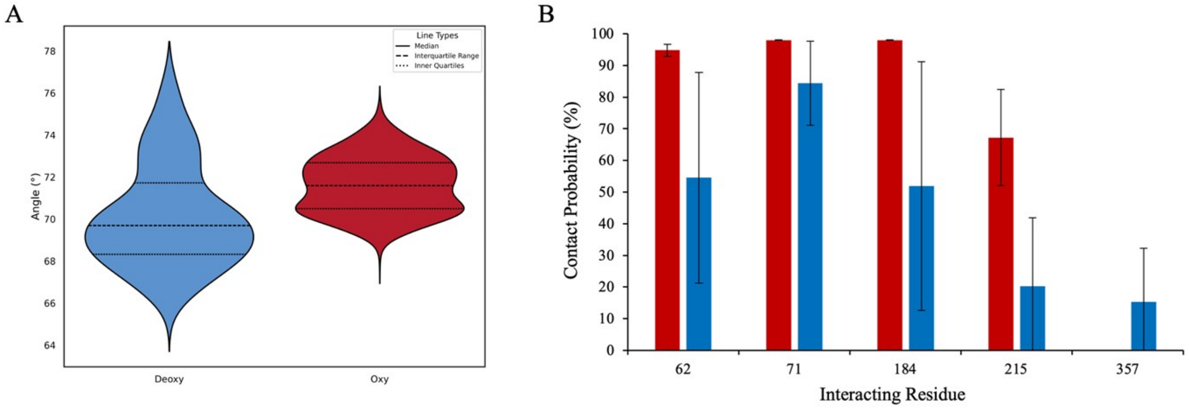

Figure S3. Structural and Functional Insights into Domain Rotation and Phe67 Residue Interactions. (A) Violin plot illustrating the inward rotation of domain II relative to domain I in the oxygenated (FU-aoxy) and deoxygenated (FU-adeoxy) states. (B) Interaction data showing the π–π stacking interactions of Phe67 with coordinating histidines, highlighting the differences between the oxygenated (FU-a$_{oxy}$, red) and deoxygenated (FU-a$_{deoxy}$, blue) states.

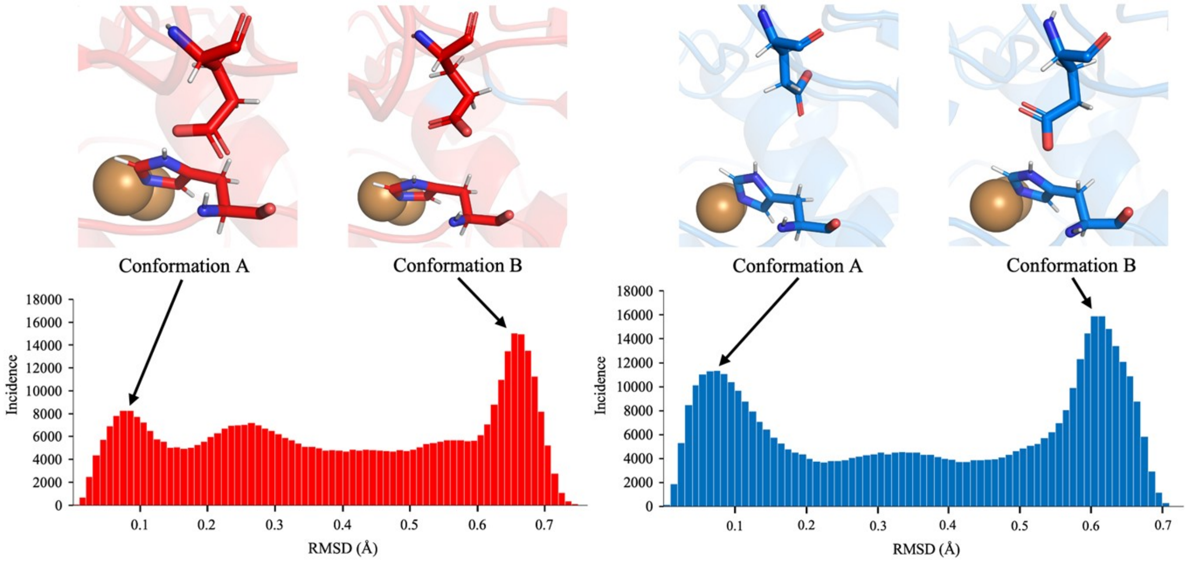

Figure S4. Comparison of Glu352 side-chain conformations in FU-a$_{oxy}$ (red) and FU-a$_{deoxy}$ (blue). Conformation A (low RMSD) and Conformation B (high RMSD) represent the dominant states identified from side-chain heavy-atom RMSD analysis.

Table S6. Sequence conservation in the interaction network shown in Figure 6. *From visual inspection of the 3D structure alignment of octopus (PDB 1JS8) and horseshoe crab (PDB 1OXY) hemocyanin.

Amino acid residue	Conservation in functional units of SLH and KLH	Conservation in FU-a of molluscan hemocyanins	Notes
His43	Full	Full	Coordinates Cu1
His62	Full	Full	Coordinates Cu1
Phe67	Full	Full	Structurally conserved in horseshoe crab*
His71	Full	Full	Coordinates Cu1
His184	Full	Full	Coordinates Cu2
Leu202	Full	Full	
Glu203	Glu, Asp, His, Arg	Glu or Asp	
Gly316	No	No	
Gly318	No	No	
Gly348	Full	Full	
Gly349	Gly, Asp	Full	Asp in FU-c only
Glu352	Full	Full	
Trp355	Trp, Phe	Full	Phe in FU-d only

Table S7. Protein sequence alignment (Clustal Omega) of the eight functional units (FUa – FUh) of slipper limpet hemocyanin (*Crepidula fornicata*; SLH1; GenBank accession number WCA44164.1) and keyhole limpet hemocyanin (*Megathura crenulata*; MecrH1; UniProtKB/Swiss-Prot accession number Q10583.2), along with the protein sequence of *Enteroctopus dofleini* Odg from PDB 1JS8. FUa of SLH is shown in bold, and the key residues participating in the interaction network shown in Figure 6 are highlighted in yellow.

```
SLH1_FUh        ---------------TGDVTRYEVSALSLAQVTNLRDALYKLQNDHGPNGFEAIASFHGA    45
MecrH1_FUh      ---------------EDILVRKNIHSLSHHEAEELRDALYKLQNDESHGGYEHIAGFHGY    45
SLH1_FUa        ---------------SGSLIRKNVDSLTESEVLTLQTVLRQLEDDKTATGFQAIAAYHGE    45
MecrH1_FUa      MLSVRLLIVVLALANAENLVRKSVEHLTQEETLDLQAALRELQMDSSSIGFQKIAAAHGA    60
SLH1_FUd        ------------EVQASSHVRRNLEDLTTGEAESLRSALRDMEEDG---SFDAIARFHGY    45
MecrH1_FUd      ------------EVTSANRIRKNIENLSLGELESLRAAFLEIENDG---TYESIAKFHGS    45
SLH1_FUf        -------------EVAPNRIRHDLTHLSERDIRSLKSSIRDLQLDDSNDGYQNIASFHGA    47
MecrH1_FUf      -------------HLSLNKVRHDLSTLSERDIGSLKYALSSLQADTSADGFAAIASFHGL    47
SLH1_FUg        --------------IAGVGVRKDINTLTAAEMENLRDALGRVQAGTGRLTYDNVVSAHGY    46
OcdoH1          -----------------AIIRKNVNSLTPSDIKELRDAMAKVQADTSDNGYQKIASYHGI    43
MecrH1_FUg      --------------MAGHGVRKEINTLTTAEVDNLKDAMRAVMADHGPNGYQAIAAFHGN    46
SLH1_FUb        ----------------SNVVRKDIELLTDEEVYALRQAMHRFQNDTSIDGYQAVAEFHGL    44
MecrH1_FUb      -----------------------INDLTREEVLNLREAFHKFQEDRSVDGYQATAEYHGL    37
SLH1_FUc        --------------EDDDHVRNDIVTLTAEQEQNVRDAFQGLKNDKSVAGYNQIAAFHGQ    46
MecrH1_FUc      --------------PRSRLIRKNVDRLSPEEMNELRKALALLKEDKSAGGFQQLGAFHGE    46
SLH1_FUe        -----------------RLVRKSVLSLSPAERRSLVLAMRSLQEDSSADGFQSLASFHAL    43
MecrH1_FUe      -----------------VMIRKDITQLDKRQQLSLVKALESMKADHSSDGFQAIASFHAL    43
                                       :  *   :   :   :  .  .     :      *.

SLH1_FUh        PGLCPENA-TDHYACCRHGMPAFPHWHRLLTVQFERALKDKG-AVVGVPYWDWTRPAKAM    103
MecrH1_FUh      PNLCPEKG-DEKYPCCVHGMSIFPHWHRLHTIQFERALKKHG-SHLGIPYWDWTQTISSL    103
SLH1_FUa        PSQCQTKDGT-AIACCVHGEATFPQWHRLYVVQFEQALVSKGLANIGVPYWDWTKPIKDL    104
MecrH1_FUa      PASCVHKD-T-SIACCIHGMPTFPHWHRAYVVHMERALQTKR-RTSGLPYWDWTEPITQL    117
SLH1_FUd        PGLCEHD-GH-KAACCVHGSPAFPHWHRLYVEQVENSLLSHG-SAVSVPYWDWTQPIKKL    102
MecrH1_FUd      PGLCQLN-GN-PISCCVHGMPTFPHWHRLYVVVVENALLKKG-SSVAVPYWDWTKRIEHL    102
SLH1_FUf        PALCPSPEAA-EYACCVHGMPTFPHWHRLYTVEMEDAMVRHG-SSVALPYWDWTMPITQL    105
MecrH1_FUf      PAKCNDSHNN-EVACCIHGMPTFPHWHRLYTLQFEQALRRHG-SSVAVPYWDWTKPIHNI    105
SLH1_FUg        PPQCTH-DGH-KVACCQHGMASFPMWHRLFTRQMEVALSWEG-AKVGIPYWDWTEAFTEL    103
OcdoH1          PLSCHYENGT-AYACCQHGMVTFPNWHRLLTKQMEDALVAKG-SHVGIPYWDWTTTFANL    101
MecrH1_FUg      PPMCPMPDGK-NYSCCTHGMATFPHWHRLYTKQMEDALTAHG-ARVGLPYWDGTTAFTAL    104
SLH1_FUb        PAKCPHPDAAVRYACCVHGMATFPHWHRLFVVEVEDELRSRG-LEIGIPYWDWTRPNAHI    103
MecrH1_FUb      PARCPRPDAKDRYACCVHGMPIFPHWHRLFVTQVEDALVGRG-ATIGIPYWDWTEPMTHI    96
SLH1_FUc        PNWCPSTQAERKYACCTHGMAVFPHWHRLLTVQAENGLIANG-LHSGLPYWDWTLPMTSL    105
MecrH1_FUc      PKWCPSPEASKKFACCVHGMSVFPHWHRLLTVQSENALRRHG-YDGALPYWDWTSPLNHL    105
SLH1_FUe        PPLCPYPEATKRFACCIHGMATFPHWHRLYTVQFEDALRRHG-ALVGIPYWDTVVPNSRL    102
MecrH1_FUe      PPLCPSPAASKRFACCVHGMATFPQWHRLYTVQFQDSLRKHG-AVVGLPYWDWTLPRSEL    102
                *  *          ** **   ** ***  .   :  :  .     .:**** .     :

SLH1_FUh        PSLFTDSY---------DNNPFQTYRMTFNDQY--IQRDV-SEELFNHPSEGDVESLFHQ    151
MecrH1_FUh      PTFFADSG---------NNNPFFKYHIRSINQD--TVRDV-NEAIFQQTKFGEFSSIFYL    151
SLH1_FUa        PALVKNQIFRDPKGKLGQKNAWFSAEIDHDNVHTQTARAI-DDRLYESVSPGGHTTLFNM    163
MecrH1_FUa      PSLAADPVYIDSQGGKAHTNYWYRGNIDFL--DKKTNRAV-DDRLFEKVKPGQHTHLMES    174
SLH1_FUd        PALINDATYYDSRAHAKLENPFFRWKIPGTDMY--TSRDP-RPDLFDS------DYFLNN    153
MecrH1_FUd      PHLISDATYYNSRQHHYETNPFHHGKITHENEI--TTRDP-KDSLFHS------DYFYEQ    153
SLH1_FUf        PDLFTSETYYDAWRDEVVANPFARAYIKVAGGY--TVRDP-QAQLKQLSRDGQHSALFDL    162
MecrH1_FUf      PHLFTDKEYYDVWRNKVMPNPFARGYVPSHDTY--TVRDV-QEGLFHLTSTGEHSALLNQ    162
SLH1_FUg        PALVREEE----------NNPFHHGRIPGTDTV--TTRAP-RPQLFRDPEHGDESFFYRQ    150
OcdoH1          PVLVTEEK----------DNSFHHAHIDVANTD--TTRSP-RAQLFDDPDKGDKSFFYRQ    148
MecrH1_FUg      PTFVTDEE----------DNPFHHGHIDYLGVD--TTRSP-RDKLFNDPERGSESFFYRQ    151
SLH1_FUb        PALANDVSYEDANTHQQLHNPFHDAPIAFLGE--KTTRQV-QDDLSESPEFGDHTSLFDG    160
MecrH1_FUb      PGLAGNKTYVDSHGA-SHTNPFHSSVIAFEENAPHTKRQI-DQRLFKPATFGHHTDLFNQ    154
SLH1_FUc        PSIVLNATYVHPKTGESVANPLYSGQVDGHD----TTRSV-RQELFEQPKFGHMTKIAEK    160
MecrH1_FUc      PELADHEKYVDPEDGVEKHNPWFDGHIDTVDKT--TTRSV-QNKLFEQPEFGHYTSIAKQ    162
SLH1_FUe        PDFIAESVWDDPLFHANFSNPWAGADIEFDNSAV--VRDVNMDRISQKGPKGYDTWSWKQ    160
MecrH1_FUe      PELLTVSTIHDPETGRDIPNPFIGSKIEFEGENVHTKRDINRDRLFQGSTKTHHNWFIEQ    162
                * :                *      :          *      :

SLH1_FUh        ALETLEETNYCDFEVQYEMLHNAVHELIGGGNTYSMSTLEYSAFDPFFMVHHASIDRIWQ    211
MecrH1_FUh      ALQALEEDNYCDFEVQYEILHNEVHALIGGAEKYSMSTLEYSAFDPYFMIHHASLDKIWI    211
SLH1_FUa        VLDALEQDDYCQFEAQFEVAHNHIHYLVGGRHHYSMSTLEYTSFDPLFFLHHSNVDRIFA    223
MecrH1_FUa      VLDALEQDEFCKFEIQFELAHNAIHYLVGGKHDYSMANLEYTAYDPIFFLHHSNVDRIFA    234
SLH1_FUd        ALLALEQTSYCDFEVQFEILHNALHSFLGGRGKYSLSSLDYSAFDPVFFLHHANVDRIWA    213
MecrH1_FUd      VLYALEQDNFCDFEIQLEILHNALHSLLGGKGKYSMSNLDYAAFDPVFFLHHATTDRIWA    213
SLH1_FUf        VLLVLEQTDYCDFEVQFEVVHNAIHYLVGGRQLYSMSSLEYTSYDPIFFVHHSFVDKIWA    222
MecrH1_FUf      ALLALEQHDYCDFAVQFEVMHNTIHYLVGGPQVYSLSSLHYASYDPIFFIHHSFVDKVWA    222
SLH1_FUg        VQLALEQRDYCDFEVQFEVIHNAIHSWIGGTSPYGMSTLEYSAYDPVFFIHHSNVDRQFA    210
```

```
OcdoH1          IALALEQTDFCDFEIQFEIGHNAIHSWVGGSSPYGMSTLHYTSYDPLFYLHHSNTDRIWS     208
MecrH1_FUg      VLLALEQTDFCQFEVQFEITHNAIHSWTGGLTPYGMSTLEYTTYDPLFWLHHANTDRIWA     211
SLH1_FUb        ILLAFEQTDFCDFEVQFEVVHNAPHFLVGGFGAYTLATLHYSAFDPIFYLHHSNVDRLWA     220
MecrH1_FUb      ILYAFEQEDYCDFEVQFEITHNTIHAWTGGSEHFSMSSLHYTAFDPLFYFHHSNVDRLWA     214
SLH1_FUc        VMLAFEQDNFCDFEIQYEIAHNYIHALVGGNETYSMASLRYTAYDPIFFLHHSNTDRIWA     220
MecrH1_FUc      VLLALEQDNFCDFEIQYEIAHNYIHALVGGAQPYGMASLRYTAFDPLFYLHHSNTDRIWA     222
SLH1_FUe        YLYALEQENFCDFEVQFEIAHNALHAWMGGSEVHSMAHLHYASYDPVFILHHSNTDRIFA     220
MecrH1_FUe      ALLALEQTNYCDFEVQFEIMHNGVHTWVGGKEPYGIGHLHYASYDPLFYIHHSQTDRIWA     222
                   .:*:  .:*.*   *  *:  **   *    **    .  :.  * *:::** *  .**:   *:  :

SLH1_FUh        IWQSLQKLRHKPFNYASCASRSLYKPLEPFSYTSL-NADPLTLNNAQPVHIFDTA-KFHY     269
MecrH1_FUh      IWQELQKRRVKPAHAGSCAGDIMHVPLHPFNYESV-NNDDFTRENSLPNAVVDSH-RFNY     269
SLH1_FUa        IWQELQKKRGKPYDHADCSVELFNKDLLPFARDSN--PIQLTRTFSKPKDLFSYM-QLGY     280
MecrH1_FUa      IWQRLQELRNKDPKAMDCAQELLHQKMEPFSWEDN--DIPLTNEHSTPADLFDYC-ELHY     291
SLH1_FUd        IWQALQKIRGLPFDESDCALNIMGTPLHPFDDKEE-NQFDLTNKYSRPIDAFDYSNHFDY     272
MecrH1_FUd      IWQDLQRFRKRPYREANCAIQLMHTPLQPFDKSDN-ND-EATKTHATPHDGFEYQNSFGY     271
SLH1_FUf        VWQELQKRRGMNYDRADCAVNFMNERMHPFDWEAL-NPDVRTREHSLPQSVWKYE-DLGY     280
MecrH1_FUf      VWQALQEKRGLPSDRADCAVSLMTQNMRPFHYEIN--HNQFTKKHAVPNDVFKYE-LLGY     279
SLH1_FUg        IWQALQKYRGLDYNTANCNIQELRQVQEPFDRDDN--PVVTTRHYSKAIDAFNYD-QYGY     267
OcdoH1          VWQALQKYRGLPYNTANCEINKLVKPLKPFNLDTN--PNAVTKAHSTGATSFDYH-KLGY     265
MecrH1_FUg      IWQALQEYRGLPYDHANCEIQAMKRPLRPFSDPIN--HNAFTHSNAKPTDVFEYS-RFNF     268
SLH1_FUb        IWQQLQIKRGLPYK-AHCASSLTQEVLKPFGFPPPWNNDHKTFENARPTNIYDYEAVLDY     279
MecrH1_FUb      VWQALQMRRHKPYR-AHCAISLEHMHLKPFAFSSPLNNNEKTHANAMPNKIYDYENVLHY     273
SLH1_FUc        IWQALQKYRGKPYNSANCAIAEMRKPLQPFAQTSLTNPDYVTRDHSTPFDVFNYHSSFHY     280
MecrH1_FUc      IWQALQKYRGKPYNVANCAVTSMREPLQPFGLSANINTDHVTKEHSVPFNVFDYKTNFNY     282
SLH1_FUe        LWQELQKYRGRDANEVNCALELMKEPLKPFSFGSPYNLNPTTTLYSKPEDAFDYKGHFKY     280
MecrH1_FUe      IWQSLQRFRGLSGSEANCAVNLMKTPLKPFSFGAPYNLNDHTHDFSKPEDTFDYQ-KFGY     281
                :** **  *         *          **             *    :       .       :

SLH1_FUh        HYDSLDLNGHGVHELHDMIEDMQATSRIFAGFVLSGISTSARVHVDVTRGED--T-----     322
MecrH1_FUh      KYDNLNLHGHNIEELEEVLRSLRLKSRVFAGFVLSGIRTTAVVKVYIKSGTDSDD-----     324
SLH1_FUa        TYDDLTLNDMSIDQLYSFLKERHARERSFAVFSLHGMGFSANVKVSVCNVEEE--ETTNC     338
MecrH1_FUa      DYDTLNLNGMTPEELKTYLDERSSRARAFASFRLKGFGGSANVFVYVCIPDDNDRNDDHC     351
SLH1_FUd        HYDTLKFNSWTIPQLEQVLEKQRSRDRVFAGFLLHNIGTSADVEIDVCVATGNGA-KSCN     331
MecrH1_FUd      AYDNLELNHYSIPQLDHMLQERKRHDRVFAGFLLHNIGTSADGHVFVCLPTGEHT-KDCS     330
SLH1_FUf        HYDNYQIGGKSIEELEELIKKQQSHPRVFAGFQLHGLGTSADVELSVCKSQNSC------     334
MecrH1_FUf      RYDNLEIGGMNLHEIEKEIKDKQHHVRVFAGFLLHGIRTSADVQFQICKTSEDC------     333
SLH1_FUg        QYDNLNFHGMTISQLDEMLEKKKQEDHVFANFMLHGIQTSADVVFDLCDAKGKC------     321
OcdoH1          DYDNLNFHGMTIPELEEHLKEIQHEDRVFAGFLLRTIGQSADVNFDVCTKDGEC------     319
MecrH1_FUg      QYDNLRFHGMTIKKLEHELEKQKEEDRTFAAFLLHGIKKSADVSFDVCNHDGEC------     322
SLH1_FUb        TYDSLQFGGMTVDQLEHYLHERKTQNRDFVGIALHNIGVSAWVTLSLQVEGGEP------     333
MecrH1_FUb      TYEDLTFGGISLENIEKMIHENQQEDRIYAGFLLAGIRTSANVDIFIKTTDSVQ------     327
SLH1_FUc        HYDNLDFNGMSVAQLQREVIRRRGLERAFAGFMLHGVKKSCLVVFDICKPDGTC------     334
MecrH1_FUc      EYDTLEFNGLSISQLNKKLEAIKSQDRFFAGFLLSGFKKSSLVKFNICTDSSNC------     336
SLH1_FUe        EYDTLELQGLDVQRLQDYINKEKEHDRVFAGFLLSGIGQSAHASFSVCKANGEC------     334
MecrH1_FUe      IYDTLEFAGWSIRGIDHIVRNRQEHSRVFAGFLLEGFGTSATVDFQVCRTAGDC------     335
                 *:   :       :   :       : :. : *  .  :.   . :

SLH1_FUh        VSVGNFYVLGGSSEMPWAYERIYKLDMTEAASKLGLSSE--STF------HFKLTVTKYD     374
MecrH1_FUh      EYAGSFVILGGAKEMPWAYERLYRFDITETVHNLNLTDD---HV------KFRFDLKKYD     375
SLH1_FUa        EFAGDIFILGGANEMAWEFTVPYYYDISDAVRKLGHQQD--FNF------GVKAEVYSVN     390
MecrH1_FUa      EKAGDFFVLGGPSEMKWQFYRPYLFDLSDTVHKMGMKLD--GHY------TVKAELFSVN     403
SLH1_FUd        HPAGKFAILGGEYEMPFTFDRLYKYDISDTVRKLGLRLDSAADF------DVQIKIFAYN     385
MecrH1_FUd      HEAGMFSILGGQTEMSFVFDRLYKLDITKALKKNGVHLQ--GDF------DLEIEITAVN     382
SLH1_FUf        VSAGVIFILGGKLEMPWAFDRLFKLDITDTLHDMGIEPE--DVFDTQAPFFLSYEVHAVN     392
MecrH1_FUf      HHGGQIFVLGGTKEMAWAYNRLFKYDITHALHDAHITPE--DVFHPSEPFFIKVSVTAVN     391
SLH1_FUg        NFAGTFAILGGPLEMPWSFDRLFKYDVTSVFKQMRLRPD--SEY------SFRVSLTAVN     373
OcdoH1          TFGGTFCILGGEHEMFWAFDRLFKYDITTSLKHLRLDAH--DDF------DIKVTIKGID     371
MecrH1_FUg      HFAGTFAILGGEHEMPWSFDRLFRYDITQVLKQMHLEYD--SDF------TFHMRIIDTS     374
SLH1_FUb        YQVGKIAVLGGEKEMPWRFDRLFKVEITSALQKLGLSYD--DDF------NVHLDITDVT     385
MecrH1_FUb      HKAGTFAVLGGSKEMKWGFDRVFKFDITHVLKDLDLTAD--GDF------EVTVDITEVD     379
SLH1_FUc        TKSGEFYLLGDENELPWEYDRLYKYEITHELEDMGLEPQ--DRF------DVQYHVYDLD     386
MecrH1_FUc      HPAGEFYLLGDENEMPWAYDRVFKYDITEKLHDLKLHAE--DHF------YIDYEVFDLK     388
SLH1_FUe        TAAGDFDILGGSAEMPWRFDRLYKYEITDVLEKKGLDVH--DSF------NITVSLTALD     386
MecrH1_FUe      EDAGYFTVLGGEKEMPWAFDRLYKYDITETLDKMNLRHD--EIF------QIEVTITSYD     387
                   * : :**.  *: : :   :  :::     .      .            .   :

SLH1_FUh        GTALNV-TFPDPVIVKRAANSQHDVLVLPLSVANQLPPKIVVRQGTQVVFHASESGV-SS     432
MecrH1_FUh      HTELDASVLPAPIIVRRPNNAVFDIIEIPIGKDVNLPPKVVVKRGTKIMFMSVDEAVTTP     435
SLH1_FUa        GTHLGSDVLSRPYASHRNPRGYTDPVIRDSR-----KP----------------------     423
MecrH1_FUa      GTALPDDLLPHPVVVHHPEKGFTDPPVKHHQ-----SANLLVRKN---------------     443
SLH1_FUd        GSYIDASLLHRPTIIFEPGQGQTQDDVGHVE-----R-----------------------     417
MecrH1_FUd      GSHLDSHVIHSPTILFEAGTDSAHTDDGHTE-----P-----------------------     414
SLH1_FUf        GTTLPLSTISPPTLVFQPAEGAAAEHSSYS------------------------------     422
MecrH1_FUf      GTVLPASILHAPTIIYEPGLDHHEDHHSSS------------------------------     421
SLH1_FUg        GTQLDSRLIEAPSVSFVPGNKPGSKTSAAHE-----DPVPLD------------------     410
OcdoH1          GHVLSNKYLSPPTVFLAPAKTTH-------------------------------------     394
```

```
MecrH1_FUg      GKQLPSDLIKMPTVEHSPGGKHHEKHHEDHH-----------------------------      405
SLH1_FUb        GKKWAEDTFHHNTIIHVPGEGHEDPG----------------------------------      411
MecrH1_FUb      GTKLASSLIPHASVIREHARVKFDKV----------------------------------      405
SLH1_FUc        NTDLGDDVFGKAVIIYSAGQGHQKGHEEDYL-----E-----------------------      418
MecrH1_FUc      PASLGKDLFKQPSVIHEPRIGHHEGEVYQA------------------------------      418
SLH1_FUe        GSALSSSLLPTPSVIFEPKSRTTELH----------------------------------      412
MecrH1_FUe      GTVLDSGLIPTPSIIYDPAHHDISSH----------------------------------      413
                        :

SLH1_FUh        LREVGSYTNSVHCAIPPGQANLYDLDVAYSLEAGDYYFTSSDKTKCQQG-SRIQITVDDE      491
MecrH1_FUh      MLNLGSYTAMFKCKVPPFSFHAFELGKMYSVESGDYFMTASTTELCNDNNLRIHVHVDDE      495
SLH1_FUa        ------------------------------------------------------------      423
MecrH1_FUa      ------------------------------------------------------------      443
SLH1_FUd        ------------------------------------------------------------      417
MecrH1_FUd      ------------------------------------------------------------      414
SLH1_FUf        ------------------------------------------------------------      422
MecrH1_FUf      ------------------------------------------------------------      421
SLH1_FUg        ------------------------------------------------------------      410
OcdoH1          ------------------------------------------------------------      394
MecrH1_FUg      ------------------------------------------------------------      405
SLH1_FUb        ------------------------------------------------------------      411
MecrH1_FUb      ------------------------------------------------------------      405
SLH1_FUc        ------------------------------------------------------------      418
MecrH1_FUc      ------------------------------------------------------------      418
SLH1_FUe        ------------------------------------------------------------      412
MecrH1_FUe      ------------------------------------------------------------      413
```

Table S8. Sequence alignment of selected molluscan hemocyanins. Slipper limpet hemocyanin type 1 (*Crepidula fornicata* ; CrfoH1; GenBank accession number WCA44164.1) is shown in bold text with the key residues participating in the interaction network (FUa only) shown in Figure 6 highlighted in yellow. Key (sequence accession number and organism): Apca; CAD88977.1; *Aplysia californica*. Coas; AYO86684.1; *Cornu aspersum*. EuscH1; AIL00900.1; *Euprymna scolopes*. HaasH1; XP_067664054.1; *Haliotis asinina*. HacrH1; XP_071088435.1; *Haliotis cracherodii*. HadishaH1; AYD59980.1; *Haliotis discus hannai*. HadivH1; ACU00134.1; *Haliotis diversicolor*. HarubH1; ANE23704.1; *Haliotis rubra*. HarufH1; XP_046359783.2; *Haliotis rufescens*. HeluHaD; AEO51767.1; *Helix lucorum*. HepoHaN; AYO86687.1; *Helix pomatia*. LijaH2; XP_064608055.1; *Liolophura japonica*. LisaH1; DAZ89886.1; *Littorina saxatilis*. LystH1; AYO86691.1; *Lymnaea stagnalis*. MecrH1; Q10583.2; *Megathura crenulata*. MetuMH; AGX25261.1; *Melanoides tuberculata*. NunuH1; AH10286.1; *Nucula nucleus*. OcvuHA; CAI9741816.1; *Octopus vulgaris*. PhacH1; WGL47534.1; *Physella acuta*. PocaH1; DAZ89889.1; *Pomacea canaliculata*. RaveH1; DAD54775.1; *Rapana venosa*. SeofH1; ABD47515.1; *Sepia officinalis*. SemaH; AGV74427.1; *Sepiella maindroni*. TopaH1; BAS69907.1; *Todarodes pacificus*. H1; type 1 or type 1-like. H2; type 2 or type 2-like. HA; A-type. MH; mega-hemocyanin. HaD; alphaD. HaN; alphaN.

```
CoasHaN        ---------MSQ--LWF--LISLGLLVWVC-QATLIRKNVDHLSQQDVLNLQKALRDVFD        46
HepoHaN        ---------MSQ--LWF--LISLGLLVWVC-QATLLRKNVDHLSQQDVLNLQKALRDVFD        46
HeluHaD        ---------MAP--TIV--WLA-FTFMLVCSNALLVRKDVDHLTPEEVLNLQKALREVSK        46
ApcaH1         ---------MVG--YLG--QALMALLLLALSNAALVRKSVDQLTSEEILNLQKSLREVND        47
LystH1         ---------MAL--LWS--FLALALFVLSFGDAALVRKNVDNLSEEDIINLQKTLRDVAD        47
PhacH1         ---------MAR--LLP--LLALALYVLSFSEAVLVRKSVDSLSSEDILNLQKSLRAVAD        47
PocaH1         ---------MLD--CRVFLLFWLLHLQVVLTQASLVRKDVATLSSEEILALQKSLLRLEV        49
MetuMH         --------MVTS--PRFALALLAFCVQVAFTTGSLIRKNVENLSSQEILSLQTSLRALAA        50
LisaH1         ---------MTS--SALTLAFVALLAHVALTSGSLRRKNVASLSETEVLSLQTYLRQLED        49
CrfoH1         ---------MTT--FCGPLALLVLWCQLLLASGSLIRKNVDSLTESEVLTLQTVLRQLED        49
RaveH1         ---------MTS--PRVTGTLLFCLAYLCFATGSLLRKNVDTLTEQEILRFQNTLLELEK        49
OcvuHA         MAPTRSPWLLGATILCIISI-----FVPVITNGKFVRKNVDSLTQDEVVSLQVALYSMQK        55
TopaH1         ---------------------------------NLLRKNVDTLTPDEILNLQVSLRAMQD        27
EuscH1         MR-----RLNHLPLLCLAVILTLWMSGSNTVNGNLVRKNVDTLSHEEIISLQVALRSMQD        55
SeofH1         MR-----HLNHLPLLYLTVILTFWMSGLDTVYGKLIRKNVDTLSHDEILNLQIALRNMQD        55
SemaH          MR-----HLNHLPLLYLTVILTFWMTGLDTVYGKLIRKNVDTLSHDEILNLQIALRNMQD        55
LijaH2         ------------------------------------------------------------        0
NunuH1         ---------MRLPLIFLALG-----IQVCIVHPLLVRKDVDSLTTAEVLALQEALKEVQE        46
MecrH1         ----------MLSVRLL-IV-----VLALANAENLVRKSVEHLTQEETLDLQAALRELQM        44
HacrH1         ---------MGFVPLFL-VA-----LVALAGAENVIRKDASHLTADEIQALHGALHAITD        45
HarufH1        ---------MGVVQLFL-VA-----LVALAGAENVIRKDASHLTADEIQALHGALHAVVN        45
HadishaH1      ---------MGVVQLFL-VA-----LVALAGAENVIRKDASHLTADEIQALHGALHAVIG        45
HadivH1        ------------------------------------------------------------        0
HaasH1         ---------MWLVQFLL-VA-----LAAVAGADNVVRKDVSHLTDDEVQALHGALHDLTE        45
HarubH1        ---------MWLVHFLL-VA-----LTTGAGADNVVRKDVSHLTDDEVQALHGALHDLTE        45


CoasHaN        DSSSKGFDAL-AAYHGYPPQCKDG-DRDVACCVHGDLNFPTWHRLYTVQIEQALAEKGL-        103
HepoHaN        DNSSKGFDAI-AAYHGYPPQCQDG-DRAVACCLHGDLNFPAWHRLLTVQIEQALHEKGL-        103
HeluHaD        DTSSKGFAAI-AAYHGYPPKCKHG-SKDVACCVHGEPTFPQWHRLYAVQMEQALKEKGL-        103
ApcaH1         DTSNLGYAAI-ASYHGYPTQCKDG-DRDIACCLHGSPVFPQWHRLYVVQMEQALKAKGL-        104
LystH1         DKSAKGYAAI-AAYHGYPAQCKDANNRPVACCVHGMPVFPQWHRLYVVQLEQALKEKGL-        105
PhacH1         DTSDKGYAAI-AAYHGYPTQCKHG-DKDVACCIHGMAVFPQWHRLYVVQMEQALKEKGL-        104
PocaH1         DKGVTGFQSL-AAIHGEPSQCSNGDNTSVACCVHGMATFPQWHRLYVVQLEQALVGEGLV        108
MetuMH         DESASGFKAV-AAYHGEPSMCEDNDGVAIACCLHGMAVFPQWHRLYVVQFEQLLVNKGL-        108
LisaH1         DKSETGFQSLVAAYHGEPSMCEDAEGHPVACCIHGMATFPQWHRLYTVQFEQALVQRGLS        109
CrfoH1         DKTATGFQAI-AAYHGEPSQCQTKDGTAIACCVHGEATFPQWHRLYVVQFEQALVSKGLA        108
RaveH1         DNSDHGFQAL-GAFHGEPSGCQTKEGSAIACCVHGEATFPMWHRAYTAQFEQLLVEKGLA        108
OcvuHA         DEGPTGFQAI-SAYHGEPADCKAADGSTVVCCLHGMPTFPLWHRLYLVQFEQAMASHGS-        113
TopaH1         DEGASGYQAI-SAYHGEPADCKAADGSTIVCCLHGMPTFPMWHRLYLVQFEQALVAHGS-        85
EuscH1         DDGATGYQAI-SAYHGEPADCKAADGSSIVCCLHGMPTFPLWHRLYIVQFEQALAAHGS-        113
SeofH1         DEGATGYQAI-SAYHGEPADCKAADGSQIVCCLHGMPTFPMWHRLYMVQFEQAVAGHGS-        113
SemaH          DDGATGYQAI-SAYHGEPADCKAADGSTIVCCLHGMPTFPMWHRLYMVQFEQAVAGHGS-        113
LijaH2         ----------------------MEDGTKIACCIHGMPVFPHWHRLYVAQIEQSLVKHGS-        37
NunuH1         DTGPTGYQAI-AAYHGYPGDLCAHDGHKMACCIHGMPTFPQWHRLFTTQMEQALRQKGL-        104
MecrH1         DSSSIGFQKI-AAAHGAPA-SCVHKDTSIACCIHGMPTFPHWHRAYVVHMERALQTKRR-        101
HacrH1         ASGPLSFREI-TSYHASPP-SCDHHGQKIACCVHGMPTFPFWHRAYVVQAERALLSQRK-        102
```

```
HarufH1         ASGPFGFREI-SAYHASPP-SCDHNGRKIACCVHGMPTFPFWHRAYVVQAERALLAQRK-        102
HadishaH1       ASGPLSFREI-SAYHASPP-SCDHNGRKIACCVHGMPTFPFWHRAYVVQAERALLSQRK-        102
HadivH1         ------------------------------------------HRAYVVQAERALLSQRK-        17
HaasH1          ATGPMSFREI-TAYHAAPA-SCDYKGRKIACCVHGMPSFPFWHRAYVVQAERALLSKRK-        102
HarubH1         ATGPMSVREI-TTYHAAPA-SCEYKGRKIACCVHGMPSFPFWHRAYVVQAERALLSQRK-        102
                                                          **   .: *: :  .

CoasHaN         SIGLPYWDWTPQLYKLPDLVSNRVFLDSAGGKTRKNAWYQGNIHVG----AKTYKTARAV        159
HepoHaN         SIGLPYWDWTPQLYKLPDLVSQRVFLDSAGGKTRKNAWYQGYIHVG----KKTYKTARAV        159
HeluHaD         NIGIPYWEWTHQLDHLPELVSQRVFIENDGGKARSNIWYQGQIPTP----EGVKNTARAV        159
ApcaH1          SIGLPYWDWTRPLNHLPELVSQQVFIDSEGGKARGNVWYQGDINID----GQVVHTARAV        160
LystH1          SIGIPYWDWTRPLTKLPALVSQQVFIESDGTKAKKNVWYQGDIEVVENSKTVIRHTARAL        165
PhacH1          SIGIPYWDWTKPLSALPDLVSQQVFIDSDGGKAKKNVWYQGDITVD----GVVKHTARAV        160
PocaH1          GLGVPYWDWTQPLTELPRLVQEPIFKDPQGGLGQRNAWFSGDIEVG----EVHRRTARAV        164
MetuMH          NIGVPYWDTLEPLTHLPSLVEEQIFRDPNGGVGGKNSWFSGEVPVD----GAIKNTARAV        164
LisaH1          NIGVPYWDWTEPLSELPPLVQNQIFRDPNGGIGKMNAWFSGEIDIG----DLHKRTARAI        165
CrfoH1          NIGVPYWDWTKPIKDLPALVKNQIFRDPKGKLGQKNAWFSAEIDHD----NVHTQTARAI        164
RaveH1          NLGVPYWDWTRELTHLPELVRHQIFKDPNGGVGQRNAWFSAEIHHG----DVDEHTARAV        164
OcvuHA          THGVPYWDWTQPMSELPALVQHPLFIDPSGEKAKKNVFYSGAIAFE------KKSTARAV        167
TopaH1          TLGIPYWDWTKPMTQLPELVQHPLFIDPTGKKAKKNVFYSGEIKFE------NKVTARAV        139
EuscH1          ALGVPYWDWTKPMDHLPELVQHPLFIDPNGQKAKKNVFYSGEIKFE------NKVTARAV        167
SeofH1          TLGIPYWDWTKPMSHLPELVQHPLFIDPNGQKAKKNVFYSGEIKFE------NRVTARAV        167
SemaH           TLGIPYWDWTKPMSHLPELVQHPLFIDPNGQKAKKNVFYSGEIKFE------NRVTARAV        167
LijaH2          SIGVPYWDWSKPISDLPDIVREPLFINPAEGKAQKNPFYEGHIDSE------NTYTARAV        91
NunuH1          GIGVPYWDWTQTVSALPTLVRDPIYINSAGGKALKNYWHKGVIKDA-------GTTDRAI        157
MecrH1          TSGLPYWDWTEPITQLPSLAADPVYIDSQGGKAHTNYWYRGNIDFL------DKKTNRAV        155
HacrH1          TVGMPYWDWTETLTQLPALVTEPIFIDSLGGKTQPNYWHRGDIAFA------NKATARAV        156
HarufH1         TVGMPYWDWTETLTQLPALVTEPIFIDSLGGKTQPNYWHRGEIAFA------NKATARAV        156
HadishaH1       TVGMPYWDWTETLTQLPALVTEPIFIDSLGGKTQPNYWHRGEIAFA------NKATARAV        156
HadivH1         TVGMPYWDWTETLTQLPALVTEPIFIDSLGGKTQPNYWHRGEIAFA------NKATARAV        71
HaasH1          TVGMPYWDWTQPLTHLPFLVTEPIYIDSKGGKAQTNFWYRGDIAFI------NKKTARAV        156
HarubH1         TVGMPYWDWTQTLTHLPSIVTEPIYIDSKGGKAQTNYWYRGEIAFI------NKKTARAV        156
                  *:***:    :  ** :. . :: :        * :. . :            * **:

CoasHaN         DKRLYQNVSGDEHTDLFEHVLHAFEYSSYCQFEVQFEVAHSTIHSLVGGRFPYSVSSLDY        219
HepoHaN         DKRLYQNVTGEEHTDLFEQVLHAFEY-GYCQFEVQFEVAHNTIHSLVGGRYPYSVSSLDY        218
HeluHaD         DPRLFQQVETGQYTDLFEHVLNALEYPNYCQFEVQFEVAHNTIHYLVGGRHTYSVSHLEY        219
ApcaH1          DDRLFQQVAPGENTQLFEMVLNALEYENYCQFEVQFEVAHNTIHYLVGGRHKYSMSHLEY        220
LystH1          DDRLFQKVEPGQNTDLFEQVLNALMYPNYCQFEVQFEIAHNTIHYLVGGRNKYSMSHLEY        225
PhacH1          DDRLFQKVDAGEHTDLFEHVIDALQYPNYCQFEVQFEVAHNTIHYLVGGRNKYSMSHLEY        220
PocaH1          DERLFQTVAPGQNSRLLDTVLHALEFDDYCQFEVHLEVAHNNIHYLVGGRHEFSMSTLEW        224
MetuMH          DDRLFEKVAPGKNTQLFNIILDALEQDDYCQFEVQLEVAHNHIHYLVGGRHQYSMATLEY        224
LisaH1          DDRLFERVAPGGKSQLYNLILDALEQDDYCQFEVQFEVAHNHIHYLVGGRHRYSMSTLEY        225
CrfoH1          DDRLYESVSPGGHTTLFNMVLDALEQDDYCQFEAQFEVAHNHIHYLVGGRHHYSMSTLEY        224
RaveH1          DERLFEHPQDGGKTRLFNMILDALEQDDYCQFEAQFEVAHNHIHYLVGGRHHYSMSTLEY        224
OcvuHA          DDHLFDAS-HGKKNSLLENILMAFEQEEYCRFEVQFEVAHNPVHFLVGGRYTYSMSSLEY        226
TopaH1          DARLYQASQEGQKNFLLEGVLNALEQEDFCHFEVQLEVAHNPIHYLVGGRFTHSMSSLEY        199
EuscH1          DARLYEASQKGAKNFLLEGVLNALEQEDYCHFEVQFEVAHNPIHYLVGGRFTHSMSSLEY        227
SeofH1          DARLYEASKEGHKNFLLEGVLNALEHEDYCHFEVQLEVAHNPIHYLVGGRFTHSMSSLEY        227
SemaH           DARLYEASKDSHKNFLLEGVLNALEHEDYCHFEVQLEVAHNPIHYLVGGRFTHSMSSLEY        227
LijaH2          DERLFEKPDAGKFTPLMEGILLALEQEDFCDFEVQFEVAHNAIHYMVGGPYNFGMSTLDY        151
NunuH1          DDRLFEQPETGHYTNAMEKVLLALEQEDYCKFEIQFEVAHNDIHYLVGGRYGKSMSSLEY        217
MecrH1          DDRLFEKVKPGQHTHLMESVLDALEQDEFCKFEIQFELAHNAIHYLVGGKHDYSMANLEY        215
HacrH1          DDRLFEKVETGHYTKLMESVLDALEQDEFCKFEIQFELAHNAIHYLVGGKYEYSMSNLDY        216
HarufH1         DDRLFEKVETGHYTKLMESVLDALEQDEFCKFEIQFELAHNAIHYLVGGKYEYSMSNLDY        216
HadishaH1       DDRLFEKVETGHYTKLMESVLDALEQDEFCKFEIQFELAHNAIHYLVGGKYEYSMSNLDY        216
HadivH1         DDRLFEKVETGHYTKLMESVLDALERDEFCKFEIQFELAHNAIHYLVGGKYEYSMSNLDY        131
HaasH1          DDRLFERVEPGQYTHLMETVLDALEQDEFCKFEIQFELAHNAIHYLVGGKFEFSMSNLEY        216
HarubH1         DDRLFEKVEPGHYTHLMETVLDALEQDEFCKFEIQFELAHNAIHYLVGGNFEYSMSNLEY        216
                * :*::       .   : :: *:    :* ** ::*:**. :* :***    .:: *::

CoasHaN         TAYDPLFYLHHSNVDRLFAIYQEVQRIRGIESGSA---NSPICDVKTFYRPLEPFSRTSN        276
HepoHaN         TGYDPLFYLHHSNVDRLYAIYQEVQKLRGVQSGSA---NSPLCDVKSYYRPLEPFRRKSN        275
HeluHaD         TSYDPIFFLHHSNVDKIYTIYETIQRSRGYTPGPGCTGECELCDIVGFRTPLEPLNRDSN        279
ApcaH1          TSYDPIFFLHHSNVDRILAVKLAL-----LGPGAPQKGGVEFCDLKNSMTPMAPFSWDSN        275
LystH1          TSYDPIFFLHHSNVDKIYSIYEALQKEKGYTPGPGCTGKCELCDLKHFDEPLAPFSWETN        285
PhacH1          TAYDPIFFLHHSNVDRIYAIYEALQQERGFIPGPGCKGKCELCDVKLFKQPMEPFSRDSN        280
PocaH1          TSYDPLFFLHHSNVDRIFAVWQELQRRRGKTY------DHADCSVELFRKQLEPFNRDSN        278
MetuMH          SSYDPIFFLHHSNIDRIFVIWQELQKKRGKPY------DHSDCSVEQFRWNLKPFDRDTN        278
LisaH1          SAYDPIFFLHHSNVDRIFAIWQQLQKKRGKPY------DHADCSVELFNQNLHPFDRDTN        279
CrfoH1          TSFDPLFFLHHSNVDRIFAIWQELQKKRGKPY------DHADCSVELFNKDLLPFARDSN        278
RaveH1          SSYDPIFFLHHSNVDRLFVIWQEMQKRRGKPY------DHADCSVELFNKPMEPFKRDSN        278
OcvuHA          TSYDPLFFLHHSNVERLFTIWQALQKHRGHDS-------KINCGLNLLHTPMEPFGRDSN        279
TopaH1          TSYDPLFFLHHSNVERHFALWQALQKHRGLPT-------RPNCGLNLFHSPMEPFGRDTN        252
EuscH1          TSYDPLFFLHHSNVERQFALWQALQKHRGLPT-------RPNCGLNLFHNPMEPFGRDTN        280
SeofH1          TSYDPLFFLHHSNVERQFALWQALQKHRGLST-------RPNCGLNLFHSPMEPFGRDSN        280
SemaH           TSYDPLFFLHHSNVERQFALWQALQKHRGLST-------RPNRGLNLFHSPMEPFGRDSN        280
```

```
LijaH2          TSYDPIFFLHHSMVDRLFAIWQALQKQRNKPS------DHSNCAIQDMKRSMRPFNFDTN       205
NunuH1          TSYDPIFFLHHSNVDRIFAIWQALQKKRGLPW------DHSNCGLEMFRTPLEPFGRDSN       271
MecrH1          TAYDPIFFLHHSNVDRIFAIWQRLQELRNKDP------KAMDCAQELLHQKMEPFSWEDN       269
HacrH1          TSYDPIFFLHHSNVDRIFAIWQRLQELRGKNP------KAIDCAHELAHQELEPFNRATN       270
HarufH1         TAYDPIFFLHHSNVDRIFAIWQRLQELRGKNP------NAIDCAHELAHQELEPFNRATN       270
HadishaH1       TAYDPIFFLHHSNVDRIFAIWQRLQELRGKNP------KAIDCAHELARQELEPFNRDTN       270
HadivH1         TAYDPIFFLHHSNVDRIFAIWQRLQELRGKNP------KAIDCAHELARQELEPFNRDTN       185
HaasH1          TSYDPIFYLHHSNVDRIFAIWQRLQELRGKNP------KAMDCAHDLARQQLEPFNRDSN       270
HarubH1         TSYDPIFFLHHSNVDRIFAIWQRLQELRGKNP------NAMDCAHDLSRQQLQPFNRDSN       270
                :.:**:*:**** :::   :   :                           : *:    *

CoasHaN         PFSLTKTYNSPDKAKDYSVFGYEYESLELNGLSPAQIVELVKQRQSHDRAFAAFSLHNIG       336
HepoHaN         PFSLTRTYNSADKAKDYSVFGYEYESLELNGLSPAQIVDLVKKRQSHDRAFAVFSLHNIG       335
HeluHaD         PFALTRVHSHPYEATEHTLFGYKYDNLTLNGLDVDKIKSIIEKRQEADRAFASFRLHGIG       339
ApcaH1          PIALTRDHSLPPQTLAHSTFGYSYDDLSLNGIPGGEQSTS-EGETNVRPRFCQLPSLRIG       334
LystH1          PFAVTKQFNLPREAVDGSIYGYKYDNLTLNGYDLNGIKRLLKEKWSHAASYAVFRLSGIS       345
PhacH1          PFAITKDHSVPKETIDFSTFGYKYEDLSLNGYDLAGIKRLIKEKWSHDHVYATFRLHGIE       340
PocaH1          PITLTKTFSQAKDLFSYDQLGYKYDNLTLGGLSMDKLFSLVEQRRARERAFAAFTLKGLG       338
MetuMH          PIALTKQFHTAKELFSNLQLGYVYDDLTLNGMDVDQLYNLMEQRRARERAFGVFSLHGLG       338
LisaH1          PIQLTRTFSQAKDLFSHLQLGYTYDDMSLNGLSLDQLYSLLEQRRARERAFAVFSLHGLG       339
CrfoH1          PIQLTRTFSKPKDLFSYMQLGYTYDDLTLNDMSIDQLYSFLKERHARERSFAVFSLHGMG       338
RaveH1          PVELTRTFSKPRDLFSHLQLGYTYDDLELGGMSLEELHAYLQQRHGRDRTFATFALHGLG       338
OcvuHA          PIELTKNNAKAADIFTADHLEYNYDDLTLNGMSISELDSLLKERQSHPRSFANFRLGGIK       339
TopaH1          PFAITKDNSKASSLFDYEHLGYAFDDLSLNGMTIEELEALLKQRRSGARAFANFRLGGIK       312
EuscH1          PFAITKDNAKPSSLFEYEHLGYAYDDLTLNGMSVEELDSFLKSRKNQARAFANFRLGGIK       340
SeofH1          PFAITKDNAKPSSVFDYEHLGYEYDDLTLNGMSIGELDTLLKVRKSQARAYASFRLGGIK       340
SemaH           PIAITKDNAKASSVFDYEHLGYEYDDLTLNGMSIEELDTLLKVRKSQARAYASFRLGGIK       340
LijaH2          PYQLTQSNAAPDNVWNFNVLDYDYDTLELNGMNIEQLNKLLSDRKAEARVFAAFRLSGIG       265
NunuH1          PFEVTKSHSKGADAFDYQNLGYQYHDLTLNGMSVDDLYTLLNERKTHERAFASFRLYGFG       331
MecrH1          DIPLTNEHSTPADLFDYCELHYDYDTLNLNGMTPEELKTYLDERSSRARAFASFRLKGFG       329
HacrH1          PVPITKQHSRPVDLFDYHQLGYSYDSLNLNGMTPEELKIKLDERHSKERAFASFRLKGFG       330
HarufH1         PVPITKQHSRPVDLFDYHQLGYSYDSLNLNGMTPEELKIKLDERHSKERAFASFRLKGFG       330
HadishaH1       PVPITKQHSRPVDLFDYHQLGYSYDSLNLNGMTPEELKIKLDERHSKERAFASFRLKGFG       330
HadivH1         PVPITKQHSRPVDLFDYHQLGYSYDSLNLNGMTPEELKIKLDERHSKERAFASFRLKGFG       245
HaasH1          PVQLTKDHSRPVDLFDYKQLGYSYDSLFLNGMSPEKLKTELDERRSKERAFASFRLSGFG       330
HarubH1         PVQLTKDHSRPVDLFDYKQLGYSYDSLYLNGMSPEKLKSELDERHSKERAFASFRLSGFG       330
                   :*.      .        * :. : *..          . .      :  :    :

CoasHaN         VSADIKVRVCLDIDDEHE---------EDDRC-LHAGDFFVLGGATEMDWTFPRPFFFEI       386
HepoHaN         VSADVKVRVCLDIDDEHE---------EDDRC-LHAGDFFVLGGATEMDWAFPRPFFFEI       385
HeluHaD         VSADVRVKVCVSDSRH-L---------AGDYC-EFAGNFFILGGPLEMPWTFNRPYYCEI       388
ApcaH1          SSANVRVEVCVPSSDK-L---------TGDYC-EFAGDFFILGGAIEMAWAFIWPYYFEI       383
LystH1          TSANVRVQVCVPSEDE-V---------TGTYC-EFAGDFFVLGGPLEMPWAFSRPYYFEV       394
PhacH1          TSANVRVEVCIPAKDA-I---------SEPNC-EFAGDFFVLGGPTEMPWAFSRPYYFDV       389
PocaH1          FSANVRVKVCQRDATG-----------SDGHC-EFAGDIFLLGGANEMAWEFNLPYYFDV       386
MetuMH          FSANARVKVCSDLTGRRKRTIGSADLDVEHHC-EFAGDFFLLGGANEMPWEFHLPFYFDV       397
LisaH1          FSANVKVEVCEGSDEDH-------SDHSRHHC-EFAGDFFILGGSNEMPWEFHQPYYFDV       391
CrfoH1          FSANVKVSVCNVEEE------------ETTNC-EFAGDIFILGGANEMAWEFTVPYYYDI       385
RaveH1          FSANVRVKVCDVHNDGER-QGDAASQAEEDHC-EFSGDFFILGGANEMPWEFTVPYYFDV       396
OcvuHA          TSANVHISVCIPSKDSR----------KSDDCHHDAGEFFILGGKREMPWDFGYPYLHDI       389
TopaH1          TSANVRIKLCIPTEDKR----------QSDNCDNDAGQFFILGGTNEMPWNFAFPYLHEI       362
EuscH1          TSANVRINICIPNKDGR----------HSDNCDNYAGGFFILGGVHEMPWNFAYPYLHEI       390
SeofH1          TSANVRINICIPSKDGR----------HSDNCDNYAGEFFILGGIHEMPWDFSYPYLHEI       390
SemaH           TSANVRINICIPSKDGR----------HSDNCDNYAGEFFILGGIHEMPWDFSYPYLHEI       390
LijaH2          TSADVKMYVCIPSGDAK----------REDDCSHYAGRFFILGGPIEMPWSFDRHYLQDI       315
NunuH1          GSANVRVQVCNRDDDEH----------SPDRC-HFAGDVSVLGGPTEMPWAFHQPYLFDI       380
MecrH1          GSANVFVYVCIPDDNDR----------NDDHC-EKAGDFFVLGGPSEMKWQFYRPYLFDL       378
HacrH1          GSANVVVYACLPDSDPR----------SDDHC-EKAGDFFVLGGSTEMPWIFYRPYFFDV       379
HarufH1         GSANVVVYACLPDSDPR----------SDDHC-EKAGDFFVLGGSTEMPWIFYRPYFFDV       379
HadishaH1       GSANVVVYACLPDSDPR----------SDDHC-EKAGDFFVLGGSTEMPWVFYRPYFFDV       379
HadivH1         GSANVVVYACLPDSDPR----------SDDHC-EKAGDFFVLGGSTEAPWVFYRPYFFDV       294
HaasH1          GSANVVVFACIPDDDPR----------SDDYC-EKAGDFFILGGQSEMPWKFYRPFYFDI       379
HarubH1         GSANVVVYACIPGDDPR----------SDDYC-EKAGDFFILGGASEMPWKFYRPFFFDV       379
                 **:  :  *                     *   :* . :***  *  * *   :  ::

CoasHaN         THTVEQLHLPLQGNYHVEADIVAVNGTQLPSSLL-VHPYVSFRPAAGSHDEPIDHNLPGH       445
HepoHaN         THTVERLHLPLDGNYHVEADIVSVNGTQLPSSIL-PHPYVSFRPAAGSHDKPIEHNEPEH       444
HeluHaD         TKTVEHMNISLTSDYHVEVDVYSVNGTQLPSDIL-PHPSVSFRPGVGKRDPEIVQK----       443
ApcaH1          TKTVQNLGVALDGNYNVQARIFSVNGTELPSNLI-PSPSVSFRPAVGKTDPPMAHGESEH       442
LystH1          TKTVEKLGLALDGNYRIEAELFSVNGTQLPSSLL-PHPFVSYRPAKGTVDTPIAHNEVNH       453
PhacH1          TKTVEKLGVPLDSNYRIKAQLFSVNGTQLSSSLL-PHPYVSYRPAKGRTDPAIAHNEVNH       448
PocaH1          SDAVVRLGIPLDGDFLVKAELYSVNGTLLSSGLL-PPPIGAHRPARGYRDPVPAMY----       441
MetuMH          SDAVQRLGLSLTSKWSLEWDLYHVNGTALPGNLL-PTPYGKHRPAKGDRDSEEVSFFAD-       455
LisaH1          TDAVEQLGLPLDGRYHVSAEVFSVNGTSLGSGDL-PHPYGSHRPPHGHTDPTPG------       444
CrfoH1          SDAVRKLGHQQDFNFGVKAEVYSVNGTHLGSDVL-SRPYASHRNPRGYTDPVIR------       438
RaveH1          TEAVHKLKLGPGGHYGVKADVYSVNGTLLGAHVLGSRPYLSHRPAEGYHDPEIH------       450
OcvuHA          TDTVKSLGLELDGNYYLKAEVTAINGSHLPSDII-PKPIVTYIPEVGHHDPEMIKV----       444
```

```
TopaH1          TDTVLSLGLALDSNYYVTAEVTAINGTLMPTQTI-PRPIVTYIPPQGFKDVNMVNM----        417
EuscH1          TDKVISLGLPLSGDYYVQAVVTAINGTLLPDGII-PSPIVSYVPGHGHLDDEMKNV----        445
SeofH1          TDTVNSLGLPLSGNYYVQAIVTAINGTLLPDGII-PNPIVSYVPSVGLKDEEMKVV----        445
SemaH           TDTVTSLGLPLSGNYYVQAIVTAINGTSLPDGIL-PNPIVSYVPSAGLKDEEMKVV----        445
LijaH2          TKAVDDANLPYDGNYYVSIKVSAVNGTSIPSSVL-YKPQVVYQPAKQFVDVTPPKG----        370
NunuH1          TDTFHKHGIDFHQNFYIKTELYAVNGSALPSDAL-VSGTGVHQPPAGYLDPKPAPV----        435
MecrH1          SDTVHKMGMKLDGHYTVKAELFSVNGTALPDDLL-PHPVVVHHPEKGFTDPPVKHH----        433
HacrH1          TKAVQRLGVVLSGHYYVKTELFSVNGTSLSSDIL-PQPTVAYRPGKGHIDPPIHHR----        434
HarufH1         TKAVQRLGVVLSGHYYVKTELFSVNGTSLSPDIL-PQPTVAYRPGKGHIDPPVHHR----        434
HadishaH1       TKAVQRLGVALSGHYYVKTELFSVNGTSLSPDIL-PQPTVAYRPGKGHIDPPVHHR----        434
HadivH1         TKAVQRLGVALSGHYYVKTELFSVNGTSLSPDIL-PQPTVAYRPGKGHIDPPVHHR----        349
HaasH1          TKAVKRLGVPLSGHYYVKTNLFSVNGTFLSPDLL-PQPTVAYRPGKGHRDPPVHHR----        434
HarubH1         TDAVHRLGVPLSGHYYVKANPFSVNGTSLSPDLV-PQPTVAYRPGKGHLDPPVHHR----        434
                :. .          : :      :**: :    :       .       *

CoasHaN         EHEFHENVVVRKNVDRLTIEEVVQLREALQEFQNDKSVEGYQAIAEFHGDPGKCPNPTAK        505
HepoHaN         EHEFHENVAVRKNVDRLTREEVVQLREALQEFLNDKSVEGYQAIAEFHGDPGKCPDPTAK        504
HeluHaD         -LEHKEEVSIRKDVDHLTREEILELREALEKLQSDHSVDGYQAIAEFHGDPGKCPFPTAR        502
ApcaH1          GHEVHDGVSIRKDIDTLTREEVNDLRQALTKFQNDASVDGYQATAEFHGDPGKCPYPNAK        502
LystH1          EHEYHEGISVRKDIDRLTNEEIYELREALQKFQNDKSVDGYQAIAEFHGDPGKCPNPNSK        513
PhacH1          EHEYHDDVAVRKDVDRLTREEVYELRQALARFQNDSSVDGYQAIAEFHGDPGKCPTPTSK        508
PocaH1          ---RTDATVVRKNVKALTDEETLALRQALSRFQNDTSVDGFQAVAEFHGLPARCPHPDAR        498
MetuMH          -RDVHRHAVVRKNVVDLTDEEVYELRLAMDRFQNDTSVDGYQAIAEFHGLPARCPRPDAA        514
LisaH1          -DGKGPTTVVRKDIAVLTDEEVYALRQAMARFQNDTSVDGFQAVAEFHGLPAKCPHPDAA        503
CrfoH1          -DSRKPSNVVRKDIELLTDEEVYALRQAMHRFQNDTSIDGYQAVAEFHGLPAKCPHPDAA        497
RaveH1          -PDLKKTTLVRKDVETLTDEEVYYLRQAMAKFQNDTSIDGFQAVAEFHGLPPKCPHPDAA        509
OcvuHA          ---DKADVKIRKDVSVLTKEEEYGVRIAMERFMLDKSINGYQALAEFHGLPAKCPRPDAP        501
TopaH1          ---DTSSLRFRKDISSLTTEEEYELRVAMERFMSDKSINGYQALAEFHGLPAKCPRPDAL        474
EuscH1          ---DEMKLSTRKDLSSLTDEEEYELRQAMERFMSDKSINGYQALAEFHGLPAKCPRPDAQ        502
SeofH1          ---DVTKLKTRKDVSSLTDEEKYDLRVAMERFMSDKSINGYQALAEFHGLPAKCPRPDAL        502
SemaH           ---DATKLKTRKDVSSLTDEEKYDLRVAMERFMSDKSINGYQALAEFHGLPAKCPRPDAL        502
LijaH2          ---DPAALAVRKDVSRLTPTEVYNLREAMSRFAKDKSVAGFEATAEYHGLPARCPRPDAK        427
NunuH1          ---DRTGLAVRKNLDRLTDEEVDSLRKAMARLQSDKSVDGYDAIAEYHGLPARCPRPDAA        492
MecrH1          ---QSANLLVRKNINDLTREEVLNLREAFHKFQEDRSVDGYQATAEYHGLPARCPRPDAK        490
HacrH1          ---QNEDYIVRKNINHLTRSETYELRKALERFQADTSVDGFQATVEYHGLPARCPEPDAK        491
HarufH1         ---QNEDYIVRKNINHLTRSETYELRKAMERFQADTSVDGFQATVEYHGLPARCPEPDAK        491
HadishaH1       ---QNEDYIVRKNINHLTRSETYELRKAMERFQADTSVDGFQATVEYHGLPARCPEPDAK        491
HadivH1         ---QNEDYIVRKNIDHLTRSETYELRKAMERFQADTSVDGFQATVEYHGLPARCPEPDAK        406
HaasH1          ---HDEDLIVRKNIDHLTREEEYELRRALERFQADTSVDGYQATVEYHGLPARCPQPDAK        491
HarubH1         ---HDDDLIVRKNIDHLTREEEYALRRALERFQADTSVDGYQATVEYHGLPARCPQPDAK        491
                          **::  **  *   :* *: .:  * *: *::* .*:** * :** * :

CoasHaN         DRHACCIHGLPTFPHWHRLIVTQVEDSLRRRGAPLGLPYWDFTRPDTHVPQLANDETYFN        565
HepoHaN         DRHACCIHGLPTFPHWHRLIVTQVEDSLRRRGAPLGVPYWDFTRPDTHVPLLASEEIYFN        564
HeluHaD         DRFACCIHGMPNFPHWHRLLVVQVEDALRRRGAHTGIPYWDWTKPNTHIPALAADETYVN        562
ApcaH1          NRLACCIHGMPTFPHWHRLFVVQVEDALRRRGAQIGIPYWDWTKPGTQIPALAAEETYVD        562
LystH1          NRKACCIHGMPTFPHWHRLLVVQVEDALRRRGSNIGVPYWDWTKPRTSVPKLAADETYTD        573
PhacH1          VRQACCIHGMPTFPHWHRLLVVQVEDALRRRGSHIGIPYWDWTKPGTSIPALAADHDYLD        568
PocaH1          VRYACCLHGVPTFPHWHRLFVVQVEDALRTRGLDIGIPYWDWTGMDTTVPPLADDDVYLD        558
MetuMH          VRYACCIHGMATFPHWHRLFVVQIEDELKARDLHFGMPYWDWTVPNAQIPAIAAEATYQD        574
LisaH1          LRYACCIHGMATFPHWHRLFVVEVEDELRSRGLEFGIPYWDWTKPNAAIPALAADITYTD        563
CrfoH1          VRYACCVHGMATFPHWHRLFVVEVEDELRSRGLEIGIPYWDWTRPNAHIPALANDVSYED        557
RaveH1          VRYACCIHGMATFPHWHRLFVTEVEDELKSRGLEFGIPYWDWTRPNTHVPALAAEETYED        569
OcvuHA          NRIACCEHGMATFPHWHRLVIMQLEDALADRGSTVGVPYWDWSKPITHLPSLVAEETYQD        561
TopaH1          NRVACCIHGMATFPHWHRLVVMQFENALFTRGSPIGVPYWDWTKPFTALPSLLADETYVD        534
EuscH1          NRVACCVHGMATFPHWHRLVVVQFENALMARGSQIGVPYWDWTKPITALPHLLGDETYVD        562
SeofH1          NRVACCVHGMATFPHWHRLVVVQFEDALINRGSPIGVPYWDWTKPMKALPDLLADETYVD        562
SemaH           NRAACCVHGMATFPHWHRLVVVQFEAALINRGSPIGVPYWDWTKPMKALPDLLAQETYVD        562
LijaH2          ERFACCVHGMATFVHWHRLYVVQVEDAIRRRGSGVGVPYWDWTEPIEALPALAGEATYLD        487
NunuH1          ERVACCVHGMPTFPHWHRLYMVQLEDALVDRGSTIGLPYWDWTKPLIGLPDLISNKMYKD        552
MecrH1          DRYACCVHGMPIFPHWHRLFVTQVEDALVGRGATIGIPYWDWTEPMTHIPGLAGNKTYVD        550
HacrH1          VRFACCMHGMASFPHWHRLFVTQVEDALVRRGSPIGVPYWDWTKPMTHLPDLAASANYVD        551
HarufH1         VRFACCMHGMASFPHWHRLFVTQVEDALVRRGSPIGVPYWDWTKPMTHLPDLAASENYVD        551
HadishaH1       VRFACCMHGMASFPHWHRLFVTQVEDALVRRGSPIGVPYWDWTKPMTHLPDLAASEDYVD        551
HadivH1         VRFACCMHGMASFPHWHRLFVTQVEDALVRRGPPIGVPYWDWTKPMTHLPDLAASEDYVD        466
HaasH1          VRFACCMHGMASFPHWHRLFVTQVEDALVRRGSPIGVPYWDWTRPMTHLPELASNETYID        551
HarubH1         VRFACCMHGMASFPHWHRLFVTQVEDALVRRGSPIGVPYWDWTKPMTHLPELASDATYID        551
                 * *** **:  * ***** : :.*  :  *.   *:****::     :* :  .  * :

CoasHaN         PHI-GQNVHNPFHDVEIAFLGNDVRTERDI-SESLAQTPAWGEHTELFNAFLLALEQDNF        623
HepoHaN         PHT-GGNLHNPFHDVQIAFLGNDVHTERDI-SPSLAQTPAWGEHTELFNAFLLALEQDYF        622
HeluHaD         PHD-NAEHVNPFHHAVIGFLGGDAKTSRDP-LPELTQTPKWGDHTELFDVFLLALEQDNF        620
ApcaH1          PEN-GETVRNPFFGADVAFLNSGAKTSRDV-SDSLNDVPEWGDHTNLFDQYLYALEQEDF        620
LystH1          PTT-NEVKRNPFHDAPIAFLGDSTKTTRDV-SSSLSDSPKWGDHTELFDAFLLALEQDNF        631
PhacH1          PKG-RVPSQPLPSRPHLSFLGADAKTGRDV-DNSLSQQPKLGDHTELFDAFLLALEQDNF        626
PocaH1          PIT-GEFQHNPFHDAPVAFIGE--RTSRQV-QAELTQTPKFGDHTDLFDGMLLAFEQTDF        614
MetuMH          PHHPDVTLHNPFYDAAVAFLNT--RTTRDV-QNDLAENPIYGDHSKLYDGILLAFEQTDF        631
```

```
LisaH1         PHS-GVEVHNPFHDAAVAFLGE--RTTRDTMVLELSKNAEFGDHTPLFDGILLAFEQTDF        620
CrfoH1         ANT-HQQLHNPFHDAPIAFLGE--KTTRQV-QDDLSESPEFGDHTSLFDGILLAFEQTDF        613
RaveH1         PHT-HHQVHNPFHDALVAFLKK--KTTRDV-QADLTETPAFGDHTALFDGMLLAFEQTDF        625
OcvuHA         PFT-LESKPNPFFRASIDFLHSGVYTTRDV-DPRLLMQPAKGDHCPLYDGLLLAYEQDEY        619
TopaH1         PYT-KETKPNPFFKAPIEFLKAGVHTSRQI-DERLFKQPSKGDHGFLYDGLSLAFEQDDF        592
EuscH1         PYT-KETKANPFFSSAIEFLDAGVHTTRVI-DPRLFKQPSVGDHSAMYDGMLLAFEQEDF        620
SeofH1         PYT-KETKPNPFFSAPIEFMHAGVSTKRVI-DARLFKEPTVGDHSYLHDGLMLAFEQHDF        620
SemaH          PYT-KETKPNPFFSAPIEFMHAGISTKRVI-DARLFKEPTVGDHSYLHDGLMLAYEQHDF        620
LijaH2         TKT-NERLPNPFFKGQIPMVG--SETSRDV-QDGLFEKPAFGEHTSLDEEVMYALEQDHF        543
NunuH1         PQT-GANTDNPFHHGSVAFEG--HVTDRDP-DSRLFEDPSYGDHTALFDGMLLALEQEDF        608
MecrH1         SH--GASHTNPFHSSVIAFEENAPHTKRQI-DQRLFKPATFGHHTDLFNQILYAFEQEDY        607
HacrH1         PN--GHTRHNPFFNANISFEEGHHHTSRSI-DARLFAPAAYGDHTPLFDGILYAFEQEDF        608
HarufH1        PN--GHTRHNPFFNANISFEEGHHHTSRSI-DARLFAPAAYGDHTALFDGILYAFEQEDF        608
HadishaH1      PN--GHTRHNPFFNANISFEEGHHHTSRSI-DARLFAPAAYGDHTALFDGILYAFEQEDF        608
HadivH1        PN--GHTRHNPFFNANISFEEEHHHTSRSI-DARLFAPAAYGDHTALFDGILYAFEQEDF        523
HaasH1         PY--GHIHHNPFFNANISFEEGHHHTSRSL-DPRLFAPAAFGDHTHLFDGILYAFEQEDF        608
HarubH1        PH--GHIHHNPFYNANISFAEGHHHTSRSI-DSRLFAPAAYGDHTHLFDGILYAFEQEDF        608
                               : :      * *      *      *.*  : :    * **  :

CoasHaN        CKFAVQFEVAHNLIHGLVGGNTSHGLSTLSYSAFDPIFYIYHSNIDRIWAIWTALQQLRG        683
HepoHaN        CKFAIQFEVAHNLIHGLVGGNTPHGLSTLSYSAFDPIFYIYHSNIDRIWAIWTALQEHRG        682
HeluHaD        CDFEVQFEIAHNLIHAYVGGNSKYGLSSLSYSAFDPIFYLHHSNIDRIWAIWTALQQHRG        680
ApcaH1         CNFEVQFEVAHNLIHALVGGNTQFGLSSLSYSAFDPIFFLHHSNVDRIWAIWTALQQYRG        680
LystH1         CDFEVQFEVAHNLIHALVGGKEQYSLATLSYSAFDPIFYLHHSNVDRIWAIWTALQELRG        691
PhacH1         CDFEIQFEVAHNLIHALVGGKNPHSLATLSYSAFDPIFYLHHSNVDRIWAIWTALQELRG        686
PocaH1         CDFEIQFEVVHNAPHFLVGGFAPYSLATLHYSAFDPIFYLHHSSVDRMWAIWQALQVLRG        674
MetuMH         CDFEIQFEVIHNSPHFLVGGFGAYTLATLHYSAFDPIFYLHHSNVDRMWAIWQELQIRRG        691
LisaH1         CDFEVQFEVTHNAPHFLVGGFAPYSLTTLHYSAFDPIFYLHHSNVDRLWAIWQKLQIIRG        680
CrfoH1         CDFEVQFEVVHNAPHFLVGGFGAYTLATLHYSAFDPIFYLHHSNVDRLWAIWQQLQIKRG        673
RaveH1         CDFEVQFEVVHNAIHFLVGGFDPYTMATLHYSAYDPIFYLHHSNVDRLWAIWQKLQMRRG        685
OcvuHA         CDFEVQFEVTHNFVHAWIGGKEAYSMSSLHYTSFDPLFWLHHAQVDRLWAIWQALQIHRR        679
TopaH1         CDFEVQFEVTHNSIHAWTGGSEPYSMSSLHYTSFDPMFWLHHSQVDRLWAIWQALQIQRG        652
EuscH1         CDFEVQFEVTHNAIHAWTGGAEPYSMSSLHYTSFDPMFWFHHSEVDRLWAIWQALQVHRK        680
SeofH1         CDFEVQFEVTHNAIHRWTGGSEPYSMSSLHYTSFHPMFWLHHSEVDRLWSIWQSLQIHRG        680
SemaH          CDFEVQFEVTHNAIHAWTGGSEPYSMSSLHYTSFDPMFWLHHSEVDRLWSIWQSLQIHRG        680
LijaH2         CDFAVQFEIAHNRIHALVGGSQRYSMSSLHFTSYDPLFFLHHSNVDRLWAIWQQLQILRG        603
NunuH1         CDFEVQFEVTHNNLHAWVGGSAKYSMSSLHYTAFDPVFYLHHSNVDRLWAMWQALQQYRG        668
MecrH1         CDFEVQFEITHNTIHAWTGGSEHFSMSSLHYTAFDPLFYFHHSNVDRLWAVWQALQMRRH        667
HacrH1         CDFEIQFELVHNSIHAWIGGSEDYSMSTLHYTAFDPIFYLHHSNVDRLWAIWQALQIRRQ        668
HarufH1        CDFEIQFELVHNSIHAWIGGSEDYSMSTLHYTAFDPIFYLHHSNVDRLWAIWQALQIRRQ        668
HadishaH1      CDFEIQFELVHNSIHAWIGGSEDYSMSTLHYTAFDPIFYLHHSNVDRLWAIWQALQIRRQ        668
HadivH1        CDFEIQFELVHNSIHAWIGGSEDYSMSTLHYAAFDPIFYLHHSNVDRLWAIWQALQIRRQ        583
HaasH1         CDFEIQFELVHNSIHAWVGGAEDYSMATLHYTAFDPIFYLHHSNVDRLWAIWQALQIRRH        668
HarubH1        CDFEIQFELVHNSIHAWIGGSEDYSMATLHYTAFDPIFYLHHSNVDRLWAIWQALQIRRH        668
               *.* :***: **  *   **   . :::* ::::.*:*:::*:.:**:*::*  **  *

CoasHaN        KPYKAHCAQSYVHTPLKPFAFKPPYNNDAKTFAHSTATNIYDYEKELAYTFDSLEFGGMS        743
HepoHaN        KPYKAHCAQSYVHTPLKPFAFKPPYNNDAKTYAHSTATNIYDYEKELAYTYDSLEFGGMS        742
HeluHaD        KPYKAHCAQSYVYTPLKPFAFHTPYNNNEKTFAHSTPTDVYDYEKEFQYGYDNLQFGGLG        740
ApcaH1         KPYKAHCAQSYVNQPLKPFAFSSPLNNNEKTFSHSVPTNVYDYAEELGYDYDNLQFGGMG        740
LystH1         KPYKAHCAQSYTYEPLKPFAFSSPYNNDQKTFTHAVPRNVYDYETELNYAYDSLEFGGLT        751
PhacH1         KPYKAHCAQSYTYEPLKPFAFASPYNNNEKTSSHAVPRNVYDYETELKYTYDQLEFGGMS        746
PocaH1         LPYQAHCALSLTRDTLKPFGFPSPLNNNQKTFSRARGDQVYEYQKNFLYDYDSLTFGGMT        734
MetuMH         LPYRAHCAHTMTHEPMKPFSFAAPLNNNEKTFSHSEPSQVYDYERDLGYTYDSLTFGGMT        751
LisaH1         LPYKAHCASTQTQEVLKPFGFPPPWNNDEKTFNNARPTNVYDYENVLDYTYDTLQFGGMT        740
CrfoH1         LPYKAHCASSLTQEVLKPFGFPPPWNNDHKTFENARPTNIYDYEAVLDYTYDSLQFGGMT        733
RaveH1         KLYKAHCAGSLTQELMMPFGFPPPYHNDPKTHDNARPSQIYDYESVLDYTYDSLQFGGMT        745
OcvuHA         KPYKAYCANSEVHRPLKPFAFESPFNNNEHTRAHAIPTDVYDYQANLGYTFDSLSFGGMS        739
TopaH1         KPYKTYCANSEVYRPMKPFAFKSPLNNDEKTREHSVPTDVYDYQAELAYTYDTLFFGGLS        712
EuscH1         LPYKCHCASSEVYRPMKPFAFEPPLNNDHQTHSHSVPTHIYDYQAELKYAYDTLFFGGMS        740
SeofH1         LPYKAHCASSEVYQPMKPFAFDAPLNNDELTHSHSVPTDIYDYQSELEYAFDTLFFGGMS        740
SemaH          LPYKAHCASSEVYQPMKPFAFDAPLNNDELTHSHSVPTNIYDYQSELDYAFDTLFFGGMS        740
LijaH2         KPFKAYCANSDVHMSLKPFGFPAPLNSNPKTFEHAVPTNIYDYENELDYTYDTLQYHGMT        663
NunuH1         KPYKAHCATTYTYYPLKPFAFKSPLNNNEKTQKHAVPTNVYDYESELGYSFDSLEFGGMG        728
MecrH1         KPYRAHCAISLEHMHLKPFAFSSPLNNNEKTHANAMPNKIYDYENVLHYTYEDLTFGGIS        727
HacrH1         KPYRAHCAQSIEQLPMKPFAFPSPLNNNEKTHSNSVPTDIYDYEETLHYSYDDLTFGGMN        728
HarufH1        KPYRAHCAQSIEQLPMKPFAFPSPLNNNEKTHSNSVPTDIYDYEETLHYSYDDLTFGGMN        728
HadishaH1      KPYRAHCAQSIEQLPMKPFAFPSPLNNNEKTHSNSVPTDIYDYEETLHYSYDDLTFGGMN        728
HadivH1        KPYRAHCAQSIEQLPMKPFAFPSPLNNNEKTHSNSVPTDIYDYEETLHYSYDDLTFGGMN        643
HaasH1         KPYQAHCAQSVEQMPMKPFAFPSPLNNNEKTHSHSVPTDIYDYEEVLHYSYDDLTFGGMN        728
HarubH1        KPYQAHCAQSVEQTPMKPFAFSSPLNNNEKTHSHSIPTDIYDYEEELHYSYDDLTFGGMN        728
                 :: :** :     : **.*  * :.:  *  .:   .:*:*   : * :: * : *:

CoasHaN        VPELDNYINTNIKNKNRVFVGIQLHGIQTSGLAHIYIEAPGKD-KFEAGRFAILGGPTEM        802
HepoHaN        VPELDNYINTNIKNKNRIFVGIQLHGIKTSGLAHIFIEAPGKE-KLEAGRFAILGGPTEM        801
HeluHaD        IPQLENYINEHLKSKSRTFVGIHLHGIKTSGLATIHVSASGK--DYVAGHFAILGGPSEM        798
ApcaH1         IAELDQYIKSTQKSKDRVFYGILLHGIKKAALATVYVKKPGVDTEYNAGQFALLGGPSEM        800
```

```
LystH1           IKQLNDYINNERKTKNRVFAGVLLWGIKRSALATLYVN------DVEAGRFALLGGPSEM    805
PhacH1           VQQLNDYINNNQKTKDRTFAGILLHGIKKSATARIYVTAPGKD-KYEAGRFVVLGGPSEM    805
PocaH1           IPQLNQFLA-ERGNRDRTFVGVKLHNIGVSAWVKLYISV-PGGERYLANSFAVLGGEREM    792
MetuMH           IEQLENYLE-ERQSKERVFVGIELHNIGASAWVKVLIDSGSGGEPYLVGKIAVLGGEKEM    810
LisaH1           VDQLEHYLE-ERQTRDRAFVGIALHNIGVSAWVDVKINA-PGGESYQVGHVAVLGGEKEM    798
CrfoH1           VDQLEHYLH-ERKTQNRDFVGIALHNIGVSAWVTLSLQV-EGGEPYQVGKIAVLGGEKEM    791
RaveH1           IAQLDHYLE-ERKTHDRTFVGIMLHNIGISAWATLAIEM-KNGEEYTVGKVAVLGGEKEM    803
OcvuHA           IRELQKHID-EKKTHDRVFAGFLFGGIKTSANIDLYVVA--AGNEYLASPISILGGTKEM    796
TopaH1           IRELQRYVE-EAKSKDRVFAGFLLMGIQTSANVDLFVVA--GGNEFFVGSIAVLGGSKEM    769
EuscH1           VRELQGHID-NNKHKDRVFAGFLFMGIKTSAIVDFYVVA--AGNEFMAGSVAVLGGSKEM    797
SeofH1           IRELQEHIQ-ENELKDRVFAGFLFMGIKTSANIDFYVVA--GENEFMAGSIAVLGGSKEM    797
SemaH            IRELQEHIE-ENELKDRVFAGFLFMGIKTSANIDFYVVA--GENEFMAGSIAVLGGSKEM    797
LijaH2           MEQLNIYLR-RRGQKDRVFAGCNLKGIRTSAMVDFQICKQDGTCGESAGHFAVLGGPFEM    722
NunuH1           IQELDHYIQ-ERKATDRVFVGFLLEGFQKSANVDFFIER-SGQDKFKAGTIAVLGGSAEM    786
MecrH1           LENIEKMIH-ENQQEDRIYAGFLLAGIRTSANVDIFIKT-TDSVQHKAGTFAVLGGSKEM    785
HacrH1           LDEIEETIH-HRQQHERVFAGFLLGGIGKSALVNIFINK-PGSSPHKAGDIAILGGTKEM    786
HarufH1          LDEIEDTIH-HRQQHERVFAGFLLGGIGKSALVNIFINK-PGSSPHKAGDIAILGGTKEM    786
HadishaH1        LDEIEETIH-HRQQHERVFAGFLLGGIGKSALVNIFINK-PGSSPHKAGDIAILGGTKEM    786
HadivH1          LDEIEETIH-HRQQHERVFAGSLFGGIGKSALVNIFINK-PGSSPHKAGDIAILGGTKEM    701
HaasH1           LDQIEKAIH-LRQQHERVFAGFLLAGIGKSALVDIFIKK-PGSEPFKAGDFAILGGSKEM    786
HarubH1          LDEIEEAIH-LRQQHERVFAGFFLGGIGKSALVDIFIKK-TGSEPHKAGDFAILGGAKEM    786
                 : :::  :       .* : *  : .:  :.   . :          .. . :***  **

CoasHaN          PWRYDRLYKHEITNALETLQLHWAQPYNVNIEIARFDGTPIDAHQFGHVEVLYLPGKQTK    862
HepoHaN          PWRYDNLYKHEITNALEKLDLHWAQPYNVTIEIAQFDGTPIDAKQFGHLEILYLPGKQTK    861
HeluHaD          DWDYDRPYRHDISHALEELGVNWAQPFDVTIEMHTFDNKPIDVSQFPRINIIHKEQEQAH    858
ApcaH1           EWRFDRNYRFEITKALEELGINFLSPFDVRLEMNEFDGTPIDVSQFPKSQLIFKRRDILP    860
LystH1           EWRFDRLYRHEITDTLEELNLHWAQPYTVTLKLTDFEGHDIPINPFPKPQIIFKAKEEPK    865
PhacH1           QWRFDRLYRQEITNALNELNLQWNDDYTVSVEYEAIDNTPISDSELTHIQIIYKAKEDVK    865
PocaH1           TWTFDRLFKVEITDTLLAIGRNYDEDFDVTLDITDVQQQKLSVDTFSHTTIFHQPKKDYD    852
MetuMH           PWHFDRLFKMEITDALSDLGLSYDDNFDVTLQITDVTGKEWSSDSFQHDTIFHEPGKTFE    870
LisaH1           AWHFDRLFKLEITDALSALGLRYDDDFEVHLHIVDVTGKQWPEDTFSHSTILHSPAQVHE    858
CrfoH1           PWRFDRLFKVEITSALQKLGLSYDDDFNVHLDITDVTGKKWAEDTFHHNTIIHVPGEGHE    851
RaveH1           PWHFDRTFKVEITEALHKLGYRYDDEYDVKLHIKDVTGKEWPEDTFSHHTIIHVPASTHL    863
OcvuHA           PWRFDRLFRQEITDSLKALGVDPFGDFTLRVDIKDVNGTALPSTIISQPIVIFEPGKVET    856
TopaH1           TWRFDRVYKHEITDALGALGVDMFAEYTLRVDIKDVNGTALPPTAIPPPIVIFVPGIADA    829
EuscH1           SWRFDRVYKHEITGALAALGVDKYAEYTLRVDIKDVNGTALPATTLPQPTVIFVPGKADT    857
SeofH1           SWRFDRVYKYEITAALAALGVDKYAEYTLRVDIKDVNGTALSANILPSPTVIFTPGKVE-    856
SemaH            SWRFDRVYKYEITAALAALGVDKYAEYTLRVDIKDVNGTALSANILPSPTVIFTPGKVE-    856
LijaH2           DWRYDRPYKHEITKALSALGLTKEDVFSIHVDITDVNGTALNSNLVPSPSIIFSPAAGEE    782
NunuH1           KWRFDRVYKHEITESLTTLGLTKDSDFSISVTLTDVDGTELDASVLHDPTIIFEPSHAAE    846
MecrH1           KWGFDRVFKFDITHVLKDLDLTADGDFEVTVDITEVDGTKLASSLIPHASVIREHARVKF    845
HacrH1           PWAFDRLFKVEITDALKALSVDVDGDYEVTFDIHDMHGNKLSSDLIPHPAVVSEPAHPSF    846
HarufH1          PWAFDRLFKVEITDALKALALDVDGDYEVTFDIHDMHGNKLSSDLIPHPAVISEPAHPTF    846
HadishaH1        PWAFDRLYKVEITDALKALALDVDGDYEVTFDIHDMHGNKLSSDLIPHPAVISEPAHPSF    846
HadivH1          PWAFDRLYKVEITDALKALALDVDGDYEVTFDIHDMHGNKLSSDLIPHPAVISEPAHPSF    761
HaasH1           PWAFDRLYKVEITDALKNLSLEVDGDYEITINIHDMHGKALDADLIPHAAIISEPAHPSF    846
HarubH1          PWAFDRVYKVEITDALKILSLEVDGDYEVTFTIHDMHGNELDTDLIPHAAVISEPAHPSF    846
                  * :*. :: :*:  *  :       : : .    .         .    :.

CoasHaN          KSA---DVEVHVRKEVSQLTGEEILDLRHALANLEVDKSIGGYQTLGRYHGASKWCPSPS    919
HepoHaN          KPA---DVEVHVRKEVSQLTEEEILDLRHALSNLEEDKSIGGYQTLGRYHGAYKWCPSPS    918
HeluHaD          DTS---PITKITRKNVNHLTEEEILNLRHSLAFLEEDRSVGGYQTLGRFHGTPNWCPYPS    915
ApcaH1           EAQ---IDEVTVRKNVDSLNAEEVLALRRALANLKEDSSIGGYQTLGRYHGTPLWCPAPD    917
LystH1           DVS---ADTIKIRKNVATLTEAEVVDLRQALANLQNDQSAGGYQDLGRFHGTPNWCPTPD    922
PhacH1           DET---PDSHRVRKNVNSLTREEVSALREAMLNLQGDNSAGGYQDLGRFHGTPKWCPSPD    922
PocaH1           GDA--PEEVGRMRYNVDSLTREQAQSLRDALQKLQNDSTVNGFGHIAAFHGQPSWCPAPD    910
MetuMH           VRGV---SDKRTRLDVDHLNSEQIQNLRDAFKALEDDTSINGFNQIAAFHGQPSWCPSPD    927
LisaH1           HEGMTFGDREYLRSDVTSLSREQIQNLRDAMQSLESDTSINGYNQIAAFHGQPNWCPSPD    918
CrfoH1           D-P--GEDDDHVRNDIVTLTAEQEQNVRDAFQGLKNDKSVAGYNQIAAFHGQPNWCPSTQ    908
RaveH1           EED--HKEEDHVRNDVDTLTKEQIQNMREALATLKQDHSPGGFDHLAAFHGQPNWCPSMQ    921
OcvuHA           DVK--FDENFRTRKEVSTLTASQMAALREALEKYEADKTANGYQQVAAFHGSTEWCPSPD    914
TopaH1           NVK--FDEQHRSRKNVDSMTVSEMNALRTAMAAFAADKEVTGYQQVAAFHGSTQWCPSPD    887
EuscH1           DVE--FDEQHRSRKNVDSMTKTEMDSIRSALTAFAADKSANGFQQVAAFHGSTKWCPSPD    915
SeofH1           DVH--FDEQHRSRKNVDSMTKSEMEDLRKAMTAFAADKGVTGYQQVAAFHGSTKWCPSPD    914
SemaH            EVH--FDEQHRSRKNVGSMTKSEMDDLRKAMTTFAADKGVTGYQRVAAFHGSTKWCPSPD    914
LijaH2           E-F--ADDWTIVRKDVTRLSPEETTALRVALQAVKDDSSVGGFQTIAGYHGIPTLCPTPD    839
NunuH1           D-R--PTDFRVVRKNADRLTETETDSLVHALKKLQEDTGAGGFAQMGAFHGLPNWCQVG-    902
MecrH1           D-K--VPRSRLIRKNVDRLSPEEMNELRKALALLKEDKSAGGFQQLGAFHGEPKWCPSPE    902
HacrH1           E-D--VSTSLRIRKNVDYLTPEETNDLRHALDLLEKDPSAGGFNQLGAFHGEPKWCPHPE    903
HarufH1          E-D--VSTSLRIRKNADYLTPEETNDLRHALDLLEKDPSPGGFNQLGAFHGEPKWCPHPE    903
HadishaH1        E-D--VTTSLRIRKNVDYLTPEETNDLRHALDLLEKDPSAGGFNQLGAFHGEPKWCPHPE    903
HadivH1          E-D--VTTSLRIRKNVDYLTPEETNDLRHALDLLEKDPSAGGFDQLGAFHGEPKWCPNPE    818
HaasH1           E-D--ETHSFRIRKNVDSLTPEETNELRKAMDLLQKDHTAGGFNQLGAFHGEPKWCPNPE    903
HarubH1          E-D--EAHSLRIRKNVDSLTPEETNELRKALDLLQKDHSAGGFNQLGAFHGEPKWCPNPE    903
                             * :   :.  :   :  ::     *    *:  :. :**    *
```

```
CoasHaN         AEVKKVCCPHGMAIFPHWHRLLVVQFENALTNHGYHGAVPYWDWTQPLDSLPALVTAETY    979
HepoHaN         AEVKKVCCPHGMSIFPHWHRLLVVQFENALSSHGYHGAVPYWDWTLPLDSLPALVTAQTY    978
HeluHaD         AEKKVACCPHGGPTFPHWHRLLTVQAENALRKHGYKGALPYWDWTQPATSLPAIVVPETY    975
ApcaH1          AEKKVACCLHGMPVFPHWHRLLTVQAENALRRHGFSGGLPYWDWTRPMTQLPDLVDSAKY    977
LystH1          AEVKKSCCLHGMPTFPHWHRLWTVQAENALRRHGYVGAIPYWDWTQPVKSLPDLVTSEKY    982
PhacH1          AEVKKSCCLHGMPTFPHWHRLWTVQAENALRRSGLKGAIPYWDWTQPIAALPELVQSETY    982
PocaH1          APVKYACCVHGMAVFPHWHRLLTVQMENALRNKGFKEGLPYWDWTQPMSSLPDFVEEESY    970
MetuMH          AETKYACCLHGMPTFPHWHRLITVQLENALRDKGFESGLPYWDWTLPMTELPESFTAETY    987
LisaH1          AEKKYACCAHGMAIFPHWHRLLTVQAENALHANGLQDGLPYWDWTLPMETLPEIVRDETY    978
CrfoH1          AERKYACCTHGMAVFPHWHRLLTVQAENGLIANGLHSGLPYWDWTLPMTSLPSIVLNATY    968
RaveH1          AEHKVACCPHGMPVFPHWHRLLTVQAENALIAHGMHSGLPYWDWTLPMTALPKFVADATY    981
OcvuHA          AEHKYACCHHGMATFPHWHRLLTLNFENGLRRNGYDGGIPYWDWLNPISALPNLVAEEQY    974
TopaH1          AAQKYACCHHGMATFPHWHRLIALNFENGLRRNGWSGGLPYWDWTRPIDALPALVLEAEY    947
EuscH1          AAQKYACCHHGMATFPHWHRLVTLNYENGLRRNGYSGGVPYWDWTRPIDALPALVLEEQY    975
SeofH1          AAQKYACCHHGMATFPHWHRLLTLNFENGLRRNGYSGGIPYWDWTRPIEALPALVLEEQY    974
SemaH           AAQKYACCHHGMATFPHWHRLLTLNYENGLRRNGYSGGIPYWDWTRPIEALPALVLEEQY    974
LijaH2          AASKFSCCVHGMATFAHWHRLYTVQFEQALRKQGFTGALPYWDWSKPISSLPALATDDTF    899
NunuH1          -DEKVACCAHGMAVFPHWHRLLVVQFEAGLHKYGYKGGVPYWDWSSPMDSLPTLANAATY    961
MecrH1          ASKKFACCVHGMSVFPHWHRLLTVQSENALRRHGYDGALPYWDWTSPLNHLPELADHEKY    962
HacrH1          AEHKVACCVHGMAVFPHWHRLLALQAENALRRHGYSGALPYWDWTRPLKKLPDLVTPETY    963
HarufH1         AYHKVACCVHGMAVFPHWHRLLALQAENALRRHGYSGALPYWDWTRPINKLPDLVTPETY    963
HadishaH1       AEHKVACCVHGMAVFPHWHRLLALQAENALRRHGYSGALPYWDWTCPINKLPDLVTPETY    963
HadivH1         AEHKVACCVHGMAVFPHWHRLLALQAENALRRHGYSGAFPYWDWTHPISKLPDLVTPETY    878
HaasH1          AVHKVACCVHGMAVFPHWHRLLALQAENALRKHGYTGALPYWDWTRPLSHLPNLVTPEQF    963
HarubH1         AEHKVACCVHGMAVFPHWHRLLALQAENALRKHGYSGALPYWDWTRPVSKLPDLVAHEHF    963
                   *  ** **   * ***** .:: * .*   *   ..*****  *   **       :

CoasHaN         TDPGSNKDVANPFYSAHI--DDANQDTERSPK-PELFLKPKYGDYTAIAKQVLLAFEQED    1036
HepoHaN         PDPASNKDVANPFYSAHI--DDVNQDTERSPR-PELFQKPKYGEYTSIAKQVLLAFEQED    1035
HeluHaD         IDPSNNQETHNPFHDAYI--DDVNQNTVRSVR-SDLYQQPAFGEYTDIAKQVLYALEQDN    1032
ApcaH1          SDPKSGQELDNPFYSGHI--DDANADTVRSVR-DDLFQQPPFGGYTDIAKQVLLALEQDD    1034
LystH1          RDPSSNNEIKNPFYSAHI--TDADQDTVRSPR-DDLFEQPKFGQNTAIAKQVLLALEQDN    1039
PhacH1          EDGN-GKSVKNPFYSAHI--TDVDKDTTRSVR-DDLFQKPAFGEYTDVAKQVLLALEQED    1038
PocaH1          QNPRTEDIEPNPFHHGTV--EG--QDTTRSPR-EDLFKVPEYGQMTPVAEQVLLALEQTD    1025
MetuMH          DNPVSGANEANPLHHAVV--EG--HKTTRSVR-GELFQQPAFGKFTRIGEQVMLAFEQTD    1042
LisaH1          IHPKTGQSTENPLHHAVV--DG--HKTVRTLREEELFEAPAFGKLTRIAEKVMLAFEQTD    1034
CrfoH1          VHPKTGESVANPLYSGQV--DG--HDTTRSVR-QELFEQPKFGHMTKIAEKVMLAFEQDN    1023
RaveH1          ENPKTHHTEPNPWFSGEV--DG--HNTSRMVR-DDLFEQPEFDKMTRIAEKVMLAFEQDN    1036
OcvuHA          TDM-NGETHVNPFFSGSI--DHIGKTTSRAPS-ADLFLQPKFGEFTSLTNEVIYALEQED    1030
TopaH1          TDA-NGEAKPNPFFSGAI--DSIGASTSRAPT-EALYEKPDFGKYTHLANEIISALEQED    1003
EuscH1          TDV-NGEAHPNPFFSGAI--DEAGAVTSRAPS-ANLFEKPEFGKYTHLANEIIFALEQED    1031
SeofH1          TDS-NGESHPNPFFSGAI--DEAGAVTSRAPS-EHLYEKPDFGKYTELANEVIYALEKED    1030
SemaH           TDS-NGESHPNPFFSGAI--DEAGAVTSRAPS-EHLYEKPDFGKYTELANEVIYALEKED    1030
LijaH2          KDPTTGEDKPNPWLSATIAA-AGDVATERAPR-DELFVQPGFGQFTNLGKQLLYAFEQED    957
NunuH1          THPEDGSSIPNPFYSFDVVEGGVSHTTSRAPR-QELFENPGFGKHTKVAKQVMLAFEQED    1020
MecrH1          VDPEDGVEKHNPWFDGHI--DTVDKTTTRSVQ-NKLFEQPEFGHYTSIAKQVLLALEQDN    1019
HacrH1          TDPSDHQDKHNPWHNGHI--DTVDEYTTRNVR-EDLYQQPEFGHFTDIAQQVLLALEQDD    1020
HarufH1         TDPSDNQDKHNPWYNGHI--ETVDQYTTRNVR-EDLYQQPEFGHFTDIAQQVLLALEQDD    1020
HadishaH1       TDPSDNQDKHNPWYNGHI--ETVDQDTTRNVR-EDLYQQPEFGHFTDIAQQVLLALEQDD    1020
HadivH1         TDPSDNQDKHNPWYNGHI--ETVDQDTTRNVR-GDLYQQPEFGHSTDIAPQVLLALEQDD    935
HaasH1          EDPSDHHVKHNPWYNGHI--DSVNQTTTRSVR-ADLYQKPEFGHYTDIAQQVLLALEQDD    1020
HarubH1         TDPSDHHEKHNPWFNGHI--DTVNHNTTRSVR-SDLYQQPEFGHFTDIAQQVLLALEQDD    1020
                 .        **     :        * *      *:  * :.  * :  ::: *:*: :

CoasHaN         FCDFEVQFEIAHNFIHTLVGGSKPYSMASLAYTAYDPLFFLHHSNTDRLWAIWQALQKNR    1096
HepoHaN         FCDFEVQFEIAHNFIHTLVGGSKPYSMASLAYTAYDPIFYLHHSNTDRLWAIWQALQKNR    1095
HeluHaD         YCDFEVQYEIAHNFIHALVGGSEVYSMASLLYTAFDPLFYLHHSNTDRIWAIWQALQAYR    1092
ApcaH1          FCTFEVQYEIAHNFIHALVGGSEQFSLASLQYTAFDPIFFLHHSNTDRIWAIWQALQKYR    1094
LystH1          FCDFEVQFEIAHNFIHALVGGNEQFSMASLLYTAFDPLFYLHHANTDRLWAIWQALQKYR    1099
PhacH1          FCTFEVQFEIAHNFIHALVGGQEPYSLASLMYTAFDPLFYLHHSNTDRIWAIWQALQKYR    1098
PocaH1          FCSFEVQFEIAHNYIHALVGDKEEYSMASLRYTAYDPLFYLHHSNTDRLWAMWQVLQGYR    1085
MetuMH          YCSFEVQFEVAHNYIHALVGGNELYSMASLRYTAYDPLFALHHSNTDRIWAIWQALQKYR    1102
LisaH1          FCDFEIQFEIAHNYIHALVGGNEEYSMASLRYTAYDPIFYLHHSNTDRIWAIWQQLQEYR    1094
CrfoH1          FCDFEIQYEIAHNYIHALVGGNETYSMASLRYTAYDPIFFLHHSNTDRIWAIWQALQKYR    1083
RaveH1          FCDFEIQYEIAHNHIHALVGGNKLYSMASLRYTAFDPLFFLHHSNTDRIWAIWQTLQKMR    1096
OcvuHA          FCSFEVQFEIAHNHIHALVGGTEPYSMSSLEFTAYDPIFLLHHSNVDRIWAIWQALQKHR    1090
TopaH1          FCDFEVQYEIAHNHIHALVGGTEAVSMASLEYSAFDPIFMLHHSNVDRIWATWQALQKFR    1063
EuscH1          FCDFEVQYEIAHNHIHALVGGTEPFSMASLEYSAFDPIFMLHHSNVDRIWSTWQALQKFR    1091
SeofH1          FCDFDVQFEIAHNHIHALVGGTEAFSMSSLEYSAFDPIFMLHHSNVDRIWATWQALQKFR    1090
SemaH           FCDFGVQFEIAHNHIHALVGGTEAFSMSSLEYSAFDPIFMLHHSNVDRIWATWQALQKFR    1090
LijaH2          FCDFSVQFEITHNAIHALVGGNKPFSMASLRYTTFDPIFFLHHSSVDRYWAIWQALQKFR    1017
NunuH1          FCSFETQFEIAHNFIHAYVGGKDEYSMASLRYTAFDPIFYLHHSNTDRIWAMWQALQKYR    1080
MecrH1          FCDFEIQYEIAHNYIHALVGGAQPYGMASLRYTAFDPLFYLHHSNTDRIWAIWQALQKYR    1079
HacrH1          FCSFEVQYEISHNFIHALVGGTTPYGMASLRYTAYDPIFFLHHSNTDRIWAIWQALQKYR    1080
HarufH1         FCSFEVQYEISHNFIHALVGGTTPYGMASLRYTAYDPIFFLHHSNTDRIWAIWQALQKYR    1080
HadishaH1       FCSFEVQYEISHNFIHALVGGTTPYGMASLRYTAYDPIFFLHHSNTDRIWAIWQALQKHR    1080
HadivH1         FCSFEVQYEISHNFIHALVGGTTPYGMASLRYTAYDPIFFLHHSNTDRIWAIWQALQKYR    995
```

```
HaasH1        FCSFEVQYEISHNFIHALVGGKDPYGMASLRYTAYDPIFFLHHSNTDRIWAIWQSLQKYR        1080
HarubH1       FCSFEVQYEISHNFIHALVGGTDPYGMASLRYTAYDPLFFLHHSNTDRIWAIWQSLQKYR        1080
              :* *  *:*::** **: **.    .::** ::::**:* ***:..** *: ** **  *

CoasHaN       GKSHNSANCATGLLRKPLAPFSLDSDVNPDPVTKAHSQPHQVFDYKQSFHYEYDNLEFNG        1156
HepoHaN       GKPHNSANCATGLLRKPLAPFSLDSDVNPDPVTKAHSQPYQVFDYKDSFHYDYDNLEFNG        1155
HeluHaD       GKPENAANCAIGKLRIPLPPFSLTPDVNPDPVTREHSSPLRVFNYKDSFQYEYDTLDFSG        1152
ApcaH1        GKPYNTANCAIGQLRNPLSPFSLTSDINPDPVTREHSVPFQVFDYQTNFHYEYDALEFNG        1154
LystH1        NKPYNTANCAIGQLRKPLSPFSLTSSVNPDANTREHSLPFQVFDYRNNFHYEYDNLEFNG        1159
PhacH1        GKPYNTANCAIGQLRKPLSPFSLTSDINPDADTREHAVPFQVFDYRNNFHYEYDNLEFNG        1158
PocaH1        GLPMFTANCALTSFRQPLQPFAQPSVVNPDPITRENSIPFRVFDYTRNFHYRYDNIDFNG        1145
MetuMH        GLPYNTANCAITQLRKPLQPFAQTSTVNPDSVTRDHSVPFDVFDYSSSFHYDYDNLEFNG        1162
LisaH1        GLPYNSANCAIASLRKPLQPFAQSSVVNPDPVTRDHAVPFDVFNYEDSFHYHYDNFQFNG        1154
CrfoH1        GKPYNSANCAIAEMRKPLQPFAQTSLTNPDYVTRDHSTPFDVFNYHSSFHYHYDNLDFNG        1143
RaveH1        GKPYNSANCAIAELRKPLQPFAQTSVTNPDPVTRDHSVPFDVFKYHENFHYRFDNMQFNG        1156
OcvuHA        GNAYNSANCAIELLRQPMSPFSLSSDINVDSQTREHSVPFDAFDYTKSFHYEYDSLDVSG        1150
TopaH1        GKAYNSANCAIEILRKPMSPFSLASDINPDAMTREYSVPFDVFNYKKNFHYEYDTLELNG        1123
EuscH1        GKPYNTANCAIEMLRKPMSPFSLASDINPDSMTREHSVPFDVFDYKKNFHYEYDTLELNG        1151
SeofH1        GKPYNTANCAIELLRKPMSPFSLASDINADAMTREHSVPFDVFDYKKAFHYEFDTLELNG        1150
SemaH         GKPYNTANCAIELLRKPMSPFSLASDINADAMTREHSVPFDVFDYKKAFHYEFDTLELNG        1150
LijaH2        GKPYNKANCALEQMRGSLMPFNLGADVNPNDLTRDNSAPFRVFDYENVFGYNYDNLEFNG        1077
NunuH1        GKPYNRANCALDSMRAHLKPFAMPSSVNPYPITRDNSIPFGAFDYKSNFHYSYDNLEFNG        1140
MecrH1        GKPYNVANCAVTSMREPLQPFGLSANINTDHVTKEHSVPFNVFDYKTNFNYEYDTLEFNG        1139
HacrH1        GKPYNTANCAIASMRKPLQPFGLSSDVNPDIITREHAIPFDVFNYKDNFHYKYDTLEFNG        1140
HarufH1       GKPYNTANCAIASMRKPLQPFGLSSDVNPDIITREHAIPFDVFDYKDNFHYKYDTLEFNG        1140
HadishaH1     GKPYNTANCATASMRKPLQPFGLSSDVNPDIITREHAIPFDVFNYRDNFHYKYDTLEFNG        1140
HadivH1       GKPYNTANCAIASMRKPLQPFGLSSDVNPDIITREHAIPFDVFNYRGNFHYKYDTLEFNG        1055
HaasH1        GKPYNTANCAIASMRRPLQPFGLGDDINPDSITREHAIPFDVFNYKDNFHYGYDSLEFNG        1140
HarubH1       GKPYNTANCAIASMRRPLEPFGLSSDINPDIITREHAIPFDVFNYKDNFHYVYDTLEFNG        1140
              .     ****   :*  : **      *     *:  : *  .*.*   * * :* ::..*

CoasHaN       LTIPQLERLIQHNKGEDRVFAGFLLHGIQHSALLRFNICKTEDHCEDEGGEFYILGDPHE        1216
HepoHaN       LTIPQLERVIQHNKAEDRVFAGFLLHGIEHSALLRFNICKTEDHCEPQGGEFYILGDPHE        1215
HeluHaD       LGIPQLAKLLEENKADDRVFAGFLLHGVGHSALVTFFICRNDTDCHNHGGEFYILGDPNE        1212
ApcaH1        LSVPQLARVLEQNKAGDRVFAGFMLHGIQQSALVKFYICKSDDDCNNYAEEFYVLGDVNE        1214
LystH1        LSIPQLAKELEKNKGDDRVFVGFMLHGIKRSALIKFQIHKNDTEFKE-AGEFYILGDENE        1218
PhacH1        LSIPQLAKQLEEIKGKDRVFAGFMLHGLKQSALVTFSICKSENSCKK-AGEFYILGDINE        1217
PocaH1        LSIPQLQREIVKRQSRERVFVGFMLHGIQRSALVVLNLCQV--TCQK-AGEFYILGDANE        1202
MetuMH        LSIAQLQHEVVVRKAHERVFAGFMLHGIKQSALVRFEICSSGGACGQ-AGEFYILGDDYE        1221
LisaH1        LSIPQIQREVVRRQAKERIFAGFMLHGVEQSVLVVFEICKPNGDCKE-AGEFYLLGDEYE        1213
CrfoH1        MSVAQLQREVIRRRGLERAFAGFMLHGVKKSCLVVFDICKPDGTCTK-SGEFYLLGDENE        1202
RaveH1        MSPPQLLRETVRRKGLDRVFAGFMLHGIQKSALVVFEICKPDGTCKE-AGEFYLLGDEFE        1215
OcvuHA        MSIPQLQTEINKRRAKDRIFVTFLLEGHKQSLLVEYYIRLHGSTDRNKAGEFYVLGSENE        1210
TopaH1        LSIXQLSREINRRKAKNRVXVTFMLEGLKKSLLVEYFIAADGTDQKMKAGEFYVLGSENE        1183
EuscH1        LSIPQLSREINRRKAKNRVLVTFMLEGLKKSVLVEYFIKDDGSDNKMKAGEFYVLGSENE        1211
SeofH1        MSIPQLSREINRRKSKNRVFITFMLEGLKKSLLVEYYIKDDGSDNKMKAGEFYVLGSENE        1210
SemaH         MSIPQLSREINRRKSKNRVFITFMLEGLKKSLLVEYYIKDDGSDNKMKAGEFYVLGSENE        1210
LijaH2        LSISDLSHEIENQKSHDRVFAQFMLHGIEKSALLKFYICLSAEDCDHFAGEFYILGDVAE        1137
NunuH1        LSIPQLSRELESRKSLYRVFAGFMLHGIKDTVLVKFYICLSNGDCSNYAGEFLFLGDPAE        1200
MecrH1        LSISQLNKKLEAIKSQDRFFAGFLLSGFKKSSLVKFNICTDSSNCH-PAGEFYLLGDENE        1198
HacrH1        LSISQLNRELDRIKSNDRVFAGFLLSGIKKSVLVKFNVCAPPHDCH-PAGEFYLLGDENE        1199
HarufH1       LSISQLNRELDRIKSHDRVFAGFLLSGIKKSVLVKFNVCAPPHDCH-PAGEFYLLGDENE        1199
HadishaH1     LSISQLNRELDKIKSHDRVFAGFLLSGIKKSVLVKFNVCAPPHDCH-PAGEFYLLGDENE        1199
HadivH1       LSISQLNRELDKIKSHDRVFAGFLLSGIKKSVLVKFNVCAPPHDCH-PAGEFYLLGDENE        1114
HaasH1        LSISQLNRELEKIKSHDRVFAGFLLSGIKQSALVKFEVCTPPDQCH-KAGEFYLLGDENE        1199
HarubH1       LSISQLNRELDKIKSHERVFAGFLLSGIKKSALVKFEVCTPPDHCH-KAGEFYLLGDENE        1199
              :   ::        :.  *    *:* *   : *:   :           . ** .**.  *

CoasHaN       IPWSYNRLFKYEITEQLKSLGLHYDDSYTIHYTLLDVDGADLG--QRYSQPTVIHEVGTS        1274
HepoHaN       IPWSYNRLFKYEITEQLKSLGLHYDDNYNIHYTLLDLDGTDLG--QRYSQPTVIHQVGTS        1273
HeluHaD       MEWSYDRLYKYEITEELKKLHLRYNDRYFVRYEIHDLTGQDLG--QPFPTPTVIRQIGTS        1270
ApcaH1        MAWSYDRLYKYEITDALAALGLRYNDRYSVRYEVLNLQGEDIG--QPFPTPTVVKEDGTS        1272
LystH1        MEWSYDRLYKYEVTQQLEDFGLRYNDRYYIKYQVLDLDGSIY-STDTFSTPTVLFELGTS        1277
PhacH1        MEWNYDRLYKYEITHQLEEQNIHHNDRYDITVKVQALDGSDITEAANIPPPTVLFELGTS        1277
PocaH1        MPWKYDRLFKYEITRLLAELKLRADDPYRVSYTVYDLNGQELE-DNLFGNVTIVRDTGTG        1261
MetuMH        MPWEYDRLFKYEITDQLKEQGLEPLDRYDIKYTVFALDGSVVG-TDTFGQVTVVHTYGAG        1280
LisaH1        LPWSYDRLFKYEITDQLHEFDLQHGDNYDIHYVVYDLAGSSLG-DDLFGSSTTVYSAGHQ        1272
CrfoH1        LPWEYDRLYKYEITHELEDMGLEPQDRFDVQYHVYDLDNTDLG-DDVFGKAVIIYSAGQG        1261
RaveH1        MPWEYDRLFKYDITEQLEKFDLEPMDRYDIQYTVSDLNGQNLG-DDLFGNATIVYTPGLG        1274
OcvuHA        MPWKFDRVYKADITRQMEHLHLHYNDLYHIEYTLKDMSGAEVT--------------SLG        1256
TopaH1        MPWKFDRPYKSDITYVMDAMKLHYTDKYHVELRITDMTGAEVT--DLKLVTSVIYEPGIG        1241
EuscH1        MPWKFDRAYKADITHTMDEMKLHYTDKYHIEYTVTDLTGAEVA--GVSLSSSSVVYEPGLG        1269
SeofH1        MPWKFDRAYKSDITHVMDEMKLHYTDKYHVEYKISDMTGAEVT--DIKLESSVVFEPGLG        1268
SemaH         MPWKFDRAYKSDITHVMDEMKLHYTDKYHVEYKITDMTGAEIA--DIKLESSVIYEPGLG        1268
LijaH2        MPWIYDRAYKYEITSQLKALNLRFGDSFSIRSVILDLNGNEIP-PDTFPSATVDFVPAQG        1196
NunuH1        MPWTYNHLYKYEITDVLDSLHLHHEDRYSIKYEVYDLNNQNTG-TQPYPEPTILHEPSSG        1259
MecrH1        MPWAYDRVFKYDITEKLHDLKLHAEDHFYIDYEVFDLKPASLG-KDLFKQPSVIHEPRIG        1257
```

```
HacrH1         MAWAYDRVFKYDITNVLDENNLHFYDHIFISYEVFDLHGTSLG-TDIFHKANVIHDPGTG       1258
HarufH1        MAWAYDRVFKYDITNVLDENNLHFYDHIFISYEVFDLHGTSLG-TDIFHKANVIHDSGTG       1258
HadishaH1      MAWAYDRVFKYDITNVLDENNLHFYDHIFITYEVFDLHGTSLG-TDIFHKANVIHDSGTG       1258
HadivH1        MAWAYDRVFKYDTTNVLDENNLHFYDHIFITYEVFDLHGTSLG-TDIFHKANVIHDSGTG       1173
HaasH1         MAWAYDRLFKYDITHVLEENHLHFYDNIFIKYEVLDLKGASLG-TDLFHTANVIHYTGTG       1258
HarubH1        MAWAYDQLFKYDITHVLEENHLHFYDNIFIHYEVFDLKGTSLG-TDLFHTANVIHDKGTG       1258
               : * ::: :* : *  :    :.  *   :   :  :

CoasHaN        HLY--GQEYRPVVAAASQVRRNLESLSEGEIESLRAAFLAIQND--HSYEGIASYHGKPG       1330
HepoHaN        HIY--GQEYRPVVAAASQVRRNLESLSEGEIESLRAAFLAIQND--HSYEAIASFHGKPG       1329
HeluHaD        HLY--GREYRDAVTVASHVRKDLDTLTAGEIESLRSAFLDIQQD--HTYENIASFHGKPG       1326
ApcaH1         QLY--GQEYREAVTAASYVRRDLSTLNEGEVESLRAAFLSIQKD--DTYANIAAFHGKPG       1328
LystH1         NLY--GKEFRAVSTAASQIRRNLDSLSQGEIESLRAAFLSIKED--GTYEKIASFHGKPG       1333
PhacH1         NLY--GKEYRPSVTTISNIRRNLNDLSEGEVESLRSAFLSIQQD--GTYGDIAAFHGKPG       1333
PocaH1         VRR--GRVFVNVIETASHVRKNLEDLSPGETESLRSALLQMQKD--ESFQKIASFHGFPG       1317
MetuMH         HVE--RKSYGEELKASSHVRRNVDDLTGGEIESLKAALQKMEDD--GSFENIAEFHGAPG       1336
LisaH1         D----GNAYRQDVQASSHVRRNIDDLTEGEAESLRAALLHMEED--GSFEAIARFHGFPG       1326
CrfoH1         HQKGHEEDYLEEVQASSHVRRNLEDLTTGEAESLRSALRDMEED--GSFDAIARFHGYPG       1319
RaveH1         HMKGHEEDYRAEVRASSHVRRNLKSLTTGECESLRSALHDMEED--GSFEAIAQFHGSPG       1332
OcvuHA         H-YGEDRAWIEPVTSANRIRKNLEDLDVGEIESLRNAFKQIKMD--GTYERIAAFHGLPA       1313
TopaH1         N-FGEGRRWISPITSASRIRKNLLDFEDGEMESLRNAFKQMADE--GRYEEIASFHGVPA       1298
EuscH1         K-FGEGRAWIEPVTSASRIRKSLDDLSGGEIESLRNTFKQMTKD--GRYQEIASFHGLPA       1326
SeofH1         K-YGEGRAWIEPVTSAVRIRKNLNDLSGGEL-ILRNYIKQMTKD--GSYQQIAAFHGLPA       1324
SemaH          K-YGEGRAWIEPVTSAVRIRKNLNNLSGGEIESLRNTFKQMTKD--GRYQQIASFHGLPA       1325
LijaH2         T-YGTEAEWVEPITDASRIRKDLATLTVGETESLRNAFLRLQKDKNRGYEYIAGFHGLPT       1255
NunuH1         A--GVDHEYEEVVTTASHIRKDINNLNPGEIESLRAAFGQIRND--GTYAKIAQAHGKPG       1315
MecrH1         H--HEGEVYQAEVTSANRIRKNIENLSLGELESLRAAFLEIEND--GTYESIAKFHGSPG       1313
HacrH1         S--REHDKYVEEVTGASHIRKNLNDLNLGEMESLRAAFLHIQND--GTYESIAQYHGKPG       1314
HarufH1        S--REHDKYVEEVTGASHIRKNLNDLNLGEMESLRAAFLHIQND--GTYESIAQYHGKPG       1314
HadishaH1      S--REHDRYVEEVTGASHIRKNLNDLNLGEMESLRAAFLHIQND--GTYESIAQYHGKPG       1314
HadivH1        S--REHDRYVEEVTGASHIRKNLNDLNLGEMESLRAAFLHIQND--GTYESIAQYHGKPG       1229
HaasH1         T--RR-ENYVEEVTGASHIRKNLNELNTGEMESLRAAFLHIQAD--GTYESIAKYHGKPG       1313
HarubH1        T--REHHTYVEEVTGASHIRKNLNDLNTGEMESLRAAFLHIQND--GTYESIAQYHGKPG       1314
                       :         :*:.:  :  **   *:  :  :  :    :  **  ** *

CoasHaN        LCEH-QGR-KVACCVHGNPTFPSWHRLYVELVEHALLSHGSSVAVPYWDWISPIKKLPKL       1388
HepoHaN        LCEH-EGR-KVACCVHGDPTFPSWHRLYVELVEHALLSHGSSVAVPYWDWISPIKKLPKL       1387
HeluHaD        LCQH-EGH-KVACCVHGMPTFLSWHRLYVEQVEEALLDHGSSVAVPYFDWISPIQKLPDL       1384
ApcaH1         LCEL-NGR-KVACCVHGMATFPAWHRLYVEQVEEALLGRGSNVGFPYWDWTQPITELPKL       1386
LystH1         KCKDSAGR-TVACCVHGMPTFPAWHRLYVEQVEEALLGRGSSVAVPYWDWTTAFTKLPDL       1392
PhacH1         LCEH-DGH-KIACCVHGMPTFPAWHRLYVEQVEEALLSRGSSVAVPYWDWTSPFQKLPSL       1391
PocaH1         MCKH-NNR-SVACCVHGSPTFLHWHRLYVEQVENCLLARGSAVSVPYWDWTQPIRQLPTL       1375
MetuMH         LCEL-NGK-KVGCCVHGMATFPHWHRLYVEQVENALLSHGSAVSIPYWDWTKPITKLPDI       1394
LisaH1         LCEL-NGQ-KKACCVHGSPTFPHWHRLYVEQVENALLSHGSAVSVPYWDWTQPIRKLPEL       1384
CrfoH1         LCEH-DGH-KAACCVHGSPAFPHWHRLYVEQVENSLLSHGSAVSVPYWDWTQPIKKLPAL       1377
RaveH1         LCDH-KGK-QVGCCVHGAPTFPHWHRLFVEQVENALLDHGSAVSVPYWDWTEDFDHLPKL       1390
OcvuHA         KCPNEDGSKVYTCCLHGMPNFLHWHRLYTVSVEHELLARGSSVAVPYWDWLKSFDELPKL       1373
TopaH1         QCPSEDGTMVHTCCLHGMPVFPHWHRLYVSLVEDELLARGSGVAVPYWDWVEPFDELPRL       1358
EuscH1         QCPNADGTVVFTCCLHGMPTFPHWHRLYLSLVEDELLARGAGVAVPYWNWIEPFDRLPAF       1386
SeofH1         QCPSEDGTTVHTCCLHGMPTFPHWHRLYVALVEDELLSRGSRSGRPYWDWIDPFDRLPDF       1384
SemaH          QCPSEDGTTVHTCCLHGMPTFPHWHRLYVALVEDELLSRGAGVAVPYWDWIDPFDRLPEF       1385
LijaH2         KCPSPDNP-KYACCLHGMPVFPHWHRLYVLQVENALLAIGSSIAVPYWDWTKRIDRLPPL       1314
NunuH1         LCNG------KGCCVHGMATFPHWHRLYEVQVENALLEHGSAVAVPYWDWTEPIKSLPHL       1369
MecrH1         LCQLNGNP--ISCCVHGMPTFPHWHRLYVVVVENALLKKGSSVAVPYWDWTKRIEHLPHL       1371
HacrH1         KCELNHHS--IACCVHGMPTFPQWHRLYVAQVENALLKKGSSVAVPYWEWTVPIDHLPGL       1372
HarufH1        KCELNHHS--IACCVHGMPTFPQWHRLYVAQVENALLKMGSSVAVPYWEWTAPIEHLPGL       1372
HadishaH1      KCELNHRS--IACCVHGMPTFPQWHRLYVAQVENALLKMGSSVAVPYWEWTAPIDHLPGL       1372
HadivH1        KCELNHRS--IACCVRGMPTFPQWHRLYVAQVENALLKMGSSVAVPYWEWTAPIDHLPGL       1287
HaasH1         KCQLNGRN--IACCVHGMPTFPQWHRLYVAQVENALLDRGSGVAVPYWEWTAPIDHLPHF       1371
HarubH1        KCELNHRS--IACCVHGMPTFPQWHRLYVAQVENALLKRGSGVAVPYWEWTSPIDHLPHF       1372
                *          **::*   *  ****:   **. **  *:  . **::*    : ** :

CoasHaN        ISKSTYYNSRQQRFDPNPFFSGRIA--GENAVTTRDPQPELFNNDYFLEQALFALEQDHY       1446
HepoHaN        ISKSTYYNSRQQRFDPNPFFSGRIA--GENAVTTRDPQPELFNNDYFLEQALFALEQDHY       1445
HeluHaD        ISKATYYNSREQRFDPNPFFSGKVA--GEDAVTTRDPQPELFNNDYFYEQALYALEQDNF       1442
ApcaH1         INDATYFNSRTQKFEPNPFFSGKVL--GEDAVTTRDPQPQLFNNDYFYQQTLFALEQTNF       1444
LystH1         INQATYFNSRQQRFEPNPFFSGSIA--GEGAVTTRDPQPELFNNDDLYEQVLYALEQDHF       1450
PhacH1         ISKATYYNSRQQRFDPNPFFSGKIK--GEDAVTTRDPQPELFDNSELYEEVLFALEQDNF       1449
PocaH1         ISSPSFFNSRTYNKESNPFFRGEIP--DTDAFTTRDPQPQLFNSNYFLDNVLLALEQTSF       1433
MetuMH         IAQETYFDSRSQTMQNNPFFRGPIRTPDVNDYTTRDPQPELFNNDYFLQQTLLALEQTSY       1454
LisaH1         IDAPTYFNSRSHTKDTNPFFRGKIS--DTDKYTTRDPRPDLFNNNYFLDNVLLALEQTSF       1442
CrfoH1         INDATYYDSRAHAKLENPFFRWKIP--GTDMYTSRDPRPDLFDSDYFLNNALLALEQTSY       1435
RaveH1         LAMPTYYNSRMHSKDVNPFFRWKVS--GTDEYTTRDVRFDLFNAEYFLDNILLALEQTSY       1448
OcvuHA         INEATFYNSRTLHIESNPFFNGKIS--FENTETDRDPQPNLFGNSYFYEHALFAFEQTDF       1431
TopaH1         INEATFYNSRTLQIEPNPFFKGKIS--FENAETDRDTQPELFGNRYLYDHTLFVFEQTDF       1416
EuscH1         FNDATYYNSRTLHIESNPFFNGAID--FASTVTDRDAHELLYGNKNLYDEALFVLEQTDF       1444
SeofH1         FNDATYYNSRTLHIESNPFFRGSMS--FANTLTDRDAQDVIYNNHEFYDKALLVLEQTDF       1442
```

```
SemaH           FNDATYYNSRTLHIESNPFFRGSVD--FASTLTDRDAQDVIYNNHEFYDKALLVLEQTDF        1443
LijaH2          VSDSTFYNSRTLRLDPNPFFRGEIS--FENAVTTRDPQPELYNNDYFYNQVLLAFEEDDF        1372
NunuH1          LSDPTFFNSRSHEFDVNPFFSGDIP--GENSDTSRDPQPRLFNNDYFYDNMLLAFEQTSF        1427
MecrH1          ISDATYYNSRQHHYETNPFHHGKIT--HENEITTRDPKDSLFHSDYFYEQVLYALEQDNF        1429
HacrH1          IDDATYFNSRQQRYDPNPFFRGKIS--FENAVTTRDPQEELFNSEYMHNNVLLALEQDNY        1430
HarufH1         IDDATYFNSRQQRYDPNPFFRGKIS--FENAVTTRDPQEELFNSEYMHNNVLLALEQDDY        1430
HadishaH1       IDDATYFNSRQQRYDPNPFFRGKIS--FENAVTTRDPQEELFNSEYMQNNVLLALEQDNY        1430
HadivH1         IDDATYFNSRQQRYDPNPFFRGKIS--FENAVTTRDPQEELFNSEYMQNNVLLALEQDNY        1345
HaasH1          IDDATYFNSRKQRYDPNPFFRGKIT--FENAVTTRDPQEGLFNSDYMYENVLLALEQENY        1429
HarubH1         IDDATYFNSRQQRYDPNPFFRGRIT--FENAVTTRDPQEGLFNSDYMYENVLLALEQENY        1430
                .   ::::**      ***.   :     .  * ** :  ::    : :. * .:*:  :

CoasHaN         CDFEIQFEILHNALHSWLGGHAFYSMASLDYAAFDPVFFLHHANTDRIWAVWQELQRYRG        1506
HepoHaN         CDFEIQFEILHNALHSWLGGHALYSMASLDYSAFDPVFFLHHANTDRIWAIWQELQRYRG        1505
HeluHaD         CDFEIQFEVLHNALHSWLGGHAKYSFSSLDYTAFDPVFFLHHANTDRLWAIWQELQRYRG        1502
ApcaH1          CDFEIQFELIHNALHSWLGGRAEYSFSSLDYTAFDPVFFLHHANTDRIWAIWQELQRYRG        1504
LystH1          CDFEIQFEIIHNALHSLLGGRAQYSLSSLDYSAFDPVFFLHHANTDRIWAIWQELQRYRG        1510
PhacH1          CDFEIQFELVHNALHSLLGGRAQYSLSSLDYSAFDPVFFLHHANTDRIWAIWQELQRYRG        1509
PocaH1          CDFEVQLEVTHNALHSWLGGRDKHSMSSLDYSAFDPAFFLHHANTDRLWAIWQALQQYRG        1493
MetuMH          CDFEAQFEVTHNAFHSFLGGRGKYSLSTLDYSAFDPVFFLYHANTDRIFAIWQALQSYRG        1514
LisaH1          CDFEVQFEIVHNALHSWLGGRGKFSMSGLDYSAFDPVFFLHHANTDRIWAIWQALQRHRG        1502
CrfoH1          CDFEVQFEILHNALHSFLGGRGKYSLSSLDYSAFDPVFFLHHANVDRIWAIWQALQKIRG        1495
RaveH1          CDFEVQFEVSHNAIHSFLGGRGKVSMSTLDYSAFDPIFFLHHANMDRIWAIWQALQKHRD        1508
OcvuHA          CEFATHLEVLHNSLHSWLGGRDPHSMSSLDYAAYDPAFFLHHSNMDRLWAIWQELQRYRK        1491
TopaH1          CEFEVHYEVLHNTIHSWLGGRDVHSMSSLDYAAYDPVFFLHHSNVDRLWAIWQELQRYRK        1476
EuscH1          CEFEVQLEVLHNRIHSLLGGREVYSMASLDFAAYDPVFFLHHSNVDRLWAIWQELQRYRK        1504
SeofH1          CEFEVQLEVLHNRIHSMLGGREIYSMSSLDYAAYDPVFFLHHSNVDRLWAIWQELQRYRK        1502
SemaH           CEFEVQLEVLHNRIHSMSGGREIYSMSSLDYAAYDPVFFLHHSNVDRLWAIWQELQRYRK        1503
LijaH2          CNFEVQFEIAHNALHAWIGGRDPYSMSTLDYTAYDPIFFLHHTNVDRLWAIWQELQRYRK        1432
NunuH1          CDFEIQMELVHNALHSWIGGRAHYSLSSLDYTAFDPAFFIHHANVDRLWAIWQELQRYRK        1487
MecrH1          CDFEIQLEILHNALHSLLGGKGKYSMSNLDYAAFDPVFFLHHATTDRIWAIWQDLQRFRK        1489
HacrH1          CDFEIQFELIHNALHSLLGGKGQYSMSSLDYSAYDPVFFLHHANTDRLWAIWQELQRYRG        1490
HarufH1         CDFEIQFELVHNALHSLLGGKGQYSMSSLDYSAFDPVFFLHHANTDRLWAIWQELQRYRG        1490
HadishaH1       CDFEIQFELVHNALHSLLGGKGQYSMSSLEYSAFDPVFFLHHANTDRLWAIWQELQRYRG        1490
HadivH1         CDFEIQFELVHNALHSLLGGKGQYSMSSLDYSAFDPVFFLHHANTDRLWAIWQELQRYRG        1405
HaasH1          CDFEIQFELVHNALHSLLGGKGQYSLSSLDYSAFDPVFFLHHANTDRLWAIWQELQRYRE        1489
HarubH1         CDFEIQFELVHNALHSMLGGKGQYSMSSLDYSAFDPVFFLHHANTDRLWAIWQELQRYRD        1490
                *:*  : *: ** :*:  **:   *:: *:::*:** **::*:. **::*:** **  *

CoasHaN         LQYNEADCAINLMRKPLEPFNRV--TNTDEVTRKNSRPVDTFDYRNNLHYEYDTLEFNHL        1564
HepoHaN         LQYNEADCAINLMKKPLQPFNRT--TNTDEVTRKYSRPVDTFDYRNNLHYEYDTLEFNHL        1563
HeluHaD         LPYNEADCAINLMRKPLQPFQDKK-LNPRNITNIYSRPADTFDCRNHFHYEYDTLELNHQ        1561
ApcaH1          LPYNEADCAINLMRKPLQPFNNDD-FNHGDATNRYSRPADTFDYRNHFQYEYDTLTFNHM        1563
LystH1          LKYNEADCAINQMRTPLRPFNDDQ-LNHGDLTHRYSRPADTFDYRNHFHYEYDTLEFNHM        1569
PhacH1          LLYNEADCAINLMRKPLQPFNDGG-LNHGDYTHRYSRPADTFDYRNHFHYEYDNLEFNHM        1568
PocaH1          LPSHESDCSLHLMRRPLQPFDSTS-QNPVEITNQFSRPADVFDYTNHFSYRYDNLLFNSW        1552
MetuMH          LHWDEADCALNHMRDGLHPFDVKA-QNEFDITHKYSRPVDVWDYSDHLQYHYDNLLLNDW        1573
LisaH1          LSFEESDCALNLMRKPLHPFDDKT-QNEFDLTNHYSRPADLFDYSGHLDYHYDNLKFNSW        1561
CrfoH1          LPFDESDCALNIMGTPLHPFDDKE-ENQFDLTNKYSRPIDAFDYSNHFDYHYDTLKFNSW        1554
RaveH1          LPFDETDCAVNLMSKPLHPFDNKD-INHFGLTYKHSRPADVWDYSRHLDYHYDNLQLHGW        1567
OcvuHA          LPYNSANCALGLLNQPMRPFSNKT-ANSNLLTFTHSRANDVFDYQNVLHYKYDSLAFNGL        1550
TopaH1          LSYNEANCALPLMNQPMRPFSNST-ANNDRLTFTNSRPNDVFDYQNVLHYKYDTLNFAGL        1535
EuscH1          LPYNEANCALPLLNEPMRPFSNST-ANQDRLTFTNSRPNDVFDYQNVLHYKYDTLTFEGL        1563
SeofH1          LSYNEANCALPLLNEPMRPFSNKT-ANHDRLTFTNSRPNDVFDYQNVLHYKYDTLKFEGL        1561
SemaH           LSYNEANCALPLPNQPMRPFSNKT-ANHDRLTFTNSRPNDVFDYQNVLHYKYDTLEFEGL        1562
LijaH2          KPSNTAFCALPYMQTPIKPFSFDRSINYDWMTRVYSRPSEAFDYQNNFGYKYDTLEFNGM        1492
NunuH1          LPYNKADCSINLMRKPLYPFAGD--ENKDTLTKKNSHPQDVFDYRNNLHYKYDNLEFHHM        1545
MecrH1          RPYREANCAIQLMHTPLQPFDKSD--NNDEATKTHATPHDGFEYQNSFGYAYDNLELNHY        1547
HacrH1          LPYEEANCAINLMHQPLKPFSDPN-ENHDNITLEHSKPQDCFDYRNNFGYKYDNLEFHHL        1549
HarufH1         LPYEEANCAINLMHQPLMPFSDPH-ENHDNVTLKHSKPQDCFDYRNNFGYKYDNLEFHHL        1549
HadishaH1       LPYEEANCAINLMHQPLMPFSDPN-ENHDNVTLKHSKPQDCFDYRNNFGYKYDNLEFHHL        1549
HadivH1         LPYEEANCAINLMHQPLMPFSDPN-ENHGNVTLKHSKPQDCFDYRNNFGYKYDNLEFHHL        1464
HaasH1          LPYEEANCAINLMHQPLKPFCDPH-ENHDNITLKYSKPQECFDYQNHFGYKYDNLEFHHL        1548
HarubH1         LPYEEANCAINLMHQPLKPFSDPH-ENHDNITLKYSKPQDGFDYQNHFGYKYDNLEFHHL        1549
                     : *::      : **      *    *   :   : ::    : * **.* :

CoasHaN         SIPQLEELIQS-RKRNGRVFAGFLIHNIGLSADVDVYVCVSVGKYGEQDCDHKAATFSVL        1623
HepoHaN         SIPQLEELLQS-RKRNGRVFAGFLIHNIGLSADVDVYVCVTVGQFGEQDCNHKAGTFSVL        1622
HeluHaD         TVPQLENLLKR-RQEYGRVFAGFLIHNIGLSADVTVYVCVPSGPKGKNDCNHKAGVFSVL        1620
ApcaH1          TIPQLENLLHQ-RQESGRVFAGFLLHNIGASADVEIYICVPTGPRGKKNCGTRAGVFSVL        1622
LystH1          TVPQLEALLKK-RKESGRVFAGFLLHNIGLSAVANIYVCVPSGPRGKKSCNHPAGSFSIL        1628
PhacH1          TIPQLEALLKK-RKQNGRVFAGFLLHNIGLSAEVDIYVCVPAGPRGKKSCDHKAGSFYIL        1627
PocaH1          TLPQLEAVLRN-QRSRDRLFAGFLLSNIGTSADVLIFICVATGN-GGRSCNHPAGEFSIL        1610
MetuMH          TIPQLEEVLKT-QRSRDRMFAGFLLHNIGTSADVELHICVATGN-GDRNCNNPAGKFSIL        1631
LisaH1          TVPQLDEVLKQ-QRSRDRLFAGFLLHNIGTSADVEIDVCVATGN-GAQSCNHPAGKFALL        1619
CrfoH1          TIPQLEQVLEK-QRSRDRVFAGFLLHNIGTSADVEIDVCVATGN-GAKSCNHPAGKFAIL        1612
RaveH1          SIAQLDEVLKK-QRSRDRMFAGFLLHNIGTSAVVEIDVCVDTGD-GGRSCHHPAGKFSIL        1625
```

```
OcvuHA          TVIQLENLLQHNRHQQDRVFAAFLLHGVKASADVRIYICAPTGR-NQENCANYAGVFSIL        1609
TopaH1          SIPQLERILQK-NQGRDRIFAGFLLHGIKASADVRIYICVPTGI-GEENCGNYAGIFSVL        1593
EuscH1          SIPELEALINK-RQDHDRVFAGFLLHGIKTSAEVHIYICVPTGV-NEENCGNFVGTFSVL        1621
SeofH1          SIPELEALLNK-RKSHDRVFAGFLLHGIKTSADISIYICVPTGV-NEENCGNYVGTFSAL        1619
SemaH           SIPELEALLNK-RKSHDRVFAGFLLHGIKTSADISIYICVPTGV-NAENCGNYVGTFSAL        1620
LijaH2          SIPKLQSLLEV-RKSQDRVFAGFLLSGIQTSANVRIFICLPR-T-GYTDCSNYAGIFSVL        1549
NunuH1          SIPELEATLDA-RKSRDRVFAGFLLHNIGTSADVEIYICVPKGD-GYKDCNNYAGVFSVL        1603
MecrH1          SIPQLDHMLQE-RKRHDRVFAGFLLHNIGTSADGHVFVCLPTGE-HTKDCSHEAGMFSIL        1605
HacrH1          SIPSLDATLKA-RKEHDRVFAGFLLHNIGTSADISIYICQPDGS-GGNDCSHKAGTFYVL        1607
HarufH1         SIPSLDATLKA-RKEHDRVFAGFLLHNIGTSADISIYICQPDGS-GGNDCSHKAGTFYVL        1607
HadishaH1       SIPSLDATLKA-RKEHDRVFAGFLLHNIGTSADISIYICLPDGS-GGNDCSHKAGTFYVL        1607
HadivH1         SIPSLDATLKA-RKEHDRVFAGFLLHNIGTSADISIYICLPDGS-GGNDCSHKAGTFYVL        1522
HaasH1          SIPSLDATLKQ-RKQHDRVFAGFLLHNIGTSADITIYICLPNEH-HGHDCGHEAGTFYIL        1606
HarubH1         SIPSLDVTLKQ-RKHHDRVFAGFLLHNIGTSADITIYICLPDGR-RGHDCSHEAGTFYLL        1607
                :: .*:  :   .:  .*:**.**: .:  **   : :*         .*   .. *  *

CoasHaN         GGET-EMPFEFNRLYKQDITRTVRELGLKLDYAANFHLLVEIKAPNGSQLDPHILPDPSI        1682
HepoHaN         GGET-EMPFEFNRLYKQDITRTVRELGLKLDNAANFHLLVEIRAPNGSQLDPHILPDPSI        1681
HeluHaD         GGEL-EMPFTFDRLYMLQITDTIKQLGLKVNNAASYQLKVEIKAVNGTLLDPHILPDPSI        1679
ApcaH1          GGQLEEMPFVFDRLYKYDITRAVRDLGLRPDSGAKFDLKINIHAVNGSYLNPSLLPAPTV        1682
LystH1          GGEL-EMPFVFDRLYKYDITKTVRDLGLKLDNAANFELRVDIKAVNGSLLDSDILPKPTI        1687
PhacH1          GGEL-EMPFVFDRLYKHDITSTIKALGLKLDSAANFELKIDIKAVNGSLLSADILPTPTI        1686
PocaH1          GGET-EMPFTFDRLYKYDISEPIRQLGLKLDSAAEFELEIKIRAFNGSYLEPDLVDKPSI        1669
MetuMH          GGEY-EMPFTFDRLYKYDISDSIRKLGLKLDSGANFDLKVQIHAYNGSYLDPSLLKEPTI        1690
LisaH1          GGEY-EMPFTFDRLYKFDISDTVRKLGLRLDSAADFDLEIKIKSYNGSYIDSNLLGRPSI        1678
CrfoH1          GGEY-EMPFTFDRLYKYDISDTVRKLGLRLDSAADFDVQIKIFAYNGSYIDASLLHRPTI        1671
RaveH1          GGEY-EMPFTFDRLYKHDITDAIRKLGLRLDSAADFDLEVKIHSYNGSYIDASLLHPPSI        1684
OcvuHA          GGES-EMPWRFTHLFRYEITDSLMKLGLNHNS--HFRISTEVRAVNGSKIEKKIFPEPTI        1666
TopaH1          GGET-EMPWQFDRLFRYEITDELKKLGLNQNS--HFRVEMELTAVNGSKITQKIFPNPTI        1650
EuscH1          GGES-EMSWNFDRLYRYEITDDLMLLGLNHNS--HFRIAVEIITANRSMADSNIFPTPTV        1678
SeofH1          GGES-EMPWNFDRLFRYEITDELMLLGLNHNS--HFRISIEIMTMNRSIASSKIFPNPTI        1676
SemaH           GGET-EMPWNFDRLFRYEITDELMLLGLNHNS--HFRISIEIMTMNRSIASSKIFPNPTI        1677
LijaH2          GGET-EMPWSFDRLYKYDITPALSDLGLTADS--NFDVSIEIIARNGSSLSPDLFPRPTI        1606
NunuH1          GGEV-EMPFVFDRLYKYDITPTIHSLGLNPDA-SDFDLKVAIHGVNGAILSSDALPRPTI        1661
MecrH1          GGQT-EMSFVFDRLYKLDITKALKKNGVHLQG--DFDLEIEITAVNGSHLDSHVIHSPTI        1662
HacrH1          GGET-EMPFVFDRLYKFEITEALHKLGVKLHG-GIFDLELDIVAYNGSHLDSHIF-DPTI        1664
HarufH1         GGET-EMPFVFDRLYKFEITEALHKLGVKLHG-GLFDLELDIVAYNGSHLDSHIF-DPTI        1664
HadishaH1       GGET-EMPFVFDRLYKFEITEALHKLGVKLHG-GLFDLELDIVAYNGSHLDSHIF-DPTI        1664
HadivH1         GGET-EMPFVFDRLYKFEITEALHKLGVKLHG-GLFDLELDIVAYNGSHLDSHIF-DPTI        1579
HaasH1          GGET-EMPFIFDRLYKFEITKALQKLGVKLHG-GVFDLELEIRAYNGSYLDPHTF-DPTI        1663
HarubH1         GGET-EMPFIFDRLYKFEITKPLQKLGVKLHG-GVFELELEIRAYNGSYLDSHTF-DPTI        1664
                **:  ** : * :*:  :*:  :   *:  .    : :   :   * :      .  *::

CoasHaN         IYIPGTDEEQD-------------------------------------------------        1693
HepoHaN         IYIPGTDEVED-------------------------------------------------        1692
HeluHaD         IFEPGTKERQA-------------------------------------------------        1690
ApcaH1          LYVPGSNERQE-------------------------------------------------        1693
LystH1          LFEPGTGERQK-------------------------------------------------        1698
PhacH1          QFVPGTTEVQK-------------------------------------------------        1697
PocaH1          IFLPGTGKRQT-------------------------------------------------        1680
MetuMH          IFEPGENTFQT-------------------------------------------------        1701
LisaH1          IFMPGEGKHQD-------------------------------------------------        1689
CrfoH1          IFEPGQGQTQD-------------------------------------------------        1682
RaveH1          IFQPGEGKTQD-------------------------------------------------        1695
OcvuHA          IFVPKHGA----------------------------------------------------        1674
TopaH1          IFVPSDVEFEEDTWRDVVTSANRIRRNLKDLSKEDMFSLRAAFKRMTDDGRYEEIAAFHG        1710
EuscH1          IFVPTADSEHKESWGGVITSANRIRKNLKDLKKEEMICFADAFRLMAADGRYEEIAAFHG        1738
SeofH1          IFVPGADSGGKDAWGGVITSANRIRRNLKDLNKDQMICFAEAFRSMAESGRYEEIAAFHG        1736
SemaH           IFVPGADSEGKDAWGGVITSANRIRRNLKDLNKDQMICFAEAFRSMAESGRYEEIAAFHG        1737
LijaH2          VYVPGTGHEMQS------------------------------------------------        1618
NunuH1          LLQPGRGAAAP-------------------------------------------------        1672
MecrH1          LFEAGTDSAHT-------------------------------------------------        1673
HacrH1          IFEPGKDTHVL-------------------------------------------------        1675
HarufH1         IFEPGIDTHVL-------------------------------------------------        1675
HadishaH1       IFEPGIDTHVL-------------------------------------------------        1675
HadivH1         IFEPGIDTHVL-------------------------------------------------        1590
HaasH1          IFEPGTDTHIL-------------------------------------------------        1674
HarubH1         IFEPGTDTHIL-------------------------------------------------        1675


CoasHaN         ------------------------------------------------------------        1693
HepoHaN         ------------------------------------------------------------        1692
HeluHaD         ------------------------------------------------------------        1690
ApcaH1          ------------------------------------------------------------        1693
LystH1          ------------------------------------------------------------        1698
PhacH1          ------------------------------------------------------------        1697
PocaH1          ------------------------------------------------------------        1680
```

```
MetuMH          ------------------------------------------------------------   1701
LisaH1          ------------------------------------------------------------   1689
CrfoH1          ------------------------------------------------------------   1682
RaveH1          ------------------------------------------------------------   1695
OcvuHA          ------------------------------------------------------------   1674
TopaH1          LPAQCPNADGSNIHTCCLHGMPTFPHWHRLYLSLVENELLARGSDVAVPYWDWIEPFDSL   1770
EuscH1          LPAQCPDETGDNVYTCCLHGMPVFPHWHRLYLALVENELLARGSCIAVPYWDWIEPFDEL   1798
SeofH1          LPAQCPDESGDKVFTCCLHGMPVFPHWHRLYLALVENELMSRGSCIAVPYWDWIEPFDVL   1796
SemaH           LPAQCPDESGDKVFTCCLHGMPVFPHWHRLYLALVENELMARGSCIAVPYWDWIEPFDVL   1797
LijaH2          ------------------------------------------------------------   1618
NunuH1          ------------------------------------------------------------   1672
MecrH1          ------------------------------------------------------------   1673
HacrH1          ------------------------------------------------------------   1675
HarufH1         ------------------------------------------------------------   1675
HadishaH1       ------------------------------------------------------------   1675
HadivH1         ------------------------------------------------------------   1590
HaasH1          ------------------------------------------------------------   1674
HarubH1         ------------------------------------------------------------   1675

CoasHaN         ------------------------------------------------------------   1693
HepoHaN         ------------------------------------------------------------   1692
HeluHaD         ------------------------------------------------------------   1690
ApcaH1          ------------------------------------------------------------   1693
LystH1          ------------------------------------------------------------   1698
PhacH1          ------------------------------------------------------------   1697
PocaH1          ------------------------------------------------------------   1680
MetuMH          ------------------------------------------------------------   1701
LisaH1          ------------------------------------------------------------   1689
CrfoH1          ------------------------------------------------------------   1682
RaveH1          ------------------------------------------------------------   1695
OcvuHA          ------------------------------------------------------------   1674
TopaH1          PGLISDETYKHPKTNEDIENPFHHGKISFADAVTVRKPRDQLFNNRYLYEHALFAFEHTD   1830
EuscH1          PPLINDLTYYNPKTDKTLPNPFLKGSVSFENTHTQRTLSSDIFGNMNMYDHALFALEQTD   1858
SeofH1          PVLINDLTYYNPQTNKVHPNPFLKGNISFASSETHRSPTKELYGNRYLYDHALFALEQTD   1856
SemaH           PVLINDLTYYNPKTNKVHPNPFLKGNISFASTETHRSPTKELFGNRYLYDHALFALEQTD   1857
LijaH2          ------------------------------------------------------------   1618
NunuH1          ------------------------------------------------------------   1672
MecrH1          ------------------------------------------------------------   1673
HacrH1          ------------------------------------------------------------   1675
HarufH1         ------------------------------------------------------------   1675
HadishaH1       ------------------------------------------------------------   1675
HadivH1         ------------------------------------------------------------   1590
HaasH1          ------------------------------------------------------------   1674
HarubH1         ------------------------------------------------------------   1675

CoasHaN         ------------------------------------------------------------   1693
HepoHaN         ------------------------------------------------------------   1692
HeluHaD         ------------------------------------------------------------   1690
ApcaH1          ------------------------------------------------------------   1693
LystH1          ------------------------------------------------------------   1698
PhacH1          ------------------------------------------------------------   1697
PocaH1          ------------------------------------------------------------   1680
MetuMH          ------------------------------------------------------------   1701
LisaH1          ------------------------------------------------------------   1689
CrfoH1          ------------------------------------------------------------   1682
RaveH1          ------------------------------------------------------------   1695
OcvuHA          ------------------------------------------------------------   1674
TopaH1          FCDFEVHFEVLHNSIHSWIGGPNPHSMSSLDFAAYDPIFFLHHSTVDRLWAIWQDLQRYR   1890
EuscH1          FCEFEIQYEVLHNTIHALIGGASKYSMSSLDYAAYDPIFFLHHSNVDRLWAVWQELQRYR   1918
SeofH1          FCEFEVHFEVLHNTIHSWIGGPSRHSMSSLDFAAYDPVFFLHHSNVDRLWAIWQELQRYR   1916
SemaH           FCEFEVHYEVLHNTIHSWIGGPSKHSMSSLDFTAYDPVFFLHHSNVDRLWAIWQELQRYR   1917
LijaH2          ------------------------------------------------------------   1618
NunuH1          ------------------------------------------------------------   1672
MecrH1          ------------------------------------------------------------   1673
HacrH1          ------------------------------------------------------------   1675
HarufH1         ------------------------------------------------------------   1675
HadishaH1       ------------------------------------------------------------   1675
HadivH1         ------------------------------------------------------------   1590
HaasH1          ------------------------------------------------------------   1674
HarubH1         ------------------------------------------------------------   1675

CoasHaN         ------------------------------------------------------------   1693
HepoHaN         ------------------------------------------------------------   1692
HeluHaD         ------------------------------------------------------------   1690
```

```
ApcaH1        ------------------------------------------------------------   1693
LystH1        ------------------------------------------------------------   1698
PhacH1        ------------------------------------------------------------   1697
PocaH1        ------------------------------------------------------------   1680
MetuMH        ------------------------------------------------------------   1701
LisaH1        ------------------------------------------------------------   1689
CrfoH1        ------------------------------------------------------------   1682
RaveH1        ------------------------------------------------------------   1695
OcvuHA        ------------------------------------------------------------   1674
TopaH1        KLDYNVANCALNLLNDPMRPFNNKTANQDHLTFTNSRPNDVFDYQNSLNYKFDSLSFSGL   1950
EuscH1        KLEYNTATCAKNSLNKPMRPFSNSTANHDRLTYVNSKPNDVFDYQNVLHYKYETIEFSGL   1978
SeofH1        KLDYNTATCAKNYLNKPMRPFSNSTANHDRLTYVNSKPNDVFDYQNVLHYKYDSISFSGL   1976
SemaH         KLDYNTATCAKNYLNKPMRPFGNSTANHDRLTYVNSKPNDVFDYQNVLHYKYDSISFSGL   1977
LijaH2        ------------------------------------------------------------   1618
NunuH1        ------------------------------------------------------------   1672
MecrH1        ------------------------------------------------------------   1673
HacrH1        ------------------------------------------------------------   1675
HarufH1       ------------------------------------------------------------   1675
HadishaH1     ------------------------------------------------------------   1675
HadivH1       ------------------------------------------------------------   1590
HaasH1        ------------------------------------------------------------   1674
HarubH1       ------------------------------------------------------------   1675

CoasHaN       ------------------------------------------------------------   1693
HepoHaN       ------------------------------------------------------------   1692
HeluHaD       ------------------------------------------------------------   1690
ApcaH1        ------------------------------------------------------------   1693
LystH1        ------------------------------------------------------------   1698
PhacH1        ------------------------------------------------------------   1697
PocaH1        ------------------------------------------------------------   1680
MetuMH        ------------------------------------------------------------   1701
LisaH1        ------------------------------------------------------------   1689
CrfoH1        ------------------------------------------------------------   1682
RaveH1        ------------------------------------------------------------   1695
OcvuHA        ------------------------------------------------------------   1674
TopaH1        SIPRLDDLLESRQSHDRVFAGFWLSGIKASADVNIHICVPIGVEHEDCDNYAGTFAVLGG   2010
EuscH1        NIPQLENVLKNNKAHDRVFVGYSLHGIKASAFFRFYICVPVGA-EKNCDHFGGTFSVLGG   2037
SeofH1        NIPQLENVLTNNKAHDRVFVGFSLHGIKASADVRIYICVPVGQEEKNCDHYAGIFSVLGG   2036
SemaH         NIPQLENVLTNNKAHDRVFVGFSLHGIKASADVRIYICVPVGQEEKNCDHYAGIFSVLGG   2037
LijaH2        ------------------------------------------------------------   1618
NunuH1        ------------------------------------------------------------   1672
MecrH1        ------------------------------------------------------------   1673
HacrH1        ------------------------------------------------------------   1675
HarufH1       ------------------------------------------------------------   1675
HadishaH1     ------------------------------------------------------------   1675
HadivH1       ------------------------------------------------------------   1590
HaasH1        ------------------------------------------------------------   1674
HarubH1       ------------------------------------------------------------   1675

CoasHaN       ------------------------------------------------------------   1693
HepoHaN       ------------------------------------------------------------   1692
HeluHaD       ------------------------------------------------------------   1690
ApcaH1        ------------------------------------------------------------   1693
LystH1        ------------------------------------------------------------   1698
PhacH1        ------------------------------------------------------------   1697
PocaH1        ------------------------------------------------------------   1680
MetuMH        ------------------------------------------------------------   1701
LisaH1        ------------------------------------------------------------   1689
CrfoH1        ------------------------------------------------------------   1682
RaveH1        ------------------------------------------------------------   1695
OcvuHA        ------------------------------------------------------------   1674
TopaH1        ETEMPWAFDRLFRYEISDEMKKLQLTEDSKFRLTTNIIASNGSKVSNDIFPTPTVIFVPK   2070
EuscH1        ETEMPWQFDRPFRYDITPILNELNLNYKSDFTIKTEVIGVNGTKLNEKLFHDPSIIFVPK   2097
SeofH1        ETEMPWQFDRLFRYEISHALNALELTHKSDFTIKVEVTAANGTRINEKIFPEPTIIFAPR   2096
SemaH         ETEMPWQFDRLFRYEISHALNELELTHKSDFTIKVEVIAANGTRINEKIFPEPTIIFAPR   2097
LijaH2        ------------------------------------------------------------   1618
NunuH1        ------------------------------------------------------------   1672
MecrH1        ------------------------------------------------------------   1673
HacrH1        ------------------------------------------------------------   1675
HarufH1       ------------------------------------------------------------   1675
HadishaH1     ------------------------------------------------------------   1675
HadivH1       ------------------------------------------------------------   1590
HaasH1        ------------------------------------------------------------   1674
HarubH1       ------------------------------------------------------------   1675
```

```
CoasHaN         --------QLGATTTYLVRKNVESLNPLEGYHLADALIALKKDTSADGFQSIATFHAIPP      1745
HepoHaN         --------QHGSTSTYLVRKNVESLSPLEGYYLAEALIALKKDTSADGFQSIATFHAIPP      1744
HeluHaD         --------EDGDVKDIVVRKNVDALSPRETLSLIHALEALQADSSADGYQSIAAFHAVPP      1742
ApcaH1          --------ADGQVGNYLVRKNIASLSPQEELSLLKAMAALQADSSADGYQSIASFHAIPP      1745
LystH1          --------SSGEVTNYLVRKEINSLSPREVQSLYSAMEALQADSSADGWQSIASFHAIPP      1750
PhacH1          --------PSGEVSSYLVRKEINSLSPRETFSLYKAMESLQADSSADGWQSIAAFHAIPP      1749
PocaH1          --------ANGRTENRLVRKNAWALSPNELRSMMWAMRLLQEDSSPNGFQALASFHALPP      1732
MetuMH          --------QDGKTQRNMIRRNVLTLSLAERRSLVLAMRRLQEDHSADGFQALASFHALPE      1753
LisaH1          --------EHGHSERRLVRKDAWAISPAERRSLVIALRNLQADDSADGFQSLAAFHAVPP      1741
CrfoH1          --------DVGHVERRLVRKSVLSLSPAERRSLVLAMRSLQEDSSADGFQSLASFHALPP      1734
RaveH1          --------DEGHPISDLVRKSVWTLSPAERRSLVLAMKSLQADSSADGFQSLASFHAQPP      1747
OcvuHA          ---HSESDEKKLVRANLIRKNINRLSLEEIDSLMHALKRMQKDKSSDGFESIATFHALPP      1731
TopaH1          KERTEQVSTTKSVRGNLVRKNVDRLSLQEINSLIHALKRMQKDRSSDGFETIASFHALPP      2130
EuscH1          EERSFKSATNKNLYGNLVRKNVDRLSLQEINSLVHALKRMQKDQSADGFETIASFHAIPP      2157
SeofH1          EEHTVQSATHSNLYGNLVRKNVDRLSLQEVNSLVHALTRMQKDKSADGFETIASFHALPP      2156
SemaH           EEHTVHSATHSNLYGNLVRKNVDRLSLQEVNSLVHALKRMQKDKSADGFETIASFHALLS      2157
LijaH2          --------DSQHDSRELERKNINSLSPGDISSLMTAFRSMMADTSADGYQAIASFHAIPA      1670
NunuH1          --------HHG-GSDVTVRKEINTLTPLEVDSLMKAMRRLQEDSSPNGYQGIASFHAVPP      1723
MecrH1          --------DDGHTEPVMIRKDITQLDKRQQLSLVKALESMKADHSSDGFQAIASFHALPP      1725
HacrH1          --------DHDNTEEILIRKNINDLSPRERVSLVKALKGMQHDRSADGFQAIASFHALPP      1727
HarufH1         --------DHDNTEEILVRKNINDLSPRERVSLVKALKGMQNDRSADGFQAIASFHALPP      1727
HadishaH1       --------DHDNTEEILVRKNIIDLSPRERVSLVKALKGMQNDRSADGYQAIASFHALPP      1727
HadivH1         --------DHDNTEEILVRKNIIDLSPRERVSLVKALKGMQNDRSADGYQAIASFHALPP      1642
HaasH1          --------DHDHKEEILVRKNINDLSPREKVSLVKALEGMKNDRSADGYQAIASFHALPP      1726
HarubH1         --------DHDHKEEILVRKNINDLSPRERVSLVRALEGMKNDRSSDGYQAIASFHALPP      1727
                                  *:.   :   :   :  *:  :  * * :*:: :*:***

CoasHaN         LCPSPTASLRYACCIHGGTSFLQWHRLYTVQFEDALKRHGSPIGVPYWDWTRFSTELPRT      1805
HepoHaN         LCPSPTASRRYACCIHGGTSFLQWHRLYTVQFEDALKRHGSPIGVPYWDWTRFSKELPRT      1804
HeluHaD         LCPSPSASTRYACCLHGMSTFPQWHRLYTVQVEDALRRHGSVVGIPYWDWTRASQSLPHF      1802
ApcaH1          LCPSPSASKRYACCLHGMATFPQWHRLYTVQVEDALRRHGSLVGIPYWDWSRQTDHLPGL      1805
LystH1          LCPSPSAENRYACCLHGMATFPQWHRLYTVQVEDALKRHGALVGIPYWDWSRVSDSLPHF      1810
PhacH1          LCHPSASKRYACCLHGMATFPQWHRLYTFQVEDALRRHGSLVGIPYWDWSRLSSSLPHL       1809
PocaH1          LCPYPEAPVRYACCVHGMPTFPQWHRLYLVQFEDALRRHSALVGIPYWDSVEPSGFHPYM      1792
MetuMH          LCPYPEAAKRYACCVHGMASFPQWHRLYTVQFEEALRRHGSLVGIPYWDTSRPQKALPAF      1813
LisaH1          LCPSPEASERFACCVHGQATFPQWHRLYTVQFEDALRRHGALVGIPYLNSLEPVSMLPVL      1801
CrfoH1          LCPYPEATKRFACCIHGMATFPHWHRLYTVQFEDALRRHGALVGIPYWDTVVPNSRLPDF      1794
RaveH1          LCPYPEATKRFACCVHGMATFPAWHRLYTVQFEDALRRHGALVGIPYWDTVVPQSELPPF      1807
OcvuHA          MCPNPTAKHRYACCLHGMATFPQWHRLFVVQFEQALQRHGAKVGVPYWDWTYPMSEIPSF      1791
TopaH1          LCPNPTAKHRHACCLHGMATFPQWHRLYVVQFEHSLNRHGAIVGVPYWDWTYPMTEVPGL      2190
EuscH1          LCPNPTAKHRHACCLHGMATFPQWHRLYVVQFEQALHRHGATVGVPYWDWTYPMKEVPNL      2217
SeofH1          LCPNPTAKHRYACCLHGMATFPQWHRLHVVQFEQALHRHGATVGVPYWDWTYPMKEVPHL      2216
SemaH           LCPNPTAKHRYACCLHGMATFPQWHRLYVVQFEQALHRHGATVGVPYWDWTYPMKEVPHL      2217
LijaH2          LCPNPTAAQRYACCVHGMATFPQWHRIYVVQVEDALRQGSTVGLPYWDWLEPFTALPSI       1730
NunuH1          LCPSPAAANRFACCVHGMATFPQWHRLHVVQMEQALKDHGATVGLPYWDWTRPMTSLPDF      1783
MecrH1          LCPSPAASKRFACCVHGMATFPQWHRLYTVQFQDSLRKHGAVVGLPYWDWTLPRSELPEL      1785
HacrH1          LCPNPSAAHRFACCVHGMATFPQWHRLYTVQVQDALRRHGSLVGIPYWDWTKPVSELPEF      1787
HarufH1         LCPNPSAAHRFACCVHGMATFPQWHRLYTVQVQDALRRHGSLVGIPYWDWTKPVSELPEF      1787
HadishaH1       LCPNPSAANRFACCVHGMATFPQWHRLYTVQFQDALRRHGSLVGIPYWDWTKPVSELPKL      1787
HadivH1         LCPNPSAANRFACCVHGMATFPQWHRLYTVQFQDALRRHGSLVGIPYWDWTKPVSELPKL      1702
HaasH1          LCPSPSASKRYACCVHGMATFPQWHRLYTVQLQDALRRHGSLVGIPYWDWTKPVTELPKL      1786
HarubH1         LCPNPAAAHRYACCVHGMATFPQWHRLYTVQFQDALRRHGSFVGVPYWDWTKPATELPKL      1787
                :** * *  *.***:**  :*  ***:. .*.:.:*. :.: :*:** :        *

CoasHaN         FTFTNYSDPFTNVWTLNPFYKGRVEFEHV--DTERDVQEDKLFKSG-PHGWDTWLYTQVL      1862
HepoHaN         FTYANYSDPFTNLWTLNPFYSGRVEFEHV--DTERDVQEDKLFKRG-PHGWDTWLYTQVL      1861
HeluHaD         LSDNNYTDPYTKEVHDNPWHGASIDFEHS--HTERDIQSAELFKLG-PHGWDTWLFEQAL      1859
ApcaH1          LANPTYTDVYTGQTIDNPWYKAKIEFENS--VTERDVQGDYLFKQG-PHGFDTWLFNQAL      1862
LystH1          IDDENFVDPVTGDQKANPWKKARIEFENS--ETEREVVSDRLFKRG-PHGWDTWLFNQAL      1867
PhacH1          IDDESYIDPNSGETVANPWKKAKIAFENS--ETERDVVSDKLFKRG-PHGWDTWLFNQAL      1866
PocaH1          FTNKTYQVPVHKFQWNNPWESAAITFAGK--RTARDFQNDRLADS--DGGLGGWQWKQFV      1848
MetuMH          ATDEKFTDPVLNVEFDNPWLGADIEFENS--HTEREPNLARLGEEG-EHGYDTWLYEQYL      1870
LisaH1          MSDETWYDPLFNSDIPNPWAGADIEFKGA--KVARDVQSDRLAKKG-PHGYDTWSWKQYL      1858
CrfoH1          IAESVWDDPLFHANFSNPWAGADIEFDNS--AVVRDVNMDRISQKG-PKGYDTWSWKQYL      1851
RaveH1          FNDEVWDDPLFHANFPNPWQGAEIEFNHH--KVARDFDMDQLAKKG-PKGYDTWSWKQYI      1864
OcvuHA          LALEKYVNPYTGIEMFNPFNHGHISYISKETMTHRDISP-HMFE-KPELGKQTWLFNNII      1849
TopaH1          LTSEKYTDPFTGIETFNPFNHGHISFISPETMTTREVSE-HLFE-QPALGKQTWLFNNII      2248
EuscH1          LTSEKYVDPFTAVETFNPFNHGHISFISPETMTARDVSD-HLFE-QPGLGKQTWLFNNII      2275
SeofH1          LTSEKYTDPFTGVETFNPFNHGHLSFISPETMTTRDVSE-HLFE-QPALGKQTWLFNNIL      2274
SemaH           LTSEKYTDPFTGVETHNPFNHGHISFISPETMTTRDVNE-HLFE-QPALGKQTWLFNNII      2275
LijaH2          FTDDEFTDSVSLKTFKNPLKNVHIAFEDT--HVERDVIAEKLYE-QPKNGKQTWIYEQVM      1787
NunuH1          FNDGQYTDARTGVTFDNPFHHADINFEGAGVHTRRSPNEQRLFE-DPSLGYNTWLFENVI      1842
MecrH1          LTVSTIHDPETGRDIPNPFIGSKIEFEGENVHTKRDINRDRLFQ-GSTKTHHNWFIEQAL      1844
HacrH1          LSAETFHDPIHNLNVSNPFAKADIEFEGDGVHTERDIQSDFLFHSGDHEGYHNWFFETVL      1847
HarufH1         LSAETFHDPIHDLNVSNPFFKADIEFEGDGVHTERDIQSDFLFHSGDHEGYHNWFFETVL      1847
HadishaH1       LSAETFHDPIHNVNVSNPFFKADIEFEGDGVHTERDIQSDFLFHSGDHEGYHNWFFETVL      1847
```

```
HadivH1             LSAETFHDPIHNVNVSNPFFKADIEFEGDGVHTERDIQPDFLFHSGDHEGYHNWFFETVL        1762
HaasH1              LSSEIFYDPIHSINISNPFLQADIEFEGEGIHTQRHINADRLFHSGDHEGYHNWFFENVL        1846
HarubH1             LSSQTFHDPIHNINISNPFFQADIEFEGAGVHTERHIDADLLFHSGDHDGYHNWFFETVL        1847
                                    **     : :      . *      : .         *     :

CoasHaN             FALEQEDFCDFEVQLEIAHNAIHSWIGGSKEHSLAHLHYASYDPAFFIHHSNTDRLFAVW        1922
HepoHaN             FALEQEDFCDFEVQLEIAHNAIHSWIGGSKEHSLAHLHYASYDPAFFIHHSNTDRLFAVW        1921
HeluHaD             LALEQEDYCDFEIQFEITHNAIHSWVGGSKEHSLAHLHYASYDPAFYIHHSNTDRLWAIW        1919
ApcaH1              LALEQEDYCDFEIQFEITHNAVHSWLGGTKVHSMGHLHYASYDPAFYVHHSNTDRLWALW        1922
LystH1              LALEQEDYCNFEIQFEITHNAIHSWVGGSKEHSLGHLHYASYDPAFFVHHSNTDRLWAVW        1927
PhacH1              FALEQEDYCNFEIQFEITHNAIHSWLGGSKEHSLGHLHYASYDPAFFVHHSNTDRLWAVW        1926
PocaH1              FALEQEDYCDFEVQFEIAHNAIHAWVGGSEEYSMGHLHYASFDPVFLLHHSSMDRIYAMW        1908
MetuMH              LALEQDNYCDFEVQFEIAHNAIHAWVGGSKIHSMGHLHYASYDPIFLLHHSSTDRIFAVW        1930
LisaH1              YALEQEDYCDFEVQFEIAHNAIHAWLGGSETYSMAHLHYASYDPAFILHHSNTDRIFALW        1918
CrfoH1              YALEQENFCDFEVQFEIAHNALHAWMGGSEVHSMAHLHYASYDPVFILHHSNTDRIFALW        1911
RaveH1              FALEQEDYCDFEVQFEIAHNAIHAWVGGKEEYSMGHLHYASYDPIFILHHSNTDRIFALW        1924
OcvuHA              LALEQTDYCEFEIQFEVVHNAIHSWLGGKEMYSMNHLHYAAYDPAFYSHHANVDRLWVIW        1909
TopaH1              LALEQTDYCDFEVQFEIVHNSIHSWLGGKELYSLNHLHYAAYDPAFFLHHSNVDRLWVVW        2308
EuscH1              LALEQTDYCDFEVQFEIVHNAIHSWLGGKEIYSLNHLHYAAYDPAFYLHHSNVDRLWVIW        2335
SeofH1              LALEQTDYCDFEVQFEIVHNAIHSWLGGKEIYSLNHLHYAAYDPAFYLHHSNVDRLWVIW        2334
SemaH               LALEQTDYCDFEVQFEIVHNAIHSWLGGKELYSLNHLHYASYDPAFYLHHSNVDRLWVIW        2335
LijaH2              LALEQTNYCDFEVQFEIAHNSIHAWVGGSKKHSLGHLHYASYDPIFFFHHSNVDRLWAIW        1847
NunuH1              LALEQDNYCDFEVQFEVMHNAIHAWIGGGELRSMGHLHYASYDPIFYIHHSTTDRIFAMW        1902
MecrH1              LALEQTNYCDFEVQFEIMHNGVHTWVGGKEPYGIGHLHYASYDPLFYIHHSQTDRIWAIW        1904
HacrH1              LALEQEDYCDFEIQFEIAHNGIHTWIGGSAKYGMGHLHYASYDPIFYIHHSQTDRIWAIW        1907
HarufH1             FALEQEDYCDFEIQFEIAHNGIHTWIGGSAKYGMGHLHYASYDPIFYIHHSQTDRIWAIW        1907
HadishaH1           LALEQEDYCDFEIQFEIAHNGIHTWIGGSAKYGMGHLHYASYDPIFYIHHSQTDRIWAIW        1907
HadivH1             LALEQEDYCDFEIQFEIAHNGIHTWIGGSAKYGMGHLHYASYDPIFYIRHSQTDRIWAIW        1822
HaasH1              LALEQEDYCDFEIQFEIAHNGIHTWIGGDAVYGMGHLHYASYDPIFYIHHSQTDRIWAIW        1906
HarubH1             FALEQEDYCNFEIQFEIAHNGIHTWIGGNAVYGMGHLHYASYDPIFYIHHSQTDRIWAIW        1907
                     **** ::*:**:*:*: **.:*:*:**    .: *****::** *  :*:  **::.:*

CoasHaN             QKLQQYRGHDPNSANCALEQLREPLKPFSFGPPYNLNKVTQEYSRPQDTFAYKEHFHYKY        1982
HepoHaN             QKLQTYRGHDANSANCALEQLREPLKPFSFGPPYNLNKVTQDNSRPEDTFAYKEHFHYKY        1981
HeluHaD             QALQKHRGYSPNEANCALEHLKDSLKPFSFGPPYNLNKLTTKYSHPEDTFAYEDHFEYQY        1979
ApcaH1              QALQKYRGLNPNEANCALEQMRVPLKPFSFGAPYNLNPKTKEFSKPEDTFEYEEHFNYNY        1982
LystH1              QALQKYRGYDPNEANCALEQMKDPLKPFSFGPPYNLNPLTKEHSRPEDTFNYADHFHYRY        1987
PhacH1              QALQKYRGHDPNEANCALEQMKEPLKPFSFGAPYNLNPKTEEYSRPEDTFNYVDHFQYKY        1986
PocaH1              QELQRYRGLDPNEANCALQLVREPLKPFSFGSPYNLNPVTHQYSRPEDVFDYKARFNYQY        1968
MetuMH              QELQRLRGKDPNEANCALEIMHEPLKPFSFGSPYNLNENTQRYSRPEDIFDYQSHFHYEY        1990
LisaH1              QELQRYRGHDPNEINCALELMREPLKPFSFGAPYNLNPTTTKYSKPEDAFDYQAHFNYEY        1978
CrfoH1              QELQKYRGRDANEVNCALELMKEPLKPFSFGSPYNLNPTTTLYSKPEDAFDYKGHFKYEY        1971
RaveH1              QKLQKFRGHDPNNVNCALELMREPLKPFSFGSPYNLNDNTREHSKPEETLDYHTHFHYDY        1984
OcvuHA              QELQKLRGLPAYESNCAIEMMSKPLKPFSFGPPYNLNPHTYKYSTPSAVFKYKEHFHYEY        1969
TopaH1              QELQKFRGLPAYESNCAIELMSQPLKPFSFGAPYNLNPVTTKYSKPSDVFNYKQNFHYEY        2368
EuscH1              QELQKFRGLPAYESNCAIELMSQPLKPFSFGAPYNLNPMTTKYSKPADVFNYKDNFHYEY        2395
SeofH1              QELQKFRGFSAHESNCAIELMSQPLKPFSFGPPYNLNPMTTKYSKPSDVFNYKEHFHYEY        2394
SemaH               QELQKFRGFPAYESNCAIELMSQPLKPFSFGAPYNLNPMTTKYSKPSDVFNYKEHFHYEY        2395
LijaH2              QELQRYRGLGGSDANCALELMRVPLKPFSFGEPWNLNPLTKKYSKPADTFDYKNHFHYHY        1907
NunuH1              QALQNHRGLDGEHPNCALEVMKEKLKPFSFGKPYNLNPVTAEYSKPGDTFDFKNHFHYDF        1962
MecrH1              QSLQRFRGLSGSEANCAVNLMKTPLKPFSFGAPYNLNDHTHDFSKPEDTFDYQ-KFGYIY        1963
HacrH1              QELQKYRGLSGSDANCAIELMRTPLKPFSFGPPYNLNSHTQEFSKPEDTFDYK-KFGYRY        1966
HarufH1             QELQKYRGLSGSEANCAIELMRTPLKPFSFGPPYNLNSHTQEFSKPEDTFDYK-KFGYRY        1966
HadishaH1           QELQKYRGLSGSEANCAIELMRTPLKPFSFGAPYNLNSHTQEFSKPEDTFDYK-KFGYRY        1966
HadivH1             QELQKYRGLSGSEANCAIELMRTPLKPFSFGAPYNLNSHTQEFSKPEDTFDYK-KFGYRY        1881
HaasH1              QELQKYRGLSGSEANCAIELMRTPLKPFSFGPPYNLNRQTQEFSKPEDTFDYK-KFGYRY        1965
HarubH1             QELQKYRGLSGSEANCAIDHMRTPLKPFSFGPPYNLNHHTQEFSKPEDTFDYK-KFGYRY        1966
                    * **  **      ***:: :   ******* *:***  *   * *   : :  .* * :

CoasHaN             DALEFVGMNIPTLDAYIKERQEHDRVFAGFLLKGIGQSATVEFEICSDA-TGDCFDGGEF        2041
HepoHaN             DALEFVGMNIPTLDAYIKERQEHDRVFAGFLLKGIGQSASVKFEICIDA-TGVCFDGGEF        2040
HeluHaD             DSLEFVGMNIPALDAYIKERQEHDRVFAGFLLHGLGTSASVEFTICDA--FKKCFEGGHF        2037
ApcaH1              DHLEFVGLDIPALDGFIKEKQEEDRVFAGFLLKGFGSSAFVNFDIVSN---GKSFEGGYF        2039
LystH1              DNLEFVGLSIPQLDAFIKERHEHDRVFAGFLLKGFKKSALVDFDICAN---GNCFPGGYF        2044
PhacH1              DSLEFVGLSIPQLDAFIKEKQEHDRVFAGFLLKGFGESGSLNFNICPK--AKACFDGGYF        2044
PocaH1              DTLELLGMDVPRLQGYINKQKEKPRVFAGFLLHSLGTSAHVTFSVCSGEEYQECTLAGDF        2028
MetuMH              DSLEVLGMSPAQLEDAIHKRQEKDRVFAGFLLKGIGTSAHVEFTICNEA--GVCTTAGDF        2048
LisaH1              DKLELQGLDMSRIQDYINERKEKDRVFAGFLLHGFGTSAHVEFAVCNEE--GTCTPAGDF        2036
CrfoH1              DTLELQGLDVQRLQDYINKEKEHDRVFAGFLLSGIGQSAHASFSVCKAN--GECTAAGDF        2029
RaveH1              DSLELQGMDVHRLQDYINQQKEEDRVFAGFLLQGIGTSAHVAFSVCKDG--GECTSAGFF        2042
OcvuHA              DMLEMNGMSVAQLEAYIRTQKQRERMFAGFLLKGIGQSAYATFQVCDKK--NSCHDGSHF        2027
TopaH1              DMLEMNGMSIAQLESYIRQERQKDRVFAGFLLEGFGSSAYATFQVCPDV--GDCYEGSHF        2426
EuscH1              DMLEMNGMSIAQLESYIRQEKQKDRVFVGFLLHGFGSSAYATFDICNDA--GECREGSHF        2453
SeofH1              DMLEMNGMSIAQLESYIRQEKQKDRVFAGFLLEGFGSSAYATFQICSDA--GECHDGSHF        2452
SemaH               DMLEMNGMSIAQLESYIRQEKQKDRVFAGFLLEGFGSSAYATFQICNDA--GECHDGSHF        2453
LijaH2              EHLEVGGLNVPKLDRYLRERQEHDRVFAGFLLHGFGSSGTVKFNVCDPN--GKCTEGGSF        1965
NunuH1              DKLEIGGMSAAQLDTFIQERKERDRVFAGFLLAGFKMSATVKFQVCKED--GTCQAGGSF        2020
```

```
MecrH1          DTLEFAGWSIRGIDHIVRNRQEHSRVFAGFLLEGFGTSATVDFQVCRTA--GDCEDAGYF          2021
HacrH1          DSLELEGRSVAHIDELIKERQEHDRTFAGFLLKGFGTSATVTLKICRDD--HTCQNAGYF          2024
HarufH1         DNLELEGRSVAHIDELIQERQEHDRTFAGFLLKGFGTSATVTLKVCRDD--HTCQNAGYF          2024
HadishaH1       DNLELEGRSVAHIDELIKERQEHDRTFAGFLLKGFGTSATVTLKICRDD--YTCENAGYF          2024
HadivH1         DNLELEGRSVAHIDELIKERQEHDGTFAGFLLKGFGTSATVTLKICRDD--YTCENAGYF          1939
HaasH1          DNLELEGRSISHIDELIKERQEQDRTFAGFLLKGFGTSATVSFDICTVN--HTCKEAGYF          2023
HarubH1         DNLELEGRSIAHLDELIKERQEQDRTFAGFLLKGFGTSATVSFEICRVD--HTCEKAGYF          2024
                : **. * .   ::  :. .::.   *.**** .:  *.   : :        .  .. *

CoasHaN         SILGGSAEMPWQFNRLYKYEITDQLESHHVHYDDEYHLTVHLKS-INGTELDSHLVHEPT          2100
HepoHaN         SILGGSAEMPWQFNRLYKYEITDQLESHHVHYDDEYHLTVHLKS-INGTELDSHLIHEPT          2099
HeluHaD         TVLGGELEKPWQFDRLYRYPITDVLKENHIHYDDDYHFHIHIKA-LNGTELDSKLIPEPS          2096
ApcaH1          TVLGGSAEMPWQFDRLFKYEITDQLIAANLRFDDNYSFNISVRL-PDGTILDSSLIPTPS          2098
LystH1          TVLGGSAEMPWQFDRLFKYEITDQLNEHKLRFDDEYTFQIKIRA-PDGTELSSDLIGTPS          2103
PhacH1          TILGGSAEMPWQFDRLFKYEITEQLEEHGLRFDDEYTFEIHIKA-PNGSELASSLISAPS          2103
PocaH1          NVLGGSAEMPWRFDRLYRYEITDVLKTKGLKVDDMFQIKVVITA-QNGTVLDSNSLPQPT          2087
MetuMH          DILGGSAEMPWSFDRAYKYDITNVLEKKGLEATDAFKVKVAITA-QDGTALDSSLLPEPS          2107
LisaH1          NVLGGSAEMPWRFDRLYKYEITDVLESKGLDVRDTFHIEVMVTA-ANGTVLDSHILPDAS          2095
CrfoH1          DILGGSAEMPWRFDRLYKYEITDVLEKKGLDVHDSFNITVSLTA-LDGSALSSSLLPTPS          2088
RaveH1          DVLGGALETPWRFDRLYKYEISDVLKEKGLEVRDAFHIELTITA-SNGTALPSSLIPEPS          2101
OcvuHA          SILGGSAEMSWAFDRLYVMEITDILHDMGLKFDSHFHIHHHIKT-ANGTDLPSDLIPQAT          2086
TopaH1          SVLGGSTEMPWAFDRLYKMEITDILQAMBLKFDSHFTIKTKIVA-HNGTELPESLLPEAT          2485
EuscH1          SVLGGSTEMPWSFDRLYRIEITDILKDMGLEFDSHFTIKVQLAA-HNGTELNH-VLSEPT          2511
SeofH1          SVLGGSTEMPWAFDRLYRIEITDILKDMGLQFDSHFTIKVNLLA-HNGTELTG-VLTEPT          2510
SemaH           SVLGGSTEMPWAFDRLYRTEITDILKDMGLQFDSHFIIKVNLLA-HNGTELTG-VLTEPT          2511
LijaH2          HVLGGSMEMPWAFDRQYRYDITSAVEALGARKDDALSISVSIVA-TNGTEVPSSILKTPN          2024
NunuH1          EVLGGTLEMPWRFDRLYKYEITDVLDKMDIRYDDPFHFEWTVTPTMPGDTIDSNPISEPS          2080
MecrH1          TVLGGEKEMPWAFDRLYKYDITETLDKMNLRHDEIFQIEVTITS-YDGTVLDSGLIPTPS          2080
HacrH1          TVLGGSAEMPWAFDRLYKYDITKTLDHMNLRHEDTFYINRTVTA-YDGTVLPGDLIPPAS          2083
HarufH1         TVLGGSAEMPWAFDRLYKYDITKTLDHMNLRHEDTFYINGTVTA-YDGTVLPGDLIPPAS          2083
HadishaH1       TVLGGSAEMPWAFDRLYKYDITKTLDHMNLRHEDTFYINGTVTA-YDGTVLPGDLIPPAS          2083
HadivH1         TVLGGSAEMPWAFDRLYKYDITKTLDHMNLRHEDTFYINGTVTA-YDGTVLPGDLIPPAS          1998
HaasH1          TILGGSAEMPWVFDRLYKYDITKTLNDMHLRHEDTFSVRVNVTS-YDGTVLSGDLIQTPS          2082
HarubH1         TVLGGSAEMPWVFDRLYKYDITKTLHDMHLRHEDTFSVKVTVTS-YNGTVLSGDLIQTPS          2083
                 :***  *  * *:* :   *:. :        .   .   :     *  :    :   .

CoasHaN         VIYVPAKHDVTLKKVTVNHIRRNLDDVEDRDTQSIQTALRKLLADTGPDGWASLASFHGA          2160
HepoHaN         VIYVPAKHDVTLKKVTVNHIRRNLDEVEERDTQSIQRALRDLLADTGPDGWASLASFHGA          2159
HeluHaD         VLFVAAHEDVHHVEATPNNIRRNLNTLEERDIQSLQAALRDLQRDTTNDGWANLASFHGA          2156
ApcaH1          VLFKSAQHDFKREHVAPNHVRRNLDTLEERDLQSLKAALRDLQHDNSNDGWASLASFHGS          2158
LystH1          VILEPFKRDYDLEKVTPNFVRHDLSTLTERDLQGLKSALRDLQLDESADGWASLASFHGA          2163
PhacH1          VILDPAHKVTDLEKVTHNYVRRDLNSLTERDVQSLKAALRDLQLDTTNDGWASLASMHGA          2163
PocaH1          VIFMPKIRERDLQDAPPSRVRRDLRDLKEVDIQNLKAAMASFQRDKGGNGWEAITAFHGV          2147
MetuMH          IILQHATRDQGQYELAPNRVRRSLSDLTERDVMSLKSALHDLQEDDSATGWQSLAAYHGV          2167
LisaH1          VIYDPKSRDSQLHEVAPNHVRHDLSHLSERDVMSLKAAMLDMQRDKGTSGYQNIAAFHGA          2155
CrfoH1          VIFEPKSRTTELHEVAPNRIRHDLTHLSERDIRSLKSSIRDLQLDDSNDGYQNIASFHGA          2148
RaveH1          VIYAPKSHDTELHEVAPNRVRHDLADLTDRDIKNLKSAIRDLQNDISKDGYQHIATFHGA          2161
OcvuHA          IIRIPAMRLEKQRFIPHNRIRKNINSLEERDVQNIMSALTRMKEDESNFGFQTVASYHGS          2146
TopaH1          IVRIPPSAQNLEVAIPLNRIRRNINSLESRDVQNLMSALKRLKEDESDFGFQTIAGYHGS          2545
EuscH1          IVRIPPEKEHIEVAIPLNKIRRQIESLEERDIQNLMSALRRLKEDDSDFGFQTIAGYHGS          2571
SeofH1          VVRIPPEREHIEVAIPLNKIRRNIQTLDERDIQNLMSSLRRLKEDESDFGFQTIASYHGS          2570
SemaH           VVRIPPEREHVEVAIPLNKIRRNIQTLDERDIQNLMSSLRRLKEDESDFGFQTIASYHGS          2571
LijaH2          VFFVPGKDTVRNAQTPPNRVRQNINNVNERDIQSIMAALAELKEDESADGFAAIAAFHGS          2084
NunuH1          VIFVPAIVDHNEDKFAHNQVRRNINELDEADVRSLSSALTDLMADEDKDGFQAIARFHGM          2140
MecrH1          IIYDPAHHDISSHHLSLNKVRHDLSTLSERDIGSLKYALSSLQADTSADGFAAIASFHGL          2140
HacrH1          IIFVPGRHTLNSRKHAPNKIRHELSSLSSRDVASLKAALTSLQKDDGPDGYQAIAAFHGL          2143
HarufH1         IIFVPGRHHLNSRKHAPNKIRHELSSLSSRDVASLKAALTSLQKDDGPDGYQAIAAFHGL          2143
HadishaH1       IIFVPGRHNLNSRKHTPNKIRHELTSLSSRDVASLKAALTSLQKDDGPNGYQAIAAFHGL          2143
HadivH1         IIFVPGRHNLNSRKHTPNKIRHELTSLSSRDVASLKAALTSLQKDDGPNGYQAIAAFHGL          2058
HaasH1          IIFVPGRHKLNSRKHAPNKIRHEISSLSSRDVASLKAALTSLQHDDGPDGYQSIAAFHGL          2142
HarubH1         IIFVPGRHKLNSRKHAPNKIRHELSSLSSRDIASLKAALTSLQHDSGTDGYQATAAFHGV          2143
                :.               . :*:.:  : . *  .:  ::  :  *    *:   :  **

CoasHaN         PARCPDPDHPTVACCQHGMFTFLHWHRLFTLQVEQAIQKHGSSIAIPYWDWTQPLKKLPD          2220
HepoHaN         PARCPDPDHPTVACCQHGMPTFLHWHRLFTLQVEQAIQKHGSAIAIPYWDWTQPIKKLPD          2219
HeluHaD         PARCPDPKHPTVACCVHGMPTFPHWHRLFALQIEQALHRHGSSIALPYWDWTLAIDDLPS          2216
ApcaH1          PAKCSTPANESVACCIHGMPTFPHWHRLFTLQVEQALRRHGSAIAIPYWDWTLPITDLPE          2218
LystH1          PSMCENESGAKVACCIHGMPTFPHWHRLFTVQVERALQRHGSAVAIPYWDWTKPITALPE          2223
PhacH1          PARCKDDAGKDVACCIHGMPTFPHWHRLFTLQVEQALQRHGSAIAIPYWDWTKPIKALPE          2223
PocaH1          PARCPSPQKPEKACCIHGMPTFPHWHRLYTLQVDMSVVRKGSSVALPYWDWTLPTDPLPS          2207
MetuMH          PALCPSPEEAKYACCIHGMPTFPHWHRLYVLAVEHSLIKHGSSVAIPYWDWTNPLDKLPE          2227
LisaH1          PAMCPTPEAAEYACCVHGMPTFPHWHRLYTVEMEDALIKHGSSVALPYWDWTLPIEHLPD          2215
CrfoH1          PALCPSPEAAEYACCVHGMPTFPHWHRLYTVEMEDAMVRHGSSVALPYWDWTMPITQLPD          2208
RaveH1          PALCPDPNAPTHACCLHGNPTFPHWHRLYAVEMEDALIRHGSGVALPYWDWMKPITELPE          2221
OcvuHA          T-MCPDPENAKYACCQHGMATFPHWHRIYALQFEEALRRHGSNVAIPYWDWTTHSEKLPS          2205
TopaH1          L-MCPTPEAPEYACCLHGMPTFMHWHRVYLLHFEESMRRHGASVAVPYWDWTMPSDNLPS          2604
EuscH1          K-NCPSPDDAQYACCLHGMPTFPHWHRVYLLHFEDAMRRHGANVAVPYWDWTLPISSLPH          2630
```

```
SeofH1          L-LCPTPEAPEYACCLHGMATFPHWHRVYLLHFEDAMRRHGANVAVPYWDWTLPISGLPS    2629
SemaH           L-LCPTPEAPEYACCLHGMATFPHWHRVYLLHFEDAMRRHGANVAVPYCDWTLPISGLPS    2630
LijaH2          PAKCVRDSGEPVACCVHGMAAFPHWHRLYTVQFEDALRRHGSSVALPYWDWTVD-ASLPK    2143
NunuH1          PVQCGD-----VACCHHGMPTFPHWHRLITVQFEMALQRHGSSSGLPYWDWTEKMDALPH    2195
MecrH1          PAKCNDSHNNEVACCIHGMPTFPHWHRLYTLQFEQALRRHGSSVAVPYWDWTKPIHNIPH    2200
HacrH1          PAQCHDTSGHQIACCIHGMATFPHWHRLYTLQLEQALAKHGSSVAVPYWDWTRPITELPH    2203
HarufH1         PAQCHDTSGHQIACCIHGMATFPHWHRLYTLQLEQALAKHGSSVAVPYWDWTRPITELPH    2203
HadishaH1       PAQCHDTSGHQIACCIHGMATFPHWHRLYTLQLEWALAKHGSSVAVPYWDWTRPITELPH    2203
HadivH1         PAQCHDTSGHQIACCIHGMATFPHWHRLYTLQLEWALAKHGSSVAVPYWDWTRPITELPH    2118
HaasH1          PAQCHEASGREIACCIHGMATFPHWHRLYTLQLEQALSKHGSSVAVPYWDWTKPITELPH    2202
HarubH1         PAQCHDASGREIACCIHGMATFPHWHRLYTLQLEQALSRHGSSVAVPYWDWTKPITELPH    2203
                   *        *** **  :* ****:   : .: :: ::*:  .:** **      :*

CoasHaN         IFTRVNYYDAWSDQVLENPFAHGSIPSEKAQTVRDVQPE--LFETTEDGKHSTLFPLLLE    2278
HepoHaN         IFTKVNYYDAWSDQVLENPFAHGSIPSEKAQTVRDVQPE--LFETTKDGKHSTLFPLLLE    2277
HeluHaD         TFTKEDYYDVWRDEVVPNPFAHGYVASEDTYTVRDIQET--LHDRHVDGKHSFLFYGVLE    2274
ApcaH1          IFTSQNYYYVWRDVVVNNPFARGYLPTEDVYTVRDIMPE--VRNKNQAGDHSAIFDLALS    2276
LystH1          IFTKEDFYDAWRDEVVDNPFARGFIPTENTYTVRDIRPE--LFQTSKDGSHSDLFELVLS    2281
PhacH1          IFTDEDYYDIWRDEVVPNPFARGYVPSEEVYTVRDVRQE--LFKTSKDGSHSDLFDLVLS    2281
PocaH1          LFTEQTFYDAWKDEVLENPFARGFIKEISGYTVRDPQPE--LLKLSADGEHSVLFDEVLL    2265
MetuMH          LFTKQTYYDAWRDEVYDNPFARGYVKGDEAYTVRDPQPE--LKERSRDGKHSVLFDQVLL    2285
LisaH1          LFTKETYYDAWRDEVMDNPFARGYVSSVGGFTVRDPQPE--LTKLSRDGKHSVLFDEVLL    2273
CrfoH1          LFTSETYYDAWRDEVVANPFARAYIKVAGGYTVRDPQAQ--LKQLSRDGQHSALFDLVLL    2266
RaveH1          LFTSESYYDPWRDEVVPNPFVRAYIKAANGYTVRDPRPA--LKKLSHDGKHSILFDEVLL    2279
OcvuHA          VLSRSDYYDAWNDVVIENPFLRGYIKSEDTYTVRDVQQE--LFELAKGGETSLLYDQVLL    2263
TopaH1          LLGDADYYDAWTDSVIENPFLRGHIKYEDTYTVREIQPE--LFALAEGQKESTLFKDVML    2662
EuscH1          LLADADYYDAWSDSVIENPFLRGFIKSEDTFTVRDIQPD--LFKIAEGGKVSVLYKQVML    2688
SeofH1          LLADADYYDVWSDSVIENPFLRGFIKQEDTFTVRDIQPE--LYKFAEGGKVSVLYKQVML    2687
SemaH           LLADADYYDVWSDSVIENPFLRGFIKQEDTFTVRDIQPE--LYKFAEGGKVSVLYKQVML    2688
LijaH2          LATRADYYDAWTDTVVENPFLRGRIEHEDTYTARNVQPE--LTEVGPDGKN-ALYESMLL    2200
NunuH1          LLTEPEYYDAWTDKVVPNPFARGYIESEDTYTVRDVRNEFKLFDNAPDAKHSILFNSVMY    2255
MecrH1          LFTDKEYYDVWRNKVMPNPFARGYVPSHDTYTVRDVQEG--LFHLTSTGEHSALLNQALL    2258
HacrH1          ILTDGEYYDFWHDAVMPNPFSRGHVNFENTFTVRNVQED--LFKLSPIGKHSVLFDQALL    2261
HarufH1         ILTDGEYYDFWQDAVMPNPFSRGHVNFENTFTVRNVQEA--LFKLSPIGKHSVLFDQALL    2261
HadishaH1       ILTDGEYYDFWQDAVMPNPFSRGHVKFENTFTVRNVQEA--LFKLSPIGKHSVLFDQALL    2261
HadivH1         ILTDGEYYDFWQDAVMPNPFSRGHVKFENTFTVRNVRKA--LFKLSPIGKHSVLFDQALL    2176
HaasH1          ILTDREYYDVWQNAVMPNPFARGYVKFKDAFTVRNVQEG--LFKMSALGKHSLLFDQALL    2260
HarubH1         LLTDGEYYDVWQNAVITNPFARGYVKFKDAFTVRNVQEG--LFKMSTFGKHSLLFDQALL    2261
                      :*  * : *  *** :. :      *.*:      :       .   :    :

CoasHaN         ALEQTSYCDFAVQFEVLHNAIHYLVGGHQKYSLSSLEYSAYDPIFFIHHSFSDKLWVVWQ    2338
HepoHaN         ALEQTSYCDFAVQFEVLHNAIHYLVGGHQKYSLSSLEYSAYDPIFFIHHSFSDKLWVVWQ    2337
HeluHaD         VLEQTDYCDFEVHFEVVHNAIHYLIGGHQTYSLSSLEYSAYDPIFFIHHSFTDKLWAVWQ    2334
ApcaH1          AMEQTDYCDFEVQLEVMHNAIHFLVGGLQTYSLSSLEYSAYDPLFFIHHSFTDKIWAVWQ    2336
LystH1          ALEQTDYCDFEVQFEVMHNAIHYLVGGLQTYSLSSLEYSAYDPIFFIHHSFVDKIWVVWQ    2341
PhacH1          ALEQTNFCDFEVQFEVMHNAIHYLVGGHQKYSLSSLEYSAYDPIFFIHHAFVDKIWVVWQ    2341
PocaH1          VLEQTDYCDFEVQFEVVHNAIHYLVGGRQSYSLSSLHYASYDPLFFIHHSFVDKIWAVWQ    2325
MetuMH          ALEQTDYCDFEVQYEVTHNAIHYLVGGPQTFSLSSLHYSSYDPIFFVHHSFVDKIWAVWQ    2345
LisaH1          ALEQTDFCDFEVQYEVLHNAIHYLVGGRQTFALSSLEYSSYDPIFFVHHSFVDKIWVIWQ    2333
CrfoH1          VLEQTDYCDFEVQFEVVHNAIHYLVGGRQLYSMSSLEYTSYDPIFFVHHSFVDKIWAVWQ    2326
RaveH1          ALEQTDYCDFEVQFEVTHNAIHYLVGGRHLYSLSSLEYSSYDPIFFVHHSFVDKIWAVWQ    2339
OcvuHA          MLEQEDFCDFEVQFEVVHNSIHYLLGGHQKYAMSSL---------------VDRIWAIWQ    2308
TopaH1          MFEQEDYCDFEVQAEVIHNSIHYLIGGHQKYAMSSLMFSSFDPIFYVHHSMVDRLWAIWQ    2722
EuscH1          MFEQEDYCDFEVQLEVIHNTIHYLLGGHQKYAMSSLMYSSFDPIFYIHHSMVDRLWAIWQ    2748
SeofH1          MFEQEDYCDFEIQLEVIHNSIHYLLGGYQKYAMSSLVFSSYDPAFYIHHSMIDRLWAIWQ    2747
SemaH           MFEQEDYCDFEIQLEVIHNSIHYLLGGHQKYAMSSLVFSSYDPAFYIHHSMIDRLWAIWQ    2748
LijaH2          AFEQEDYCDFEVQFEVSHNAIHYLVGGRHEYALSSLSYTSYDPIFFLHHSQVDRLWAIWQ    2260
NunuH1          ALEQEDFCDFEVQFEVIHNAIHFLIGGFQTYALSSLHYSSYDPLFYIHHANVDRIWAVWQ    2315
MecrH1          ALEQHDYCDFAVQFEVMHNTIHYLVGGPQVYSLSSLHYASYDPIFFIHHSFVDKVWAVWQ    2318
HacrH1          ALEQTDYCDFEVQFEVMHNTIHYLIGGRQTYAFSSLHYASYDPIFFIHHSFVDKIWAIWQ    2321
HarufH1         ALEQTDYCDFEVQFEVMHNTIHYLIGGRQTYAFSSLHYASYDPVFFIHHSFVDKIWAIWQ    2321
HadishaH1       ALEQTDYCDFEVQFEVMHNTIHYLIGGRQTYAFSSLHYASYDPIFFIHHSFVDKIWAIWQ    2321
HadivH1         ALEQTDYCDFEVQFEVMHNTIHYLIGGRQTYAFSSLHYASYDPIFFIHHSFVDKIWAIWE    2236
HaasH1          ALEQTDYCDFEVQFEVMHNTIHYLVGGRQTYAFSSLEYSSYDPIFFIHHSFVDKIWAVWQ    2320
HarubH1         ALEQTDYCDFEVQFEVMHNTIHYLVGGRQTYAFSSLEYSSYDPIFFIHHSFVDKIWAVWQ    2321
                 :** .:*** :: ** **:**:*:** : :::***                *::*.:*:

CoasHaN         ELQQRRHLPYHTADCAVNAMSEPMKPFSF-ETFNTNKFTREHSVPNTVFDHENLGYTYDN    2397
HepoHaN         ELQQRRHLPYHTADCAVSAMSEPMKPFSF-ETFNTNKFTRDHAGPNSLFDHENLGYTYDH    2396
HeluHaD         ELQKRRHLPYNRADCALNYVNEPLKPFSF-EGFNLNKFTREHAVPNTLFNNEDLGYAYDN    2393
ApcaH1          ELQTRRHLPANKADCALNYMSQPMRPFFF-EGFNLNKFTMDHAVPNTVFDYEHLGYSYDD    2395
LystH1          ELQQRRHLPSNTANCALNYLAEPMKPFNL-D-LNINAFTKKHAVPRDVFNHEELGYHYDN    2399
PhacH1          ELQQRRHLPSNRADCALNYMNEPMKPFNI-EGFNINSFTKKHAVPNTVFNYEHLGYQYDN    2400
PocaH1          ELQKRRHLPHDRADCAVNFMAEPMAPFNN-PKVNFNPRTRAYAVPQTVFDYEGLEYTYDN    2384
MetuMH          ALQRKRGKPSERADCAVNYMYDDMHPFDS-AKLDPDPTIRSHANPSTVFNIYDLGYKYDD    2404
LisaH1          EMQKHRGLSYDRADCAVNYMNEVLHPFDW-AQFNTDVRTRAHSLPQSVFDYEDLGYHYDN    2392
CrfoH1          ELQKRRGMNYDRADCAVNFMNERMHPFDW-EALNPDVRTREHSLPQSVWKYEDLGYHYDN    2385
```

```
RaveH1          ELQKRRGLTYDRADCAVNFMQHKMHPFDW-DDLNPDVRTREHAHPQTVFDYEDLGYHYDD        2398
OcvuHA          ELQEYRKMPFDKAYCALDQMSLAMKPFT--WESNPNLATRALATPSMLFDYKRFGYKYDN        2366
TopaH1          ELQKHRKLPHDKAYCALDQMAFPMKPFI--WESNPNPTTRAVSTPSKLFDYKSLGYDYDH        2780
EuscH1          ELQHFRKLPDNKAFCALDQMAFAMKPFF--WESNPNPHTRSVAVPAKLFDYKSLGYIYDD        2806
SeofH1          ELQHFRKLPDKKAFCALDQMSFQMKPFH--WDSNPNAHTRSSSTPAKLFDYKGLGYIYDD        2805
SemaH           ELQHFRKLPDKKAFCALDQMSFQMKPFH--WDSNPNPHTRSSSTPAKLFDYKGLGYIYDD        2806
LijaH2          ALQKHRKRPYLKAYCAAAQMTMPMKPFKFNETFNMNSVTRSHARPDSVFNYENLGYTYDD        2320
NunuH1          ELQKARGMPHNSANCALNLMSEPMHPFD--MGININKVTKRHATPSTVFDFEDFGYTYDS        2373
MecrH1          ALQEKRGLPSDRADCAVSLMTQNMRPFH--YEINHNQFTKKHAVPNDVFKYELLGYRYDN        2376
HacrH1          ELQSRRHLQFNGADCAVSLMGKAMRPFN--KDFNHNPFTKRHAVPNTLFDYEDLGYNYDN        2379
HarufH1         ELQSRRHLQFNGADCAVSLMSKAMRPFN--KDFNHNPFTKRHAVPNTLFDYEDLGYNYDN        2379
HadishaH1       ELQSRRHLQFNAADCAVSLMSKAMRPFN--KDFNHNPFTKRHAVPNTLFDYEDLGYNYDN        2379
HadivH1         ELQSRRHLQFNGADCAVSLMSKAMRPFN--KDFNHNPFTKKHAVPNTLFDYEDLGYNYDN        2294
HaasH1          ELQSRRHLQFKAADCAVGLMSKAMRPFN--KDFNHNSFTKKHAVPNTVFDYEDLGYNYDN        2378
HarubH1         ELQSRRHLQYKAADCAVGLMSKAMRPFN--KDFNHNSFTKKHAVPNTVFDYEDLGYNYDN        2379
                 :*  *      * **   :   : **      : :      : *  ::.   : * **

CoasHaN         LNVGGYSLDQLEELIHDNQQHARVFAGFLLHGIKTSGSVKIKLCQG---DKCTSAGQFNL        2454
HepoHaN         LKVGGYDLGQLEELIHDNQQHARVFAGFLLHGIKTSGSVKIKLCQG---DKCTSAGQFNL        2453
HeluHaD         FNIGGYDLDELEKLIHDRQIKPRIFAGFLLKGIKTSGSVNLKVCKF---EECTPAGFFNL        2450
ApcaH1          LSIGGYDLDGLEQVIADRQSKARVFAGFLLKGVKTSGSVVINICLRN--NVCNYAGRFNV        2453
LystH1          LNIGGYNLDQLEQLIAQHQSRPRVFAGFLLKGIKTSGSVVLNVCKG---SQCTYAGRFNL        2456
PhacH1          LEIGGYNIEQLEALIAQRQSRPRVFAGFLLKGVKTSGSVVLQICNSR--KQCSYAGRFNL        2458
PocaH1          LNIGDKTPDELEKLINKQRSRPRVFAGFLLHGIGTSADVVISVCVH---SNCVRAGAFFI        2441
MetuMH          YQIGGKNLDELEALIKDNQAHPRVFAGFHLKGIGSSADVVFKICKDKSGGQCTRAGSIFI        2464
LisaH1          LELGGKTLEELEELIHDQQSHPRVFAGFHLHAIGTSADVIFEVCKTD--DECYRAGGFFI        2450
CrfoH1          YQIGGKSIEELEELIKKQQSHPRVFAGFQLHGLGTSADVELSVCKSQ--NSCVSAGVIFI        2443
RaveH1          FKLGGNTLEELEEMIHEKKSHPRVFAGFHLHNIGTSADVVFHICTTE--SRCTRAGGLFI        2456
OcvuHA          LEFHGMDTEHLEIEIQKLKQKDRVFATFLLHGIKTSADVHLKLCDD---KSCSAAGVVFI        2423
TopaH1          LNFHGMSIGQLEALIQKQKKADRVFAGFLLHGIKISADVHLKICIE---ADCQEAGVIFV        2837
EuscH1          LTFHGMSIAQLDAAIQNQKAKDRVFAGFLLHGIKTSADVHLKVCNE---DICKDAGVLFI        2863
SeofH1          LTFHGMSIAQLEAAIQRQKEDDRVFAGFLLHGIKTSADMHLKVCNE---ADCKEAGTIFI        2862
SemaH           LTFHGMSIAQLEAAIQRQKEDDRVFAGFLLHGIKTSADMHLKVCNE---ADCKEAGTIFI        2863
LijaH2          LKFDGHSIEELSEIVERQTQSDRVFANFLLHGIGTSADVKFSVCKTD--NTCERAGLFFI        2378
NunuH1          FKIGGLSLSEIQEEIDNRRNHDRVFAGFLLSGIKTSALVHFHLCKSD--DDCIKAGEFGV        2431
MecrH1          LEIGGMNLHEIEKEIKDKQHHVRVFAGFLLHGIRTSADVQFQICKTS--EDCHHGGQIFV        2434
HacrH1          LEISGLNLKEIEALIAKRKSHARVFAGFLLFGIGTSADVHLDICKTS--DDCHHAGVLFI        2437
HarufH1         LEISGLNLKEIEALIAKRKSHARVFAGFLLFGIGTSADVHLDICKTS--EDCHHAGVLFI        2437
HadishaH1       LEISGLNLKEIEALIAKRKSHARVFAGFLLFGIGTSADIHLDICKTS--DDCHHAGVLFI        2437
HadivH1         LEISGLNLKEIEALIAKRKSHARVFAGFLLFGIGTSADIHLDICKTS--DGCHHAGVLFI        2352
HaasH1          LELSGLNLNEIEALITKRKSHARVFAGFLLFGIGTSADVHLDICKTS--ENCHDAGVIFI        2436
HarubH1         LELSGLNLNEIEALISKRKSHARVFAGFLLFGLGTSADIHLDICKTS--ENCHHAGVIFI        2437
                  . .     :.  :       *:** * *  :  *. : : :*       *  .* . :

CoasHaN         LGGPLESPWAYNRLYKRDITQYLAALNLHPEDVFDPTLQVHLEVEVRDVQDHVLEAQNVL        2514
HepoHaN         LGGPLESPWAYNRLYKRDITQYLEDLNLHPEDVFDPILQVHLEVEVRDVQDHLLVAQDVL        2513
HeluHaD         LGGPLEMPWAFDRLFKKDITWTIARIGLNPDDIHKTDSGFKLEVQAFNVEGTALPLSQAI        2510
ApcaH1          LGGPIEMAWAFDRLFTYDITSALEQSGINPEDVFDAEALFTLDIKVFDVEGHALPVSSVL        2513
LystH1          LGGPSEMPWAFDRLYKKDITWALEAADIKPESVFDADSLFTLDVKVFDVEGHAINVKSVF        2516
PhacH1          LGGPTEMEWAFDRLYKKDITWALEEAGISPESVFDAENAFTLDVKVYNVDGESINVKSVF        2518
PocaH1          LGGALEMQWSFDRLYRHDITQALLAVSVRPQDLFTEHTPFTLDYSITAVNGTQLPRS-LM        2500
MetuMH          LGGEKEMPWEFDRLYKYDITDALKDNGIQPEDVFDAEAPFYLKYEVTAVNGSTLPKS-VI        2523
LisaH1          LGGHLEMPWTFDRLYKYDITDTLHDADISPEDVFNAQTPFTIKYSIHAVNGSALPLS-SV        2509
CrfoH1          LGGKLEMPWAFDRLFKLDITDTLHDMGIEPEDVFDTQAPFFLSYEVHAVNGTTLPLS-TI        2502
RaveH1          LGGALEMPWAFDRLYKYDITEDLHDLGIEPEDVFNVQAPFHLKYEIHAVNGSTLAPS-TI        2515
OcvuHA          LGGETEMPWHFDRTYKMDITMVMKEKGIAMESLFHHDSKVHLEMEIITIDGTVLDQN-SL        2482
TopaH1          LGGETEMPWHFDRNYKMDITDVLKKRNIPPEALFEHDSKIRLEVEIKSVDGAVLDPN-SL        2896
EuscH1          LGGETEMPWHFDRNYRLEITSVLEEMKISFDDLFQHESKIHLEVLIKNVDGSPLDAG-LL        2922
SeofH1          LGGETEMAWHFDRNYRFEITSVLEEMKIPFDKLFEHESKIHWEVEIIKVDGTPVDAG-VI        2921
SemaH           LGGETEMPWHFDRNYRFEITSVLEEMKIPFDKLFEHESKIHLEVEIVKVDGTPVDSG-VI        2922
LijaH2          LGSDLEMPWAFDRTFKYDITKALQKLGVSLS-----DDLYHLKVTIVAVNGTTLSND-VI        2432
NunuH1          LGGEFEMPWAFDRLYKYEITSAVKEAGLNPNDVFNAEAPFHLKLEITKVDGTQIPSS-EL        2490
MecrH1          LGGTKEMAWAYNRLFKYDITHALHDAHITPEDVFHPSEPFFIKVSVTAVNGTVLPAS-IL        2493
HacrH1          LGGSAEMHWAYNRLYKYDITEALHEFGINPEDVFHADEAFFLKVSVVAVNGTVLPPS-LL        2496
HarufH1         LGGSAEMHWAYNRLYKYDITEALHEFGINPEDVFHADEAFFLKVSVVAVNGTVLPPS-LL        2496
HadishaH1       LGGSAEMHWAYNRLYKYDITEALHEFGINPEDVFHADEAFFLKVSVVAVNGTVLPPS-LL        2496
HadivH1         LGGSAEMHWAYNRLYKYDITEALHEFGINPEDVFHADEAFFLKVSVVAVDGTVLPPS-LL        2411
HaasH1          LGGPAEMHWAYNRLYKYDITEALREFDINPEDVFHADEPFFLKLSVVAVNGSVIPPS-LL        2495
HarubH1         LGGSAEMHWAYNRLYKYDITEALHEFDINPEDVFHADEPFFLKLSVVAVNGTVIPSS-LL        2496
                **.  *  * ::* :  :**  :    :  .           .     ::.  :     .

CoasHaN         PQPTIIFDPPHEGAED---V-SSTSIAGIGVRKDVSTLSTSEIYNIRSALRQVKDDAGAN        2570
HepoHaN         PQPTIIFDPPHEGAED---V-SSTSIAGVGVRKDVSSLSTSEIDNIRSALQQVEDDTGPN        2569
HeluHaD         PKPSVTYKPALGVEKD---I-HTTAVAGVGVRKDVTRLTVSETENLREAPRRIKADNGSN        2566
ApcaH1          PEPTIIYKAAVGASEE---VSSSSSLAGVGVRKDVSTLSTSEIDNLREALRRVQADAGPN        2570
LystH1          PTPSIIYDPAKGVANV---D-ITSAVAGVGVRKDVSSLTASETESLRNALRKVQEDEGPN        2572
PhacH1          PTPSLIFDPAHGAAED---V-VSTSIAGVGVRKDVSTLSVSETENLRNALRKVQADNGPN        2574
```

```
PocaH1         PPPTIIYEPASGGFRG---M-PSYSLAGIGVRKDINTLSEAETENLREALSSVMDDTRII   2556
MetuMH         NAPTLIFVPATGASVD---A-KSYAVAGMSVRKDINTLSSAETQSLKNSLQKVMDGEGPL   2579
LisaH1         SPPTIIFEPAHGASQD---H-TSYSVAGVGVRKDINTLTAEETESLRDSLREMQEGGSGL   2565
CrfoH1         SPPTLVFQPAEGAAAE---H-SSYSIAGVGVRKDINTLTAAEMENLRDALGRVQAGTGRL   2558
RaveH1         SAPTLIFKPAEGASVD---Q-TSYSIAGVGVRKDINTLTAAEMKNLRDALRRVQAGTGRL   2571
OcvuHA         PKPSLVYSPAKGSVHQHHHH-HKEYAEGTIVRKNVNSLTPSEIDNLRHALSDVMADKSEN   2541
TopaH1         PKPSLIYAPAKGLIIQ---Q-VGEYDAGSMVRKNVNSLTPSEIENLRNALAAVQADKTDA   2952
EuscH1         PKPSLIYLPAKHHAVE---K-EQPEMAGTLIRKNVDSLTPYEVGNLRDALAAVQADITKT   2978
SeofH1         PKPSLIYLPGKPHLIE---K-EKPHSAGAAVRKNVNSLTPYETNNLRMALAAVQADITDT   2977
SemaH          PKPSLIYLPGKPHHIE---K-EKPHAAGAAVRKNVNSLTPYETDNLRDALAAVQADITDT   2978
LijaH2         PAPTLTFVPASRSGRQ---A-EADHAAAPGIRKNVKDLSSSEMENLRQVQQDPSSR   2488
NunuH1         HKPTIIYEPAHGHESS---V-GVASHAGRGVRKNVNTLTPSEIDNLKDAMRAVQADKGVN   2546
MecrH1         HAPTIIYEPGLDHHED---H-HSSSMAGHGVRKEINTLTTAEVDNLKDAMRAVMADHGPN   2549
HacrH1         HEPTILYEPGVGHHED---H-ESGSLAGSGVRKDVNTLTTAETDNLRRALRGVKEDHGHN   2552
HarufH1        HEPTILYEPGVGHHED---H-ESGNLAGSGVRKDVNTLTTAETDNLRKALRGVKEDHGHN   2552
HadishaH1      HEPTILYEPGVGHHED---H-ESGSLAGSGVRKDVNTLTTAETENLRKALRGVKEDHGHN   2552
HadivH1        HEPTILYEPGVGHHED---H-ESGSLAGSGVRKDVNTLTTAETENLRKALRGVKEDHGYN   2467
HaasH1         HQPTIIYEPGEDHHED---H-ESGSIAGSGVRKNVNTLTKAETDNLREALRGVMDDHGPN   2551
HarubH1        HQPTIIYEPGEGHHDD---H-ESGSIAGSGVRKDVNTLTKAETDNLREALKGVMDDHGPN   2552
                 *:: :                    .  :**::  *:  *  .:: :   :  .

CoasHaN        GFLNIASFHGKPARCEH-DHHPVACCVHGSPKFPHWHRLYVKQWEDALTAHGAKIGIPYW   2629
HepoHaN        GFQNIASFHGSPARCEH-DHHPVACCVHGSPNFPQWHRLYVKQWEDALTAHGAKIGIPYW   2628
HeluHaD        GFQSIASFHGSPPGCVH-ENHSVACRIHGMANFPQWHRLYVKQWEDALTAQGAKIGIPYW   2625
ApcaH1         GFASIAAFHGEPAGCEL-NGRRIACCQHGMTNFPQWHRLFVKQFEDALTAQGTMIGIPYW   2629
LystH1         GFQNIASFHGSLAKCEY-QGHPIACCLHGMANFPQWHRLYVKQWEDALTSQGSKVGIPYW   2631
PhacH1         GFQNIASFHGSPARCEH-NGHSIACCLHGMANFPQWHRLYVKQWEDALTAQGAMIGIPYW   2633
PocaH1         SYQRLAGWHGFPGLCSM-NGQQVACCQHGSASFPHWHRLYVRSLEIAMTLEGARIGIPYW   2615
MetuMH         SYQKIAEMHGWPGDCEQ-NGKKVACCHHGMASFPQWHRVYTRLLEMAMTWQGANVGIPYW   2638
LisaH1         PFQQFASWHGWPASCEH-DGHPVACCHHGMASFPQWHRLYVRILEMVMTFRGARVGIPYW   2624
CrfoH1         TYDNVVSAHGYPPQCTH-DGHKVACCQHGMASFPMWHRLFTRQMEVALSWEGAKVGIPYW   2617
RaveH1         TYDFIAGAHGYPAACKM-GEYDVACCQHGMASFPGWHRVFTRQMEVALSWEGAKVGIPYW   2630
OcvuHA         GYQKIAAYHGMPLSCKYPNGTAYACCQHGMVTFPHWHRLFVKQMEDALKLKGAKTGIPYW   2601
TopaH1         GYQKIASFHGMPLSCQYPDGTAFACCQHGMVTFPHWHRLYMKQMEDALKAKGAKIGIPYW   3012
EuscH1         GYQKIASYHGMPLSCKYDNGTALACCQHGMVTFPHWHRLYMKQMEDAMRARGARVGIPYW   3038
SeofH1         GFQKIASFHGMPLSCKYDNGSAYACCQHGMVTFPHWHRLYMKQMEDALRARGAKVAIPYW   3037
SemaH          GFQKIASFHGMPLSCKYDNGSAYACCQHGMVTFPHWHRLYMKQMEDALRARGAKVGIPYW   3038
LijaH2         GYQAIAAYHGLPAKCKNGNGQKMACCLHGMANFPHWHRLYTKQMEDALAEKGARLGMPYW   2548
NunuH1         GYQSIAAYHGLPAQCG-----DYACCLHGMPTFPHWHRLYVKQMEDALAAHGAEVGMPYW   2601
MecrH1         GYQAIAAFHGNPPMCPMPDGKNYSCCTHGMATFPHWHRLYTKQMEDALTAHGARVGLPYW   2609
HacrH1         GFQAIAAFHGKPAMCPMPDGHNLSCCTHGMATFPHWHRLYTKQMEDALKAHGSHVGLPYW   2612
HarufH1        GFQAIAAFHGKPAMCPMADGHNLSCCTHGMATFPHWHRLYTKQMEDALKAHGSHVGLPYW   2612
HadishaH1      GFQAIAAFHGKPAMCPMADGHNLSCCTHGMATFPHWHRLYTKQMEDALKAHGSHVGLPYW   2612
HadivH1        GFQAIAAFHGKPAMCPMADGHNLSCCTHGMATFPHWHRLYTKQMEGALKAHGSHVGLPYW   2527
HaasH1         GFQAIAAFHGKPAMCPMPDGHNYSCCTHGMATFPHWHRLYTKQMEDALRAHGSLVGLPYW   2611
HarubH1        GFQAIAAFHGKPALCPMPDGHNYSCCTHGMATFPHWHRLYTKQMEDALKSHGSLVGLPYW   2612
                :  ..  **    *        :*  **  .** ***:: :  * .:  .*:  .:***

CoasHaN        DWTYAFKKLPSLVT-AEVDNPFHHGVTHDG---HVTTRAPRSLLFNDPEFGDESFFYRQV   2685
HepoHaN        DWTYAFKKLPSLVT-AGDNNPFHHGVTHDG---HITTRAPRSLLFNDPEFGDESFFYRQV   2684
HeluHaD        DWTTAFTELPALVT-EEVDNPFHHGTIYNG---EITTRAPRDKLFNDPEFGKESFFYRQV   2681
ApcaH1         DWTTAFTALPSLVT-EQENNPFYNFKIFNG---EVTSRAPREQLFNDPEFGSESFFYRQA   2685
LystH1         DWTTAFTALPTLVT-EETNNPFHHGKIYNG---EITTRAPRDQLFNDPEFGSESFFYRQA   2687
PhacH1         DWTTAFTALPTLVT-EENNPFHHGKIYNG---EITTRAPREQLFNDPEFGSESFFYRQT   2689
PocaH1         DWTTTFTNLPSLLTADDSSNPFLKGHIKALN--QSTSRSPRPQLFNDPERGEESFFYRQI   2673
MetuMH         DWTEAFTALPSLVT-DDGDNPFNHGHIEALG--KVTSREPRPQLFKDPEHGEESFFYRQI   2695
LisaH1         DWTQAFTELPALVT-EEENNPFHHGVIEAL--NMTTSRAPRPQLFNDPEHGDESFFYRQV   2681
CrfoH1         DWTEAFTELPALVR-EEENNPFHHGRIPGTD--TVTTRAPRPQLFRDPEHGDESFFYRQV   2674
RaveH1         DWTEAFTELPTLVS-EDHDNPFHHGHVPGKAENITTTRAPRPQLFKDPEHGEESFFYRQA   2689
OcvuHA         DWTTPFTDLPTLIT-EETDNPFHHAHIDSVD--EITTRAPRHQLFEG-----RHFFYDQM   2653
TopaH1         DWTTAFHSLPILVT-EPKNNPFHHGYIDVAD--TKTTRDPRPQLFDDPEQGDQSFFYRQI   3069
EuscH1         DWTIAFSSLPDLVT-DEKNNPFHHAHIDVAN--TITTRNPRPQLFDDPEQGDESFFYRQI   3095
SeofH1         DWTTAFSSLPALVT-DEKNNPFHHSYIDVAG--TITTRSPRPQLFDDPEHGDESFFYRQI   3094
SemaH          DWTTAFSSLPALVT-DEKNNPFHHSYIDVAG--TITTRSPRPQLFDDPEHGDESFFYRQI   3095
LijaH2         DWTMPFTSLPHLVT-ETENNPFNKVKIVFKN--KYTTRDPVPNLFRDPEYGEKSFFYRQV   2605
NunuH1         DWTAAFTKLPALVT-DPNDNPWVHAHIEYLN--EVTTRAPRPQLFKDPEHNEGSFFYRQM   2658
MecrH1         DGTTAFTALPTFVT-DEEDNPFHHGHIDYLG--VDTTRSPRDKLFNDPERGSESFFYRQV   2666
HacrH1         DWTAAFSHLPTLVT-DSDNNPFHDGTIEYLN--VTTTRSPRDLLFNDPEHGSESFFYRQV   2669
HarufH1        DWTAAFSHLPTLVT-DNDNNPFHDGTIEYLN--VTTTRSPRDSLFNDPEHGSESFFYRQV   2669
HadishaH1      DWTAAFSHLPTLVT-DNDNNPFHDGTIEYLN--VTTTRSPRDLLFNDPEHGSESFFYRQV   2669
HadivH1        DWTAAFSHLPTLVT-DNDNNPFHDGTIEYLN--VTTTRSPRDLLFNDPEHGSESFFYRQV   2584
HaasH1         DWTTAFTHLPTLVT-DTENNPFNHGHIEYLD--VDTTRSPRDMLFNDPEHGSESFFYRQV   2668
HarubH1        DWTTAFTHLPTLVI-DTKNNPFNHGHIEYLD--VDTTRSPRDMLFNDPEHGSESFFYRQV   2669
               * *  *  ** ::     .**: .           *:* *   ** .       *** *

CoasHaN        LLAFEQTDYCDFEVQFEITHNAIHSWVGGQSPYGLSTLEYTAYDPLFLLHHSNVDRQFAI   2745
HepoHaN        LLAFEQTDYCDFEVQFEITHNAIHSWVGGQSPYGLSTLEYTAYDPLFLLHHSNVDRQFSI   2744
```

```
HeluHaD       LLAFEQTDYCDFEVQYEISHNAIHSWTGGQSPYGMSTLEYTAYDPLFLLHHSNVDRQFAI      2741
ApcaH1        LLAFEQTDYCDFEVQYEITHNAIHSWTGGRSPYGMSTLEFTAYDPLFLLHHSNADRQFAI      2745
LystH1        LFTFEQTDYCNFEVQFEITHNAIHSWTGGSSQYGMSSLEYTAYDPLFLLHHSNVDRQFAI      2747
PhacH1        LLAFEQTDYCDFEVQYEINHNAIHSWTGGVSPYGMSTLEYTAYDPLFLLHHSNVDRQFAI      2749
PocaH1        LLAFEQRDYCDFEVQFEVTHNAIHSWIGGTSPYGMSTLEYSAYDPIFFIHHSNVDRQFAI      2733
MetuMH        LLAFEQRDYCDFEVQFEVTHNAPHSWIGGTSPYGMSTLEFSSYDPVFFLHHSNVDRQFAI      2755
LisaH1        LLAFEQRDYCDFEVQFEVAHNAIHSWIGGTSPYGMSTLEYSAYDPIFFIHHSNVDRQFAI      2741
CrfoH1        QLALEQRDYCDFEVQFEVIHNAIHSWIGGTSPYGMSTLEYSAYDPVFFIHHSNVDRQFAI      2734
RaveH1        LLAFEQRDFCDFEVQFEVLHNAIHSWIGGTSPYGMSTLEYAAYDPLFFIHHSNVDRQFAI      2749
OcvuHA        MFALEQTNFCDFEIQFEISHNAIHSWVGGSSPYSMSTLHYTSYDPLFYLHHSNVDRIWAI      2713
TopaH1        AFALEQRDFCDFEIQFEMGHNAIHSWVGGPSPYGMSTLHYTSYDPLFYVHHSNTDRIWAI      3129
EuscH1        AFALEQRDFCDFEIQFEMGHNAIHSWVGGSSPYGMSTLHYTSYDPLFYLHHSNTDRIWAI      3155
SeofH1        ALALEQRDFCDFEIQFEMGHNAIHSWVGGPSPYGMSTLHYTSYDPLFYLHHSNTDRIWAI      3154
SemaH         ALALEQRDFCDFEIQFEMGHNAIHSWVGGPSPYGMSTLHYTSYDPLFYLHHSNTDRIWAI      3155
LijaH2        LFALEQTDYCDFEIQFEVSHNAIHSWVGGSSPYSMSTLHYTSYDPLFFLHHSNTDRIWAI      2665
NunuH1        LLAFEQTDYCDFEVQFEMTHNAIHSWTGSASPYGLSSLDFTSYDPLFYVHHSNTDRLWAM      2718
MecrH1        LLALEQTDFCQFEVQFEITHNAIHSWTGGLTPYGMSTLEYTTYDPLFWLHHANTDRIWAI      2726
HacrH1        LFALEQTDFCKFEVQFEITHNAIHSWTGGHSPYGMSTLDFTAYDPLFWLHHSNTDRIWAI      2729
HarufH1       LFALEQTDFCKFEVQFEITHNAIHSWTGGHSPYGMSTLDFTAYDPLFWLHHSNTDRIWAI      2729
HadishaH1     LFALEQTDFCKFEVQFEITHNAIHSWTGGHSPYGMSTLDFTAYDPLFWLHHSNTDRIWAI      2729
HadivH1       LFALEQTDFCKLEVQFEITHNAIHSWTGGHSPYGMSTLDFTAYDPLFWLHHSNTDRIWAI      2644
HaasH1        LLALEQTDFCQFEVQFEITHNAIHSWTGGHSPYGMSTLDFTSYDPLFWLHHSNTDRIWAI      2728
HarubH1       LLALEQTDFCQFEVQYEITHNAIHSWTGGHSPYGMSTLDFTAYDPLFWLHHSNTDRIWAI      2729
               :::** ::*.:*:*:*: *** *** *. : *.:*:*.:::***:* :**:*.** :::

CoasHaN       WQALQKHRGLPYNTANCAIQELRRPLRPFSDKENVNPTTRANSRAIDAFSSDRLHYQYDN      2805
HepoHaN       WQALQKYRGLPYNTANCAIQDLRRPLRPFSDPANVNPTTRANSRAIDAFNADSLHYQYDN      2804
HeluHaD       WQALQKFRGLPYNSANCAIQLLHQPMRPFSDADNVNPVTRTNSRARDVFNYDRLNYQYDD      2801
ApcaH1        WQALQKFRGLPYNNANCAIQKLREPMKPFSNLDNINPTTRANARAIDAFDYDRFNYQYDN      2805
LystH1        WQALQKFRGLPYNSANCAIQHLRQPMRPFSDENNLNAITRANSRAIDAFDYDRLNYQYDN      2807
PhacH1        WQALQKFRGLPYNSANCAIQHLRQPMKPFSEESNINPTTRANSRGIDAFDYDRLNYQYDN      2809
PocaH1        WQALQRYRGLEHNSANCNIQELKMPLEPFNRKENLITIIRENSRAIDAFNYEQFGYQYDN      2793
MetuMH        WQALQKYRGLDYNSANCDIQELRMDLEPFNRDYIPIVIIRNNARAIDAFNYDQYSYQYDN      2815
LisaH1        WQALQKYRGLDYNTANCNIQELRQTLEPFDSPHNPVSIIREHATAIDAFNYDQFSYQYDN      2801
CrfoH1        WQALQKYRGLDYNTANCNIQELRQVQEPFDRDDNPVVTTRHYSKAIDAFNYDQYGYQYDN      2794
RaveH1        WQELQKHRGLDYNTANCHIQDLRKPLEPFNRAGNPVLVTRAHSRAIDAFNYDQYGYQYDH      2809
OcvuHA        WQALQKYRGLPYDSANCEIHRLKKPLNPFSSDANHNEQTRTHSTGESSFDYHKLHYDYDN      2773
TopaH1        WQALQKYRGLPYNSANCEINKLKKPMMPFSSEDNPNEVTKAHSTGYKSFDYQQLNYEYDN      3189
EuscH1        WQALQKYRGLPYNSANCEINKLKKPMEPFSYEENPNPVTKAHSTGMKVFDYHELNYEYDN      3215
SeofH1        WQALQKYRGLPYNSANCEINKLKKPMMPFSSDDNHNEVTKAHSTGIKSFDYHELNYEYDN      3214
SemaH         WQALQKYRGLPYNSANCEINKLKKPMMPFSSDDNHNEVTKAHSTGIKSFDYHELNYEYDN      3215
LijaH2        WQALQKYRGLPYKYANCAIPLLRKPMRPFSDDDNWNAITRTNSRPIDVFDYQRLGYQYDH      2725
NunuH1        WQALQRFRGKPYNTAYCALEQLKKPIRPFSDASNPNPVTRAHARALRSFNYEALNYQYDT      2778
MecrH1        WQALQEYRGLPYDHANCEIQAMKRPLRPFSDPINHNAFTHSNAKPTDVFEYSRFNFQYDN      2786
HacrH1        WQALQKYRGLPYNHANCEIQAMKTPLRPFSDNINHNPVTKANSRPVDVFEYNRLRYQYDN      2789
HarufH1       WQALQEYRGLPYNHANCEIQAMKTPLRPFSDNINHNPVTKANSRPVDVFEYNRLRYQYDN      2789
HadishaH1     WQALQKYRGLPYNHANCEIQAMKTPLRPFSDNINHNPVTKANSRPVDVFEYNRLRYQYDN      2789
HadivH1       WQALQKYRGLPYNHANCEIQAMKTPLRPFSDNINHNPVTKANSRPVDVFEYNRLRYQYDN      2704
HaasH1        WQALQEHRGLPYNHANCEIQAMKTPLRPFTDDINKNPVTKANSKPVDVFEYNRLSFQYDN      2788
HarubH1       WQALQEYRGLPYNHANCEIQAMKTPLRPFSDEINHNPVTKANSKPVDVFEYNRLSFQYDN      2789
              ** **..**  :. * * :  ::    **          :  :     *.     ::**

CoasHaN       LDFHGHSIVELEEILQHRQEEDRVFAEFLLHGLKTSADVTFDLCDERNHCEFAGTFAILG      2865
HepoHaN       LNFHGHSIAELEEILQHRQEEDRVFAEFLLHGLKTSADVSFDLCDERGHCEFAGTFAILG      2864
HeluHaD       LNFHGLSISELNDVLERRKEKARIFAEFLLHGIGASADVTFDLCDSHDHCEFAGTFAILG      2861
ApcaH1        LNFHGLTISELNDLLEKRKEEDRVFAEFLLAGFGGSGDVVFNLCSEE-ECAFAGTFAVLG      2864
LystH1        LNFHGLTISQLNDLLEKRKEEDRIFAEFLLRGFSASADVIFKLCDEKGHCEFAGTFAVLG      2867
PhacH1        LNFHGLTISELNDVLERRKEEDRIFAEFLLRGFALSADVTFRLVDDKGHSEFAGTFAVLG      2869
PocaH1        LNFHGLTIPELEAVLEARRQEDRVFANFMLHGIRSSADVSFDICDAQNHCIFAGTFAILG      2853
MetuMH        LNFHGMSIPELEALLEKRRSEDHVFLNFMLLGIGASADVTFDICDSEGHCNFAGTFAVLG      2875
LisaH1        LNFHGLTISQLDDLLEERKDEDRVFANFLLHNIGTSADVEFDLCDAEEHCQFAGTFAILG      2861
CrfoH1        LNFHGMTISQLDEMLEKKKQEDHVFANFMLHGIQTSADVVFDLCDAKGKCNFAGTFAILG      2854
RaveH1        LRFHGLSVQELEEKLHERAEQDRVFLNFMLRGIKMSADVEFDLCNAQGTCNFAGTFAILG      2869
OcvuHA        LIFHGMTIPQLETELMKSQKKDRVYAGFLLRTIGQSADVEFEVCRKDGECKFAGTFCILG      2833
TopaH1        LNFHGMTIPQLEVHLKKIQEKDRVFAGFLLRAIGQSADVNFDVCRKDGECTFGGTFCVLG      3249
EuscH1        LNFHGMTIPQLEIHLEEIKQEDRVFAGFLLRAIGQSADVTFDICH-NGKCEFAGTFCVLG      3274
SeofH1        LNFHGMTIPQLEVHLNKIQEKDRVFAGFLLRAIGQSADVNFDICRKDGECHFGGTFCVLG      3274
SemaH         LNFHGMTIPQLEVHLNKIQEEDRVFAGFLLRAIGQSADVNFDICSKDGECHFGGTFCVLG      3275
LijaH2        LRFDGLDIPQLEVLLNENRQKDRVFAGFLLSGFQASADVRFDVCLASGECKFAGTFAVLG      2785
NunuH1        LSFNGMSIPELDDLVHERQEKDRIFAAFSLEGIKKTCDVKFDVCKSDGECKFAGTFAILG      2838
MecrH1        LRFHGMTIKKLEHELEKQKEEDRTFAAFLLHGIKKSADVSFDVCNHDGECHFAGTFAILG      2846
HacrH1        LVFHGHTIPELDHMLEERKKHDRIFAAFLLRGIKKSADVVFEICEPDHECVFAGTFAILG      2849
HarufH1       LVFHGHTIPELNHMLEERKKHDRIFAAFLLRGIKKSADVVFEICEPNHECVFAGTFAILG      2849
HadishaH1     LVFHGHTIPELDHMLEERKKHDRIFAAFLLSGIKKSADVVFEICEPNHECVFAGTFAILG      2849
HadivH1       LVFHGHTIPELDHMLEERKKHDRIFAAFLLSGIKKSADVVFEICEPNHECVFAGTFAILG      2764
HaasH1        LIFHEYNIPDLDRMLEERKKEDRIFAAFLLSGIKHSADVVFDICQPGHECVFAGTFAILG      2848
HarubH1       LIFHGYNIPDLDHMLEERKQEDRIFAAFLLSGIKHSADVVFDICQPGHECVFAGTFAILG      2849
```

```
                * *.   : .*:  :    .. : :  * *  :  : ** * :      . *.***.:**

CoasHaN         GDLEMDWEFDRLFKYDVTNVFNKLHLQPDSAYHFVDHITAVNGTELDSNLIRPPSVHFVP          2925
HepoHaN         GYLEMDWEFDRLFRYDVTNVFNKLHLQPDSDYHFVDHITAVNGTELDSQLIRPPSVHFVP          2924
HeluHaD         GPLEHPWAFDRLFKYDVTDVFSKLHLRPDSEYHFNIHIVAVNGTELDSHLIRSPTVQFVP          2921
ApcaH1          GAIEMPWAFDRLFKYDVTNVFRKLNLHPDDVYHFEVKIMAVNGTELYPGLIRPPSVQFVP          2924
LystH1          GLLEMPWAFDRLFKYDVTNVFNKLNLRTDSDYHFDVEIKAINGTVLDSNLIRSPSVQFVP          2927
PhacH1          GPLEMPWAFDRLFKYDVTNVFNKLNLRPDSNYHFEVEIKAINGTILDSNLIRSPSVQFVP          2929
PocaH1          GPLEMPWVFDRLFKYDVTSVFKQLHLRPDSEYRFKMRLTAVNGTELDPHLLHAPSVSFLP          2913
MetuMH          GPLEMPWSFDRLFKYDVTSVFRQMHLRPDSDYSVKVHIVSVNGTELDSDLLEAPSVSFVP          2935
LisaH1          GPLEMAWRFDRLFKYDVTSVFRQMRLRPDSVYHFRVHLTAVNGTELDAHLIEDPSVSFLP          2921
CrfoH1          GPLEMPWSFDRLFKYDVTSVFKQMRLRPDSEYSFRVSLTAVNGTQLDSRLIEAPSVSFVP          2914
RaveH1          GPLEMPWNFDRVFKYDVTKIFRQMRLRPDSNYTIPIRIRAVNGMQLDPDLLEPPSVTFAP          2929
OcvuHA          GKHEMFWAFDRLYKYDITSTLEQLRLHAHDQFSITVSIKATDGSTLAPSLIPPPSIMYHP          2893
TopaH1          GDYEMPWAFDRLFLYDISKSLVHLRLDAHDDFDIKVTIMGIDGKSLPPNLLPSPTILFKP          3309
EuscH1          GEHEMAWAFDRLFLYDITKALTKLSIDAYDDFKVQVGITSITGEKLPQSLLPPPTILFKP          3334
SeofH1          GQHEMAWAFDRLFLYDITKALNKLHLDAYDDFLINVSIVNIEGVKLPSSLLPRPTIMFKP          3334
SemaH           GQHEMAWAFDRLFLYDITKALNKLHLDAYDDFVINVSIVNIEGVKLPSRLLPKPTIMFKP          3335
LijaH2          GTKEMPWAFDRLFKYEITEALSALRADHHSVFTVETTIIATNKTKLASNLIPRPTVIYEP          2845
NunuH1          SEYEMHWRYDRAFRYDITDTMEELHLKPESEFTIKLHLVQADGTELSSDLVDSPEVIFVP          2898
MecrH1          GEHEMPWSFDRLFRYDITQVLKQMHLEYDSDFTFHMRIIDTSGKQLPSDLIKMPTVEHSP          2906
HacrH1          GELEMPWSFDRLFRYDITKVMQQQHLRHDSDFSFKVKVVGTDDKELPPGILKEPTIEYEP          2909
HarufH1         GELEMPWSFDRLFRYDITKVMKQQHLRHDSDFSFKVKVVGTDDKELPHGILKEPTIEYEP          2909
HadishaH1       GELEMPWSFDRLFRYDITKVMQQQHLRHDSDFSFKVKVVGTDDIELPPGILKEPTIEFEP          2909
HadivH1         GELEMPWSFDRLFRYDITKVMQQQHLRHDSDFSFKVKVVGTDDIELPPGILKEPTIEFEP          2824
HaasH1          GDQEMPWAFDRLFRYDITKVMKQLHLRHDADFTFSVKIVSTDGQELPSDSVKTPTIEFEP          2908
HarubH1         GELEMPWSFDRLFRYDITKVMKQLHLRHDSDFTFRVKIVGIDDHELPSDSFKAPTIEFEP          2909
                .  *  * :** : *:::. :          : .   :       *    .  * : . *

CoasHaN         GVKKPHG-VAAAS---GPASGVLVRKNINQLSQDEAVSLRDALYQLQQDQGLGGFEAIAG          2981
HepoHaN         GVKKPLG-VAAAS---GPGSGVLLRKNVNQLSQDEAVSLRDALYQLQQDQGLGGFEAIAG          2980
HeluHaD         GVKDYYEKIAQKT---EAHEDVLLRKNINELSLEESANLRSALNKLQQDQGPNGFEAIAG          2978
ApcaH1          GVKGYYERVAAKT---AKSSASVLRKDVNDLTLAEASNLRDALYKLQQDQGPNGFEAIAG          2981
LystH1          GVKRFYEKVAEKT---VVRDDKLVRKNVNQLTLDEAANLRNALNQLQNDQGPNGFEAIAG          2984
PhacH1          GTKKFYEKIAEKT---AKRDDKLVRKNINQLTLDEAANLRNALNKFQQDQSDEGYEATAG          2986
PocaH1          GRGEQRA-RAAREDPVTTVSSVVTRYDVDTLTLEQASSLRNALHKLQNDHGLTSYEAIAS          2972
MetuMH          GKRGR----KAREDYRPTVSEDLIRKEVSSLSLEEVSNLKNALYKLQNDHGPNGFEAIAS          2991
LisaH1          GRDHGVSHAQAHEDPIPLASEDVTRYEVSSLSLEQSSNLRNALYKLQNDDGPNGFEAIAS          2981
CrfoH1          GNKPGSKTSAAHEDPVPLDTGDVTRYEVSALSLAQVTNLRDALYKLQNDHGPNGFEAIAS          2974
RaveH1          GKKSGSGRAVVYEEPTPSETQDLTRHDLSSLSMAQISNLEDALYKLQNDHGPNGFEAIAS          2989
OcvuHA          AAA---------------------------------------------------------          2896
TopaH1          GTGKI-------------------------------------------------------          3314
EuscH1          GKGSQQHH----------------------------------------------------          3342
SeofH1          GKGTQHHH----------------------------------------------------          3342
SemaH           GKGTQHHH----------------------------------------------------          3343
LijaH2          GKAST---STKRV--TQPKRTALLRKGLNTLTLDEAKNLREAMFKMMNDEGPNGFEAIAT          2900
NunuH1          GSAT---HEHHTV--HEDHRDVLLRKNINSLSLEEARSLRDALYKLQNDQTENGFEHIAG          2953
MecrH1          ----GGKHHEKHH--EDHHEDILVRKNIHSLSHHEAEELRDALYKLQNDESHGGYEHIAG          2960
HacrH1          ALGDGLKHEDEGH--DDRLSHVLIRKEVDFLSLKEANAIKDALYKLQNDHSKGGFEEIAG          2967
HarufH1         ALGDGLKHEDEGH--DDRLSHVLIRKEVDFLSLKEANAIKDALYKLQNDHSKGGFEEIAG          2967
HadishaH1       ALGDGLKHEDEGH--DDRLSHVLIRKEVDLLSLKEANAIKDALYKLQNDHSKGGFEEIAG          2967
HadivH1         ALGDGLKHEDEGH--DDRLSHVLIRKEVDLLSLKEANAIKDALYKLQNDHSKGGFEEIAG          2882
HaasH1          GVQDGRKLDGEHH--DDRHSDVLIRKEVDFLSLQEANAIKDALYKLQNDDSKGGFEAIAG          2966
HarubH1         GVHDGGKHDDGHH--DDRHSDVLIRKEVDFLSLQEANAIKDALYKLQNDNGKGGFEAIAG          2967


CoasHaN         FHGAPFLCPEHGAE-KYACCVHGMPSFPHFHRLFTVQFEQSLKQHGSTTGIPYWDWTSPG          3040
HepoHaN         FHGAPFLCPENGDK-KYACCVHGMPTFPHWHRLFTVQFEQALKLHGSTTGIPYWDWTSPG          3039
HeluHaD         FHGAPFKCPENGTD-KYACCVHGMSVFPHWHRLLTVQFEQALKAHGAKEGVPYWDWTAPI          3037
ApcaH1          YHGAPFKCPANGED-KYACCAHGMPVFPHWHRLHTVQFEQALKEHGALVGVPYWDWTAPI          3040
LystH1          FHGAPFRCPATGDD-KYACCVHGMPVFPHWHRLFTVQIEQALKSNGALIGVPYWDWTTPV          3043
PhacH1          FHGAPFKCPADGDD-KFACCVHGMPVFPHWHRLFTVQVEQSLKKNGALVGVPYWDWTAPV          3045
PocaH1          FHAGSGLCPENASV-TFACVPHGFANLPHFNRLLLVQMEMALLEKGATTGIPYWDWTRTI          3031
MetuMH          FHGDPGLCPEGSGS-KYACCQHGMPVFPHWHRLLTVQFERALKAKGSVVGVPYWDWTRPS          3050
LisaH1          FHGAPGLCPENGGKLRIACCRHGMPAFPHWHRLLTVQFERALRDKGAVVGVPYWDWTRPV          3041
CrfoH1          FHGAPGLCPENATD-HYACCRHGMPAFPHWHRLLTVQFERALKDKGAVVGVPYWDWTRPA          3033
RaveH1          YHGAPGLCPENATE-HYSCCQHGMPAFPHWHRLLTVQFEHALKEKGAMVGVPYWDWTRPA          3048
OcvuHA          ------------------------------------------------------------          2896
TopaH1          ------------------------------------------------------------          3314
EuscH1          ------------------------------------------------------------          3342
SeofH1          ------------------------------------------------------------          3342
SemaH           ------------------------------------------------------------          3343
LijaH2          FHGAPGKCPEGATENTFSCCIHGMPMFPHWHRLFVLQAEKSLQDHGATVGIPYWDWTRSM          2960
NunuH1          FHGEPNVCPEDSE-SKYACCAHGMPIFPHWHRLLTVQFERGLVDNGALIGLPYWDWTVPS          3012
MecrH1          FHGYPNLCPEKGD-EKYPCCVHGMSIFPHWHRLHTIQFERALKKHGSHLGIPYWDWTQTI          3019
HacrH1          YHGYPNKCPEKGD-DKYPCCVHGMPIFPHWHRLHTIQMERALKNHGSQIGIPYWDWTKRM          3026
HarufH1         YHGYPNKCPEKGD-DKYPCCVHGMPIFPHWHRLHTIQMERALKNHGSQIGIPYWDWTKRM          3026
```

HadishaH1	YHGYPNKCPEKGD-DKYPCCVHGMPIFPHWHRLHTIQMERALKNHGSQIGIPYWDWTKRM	3026
HadivH1	YHGYPNKCPEKGD-DKYPCCVHGMPIFPHWRRLHTIQMERALKNHGSQIGIPYWNWTKRM	2941
HaasH1	YHGYPNMCPERGS-EKYPCCVHGMPVFPHWHRLHTIQMERALKNHGSPMGIPYWDWTKKM	3025
HarubH1	YHGYPNMCPEHGT-DKYPCCVHGMPVFPHWHRLHTIQMERALKNHGSPMGIPYWDWTKKM	3026

CoasHaN	NELPLFLADTDSDNPFSGYTISFAGQRTSRNPLEALFSTN--TSAGTSLLYQLTLDALEE	3098
HepoHaN	NELPLFLADTDNDNPFSSYTISFVGQRTSRNPLGALFSTN--TSAGTSLLYQLTLDALEE	3097
HeluHaD	GKIPSLFGDSADYNPFYSYTISFNNQRTTRDIQSELYNPH--QINGYNYLYYLALSTLEE	3095
ApcaH1	NALPSLIGDSSNHNPFYKYHISFVNQDTTRDIQDSLFNPR--TINGYNYLYYLALSTLEE	3098
LystH1	KSLPSFFGDDADHNPFASFRIAAANEKTTRQVQSELYSER--KVHGFPYLFYLALTTLEE	3101
PhacH1	QSLPSLFGDSSDFNPFYSFQIASANHKTTRDVQQDLYSNR--KVNGYHYLFYLALNTLEE	3103
PocaH1	RALPSLVAES-GDNSFFSYHIRQANKDTVRDPQEDLYVAS---RGTRNILFEQTLLALEE	3087
MetuMH	RGIPQLFSNTYGNNPFLTYEIQAASAFVERDVQVDALEHTTGHEGFEDSLFHQALETLEE	3110
LisaH1	KALPSLLTDPYDNNPFRSYRISFVEQYTSRDVQAELFKHP--SEGDLESLFHQALETLEE	3099
**CrfoH1**	**KAMPSLFTDSYDNNPFQTYRMTFNDQYIQRDVSEELFNHP--SEGDVESLFHQALETLEE**	**3091**
RaveH1	KGMPTLFTDNSAINPFYTYLMSFNNHHIERKVHEDLFSHS--AEGDPESLFHQALELLEE	3106
OcvuHA	--	2896
TopaH1	--	3314
EuscH1	--	3342
SeofH1	--	3342
SemaH	--	3343
LijaH2	KALPNFVSQAE-NNPWHRGYIRFEGKYTTRTVDPRLFDPP-QVS-GRNFLHHNVLLSLEE	3017
NunuH1	KALPSLFAEES-NNPFYKYHIGFANTDTEGDVQDFLFNQP-SFYGKYNYLYYLALTTLEE	3070
MecrH1	SSLPTFFADSGNNNPFFKYHIRSINQDTVRDVNEAIFQQT-K-FGEFSSIFYLALQALEE	3077
HacrH1	SSIPAFFGDDSNNNPFYKYHIRAVNQYTTRDVDVDLFNQT-K-FGEYDYLYYLTLQVLEE	3084
HarufH1	SSLPAFFGDDSNNNPFYKYHIRAVNQYTTRDVDVELFNQT-K-FGEYDYLYYLTLQVLEE	3084
HadishaH1	SSIPAFFGDDSNNNPFYKYHIRAVNQYTTRDVDVELFNQT-K-FGEYDYLYYLTLQVLEE	3084
HadivH1	SSIPAFFGDDSNNNPFYKYHIRAVNQYTTRDVDVELFNQT-K-FGEYDYLYYSTLQVLEE	2999
HaasH1	SSLPAFFGDSGNNNPFYKYYIRGVHHETTREINQQMFNQT-K-FGEYDYLYYLTLQVLEE	3083
HarubH1	SSLPAFFTDAGNGNPFYKYYIRGVQHETTREINPIIFNQT-K-FGEYDYLYYLSLQVLEE	3084

CoasHaN	DDYCQFEIMLEFFHNRIHFLIGGSETYSMSTLDYSAFDPIFMIVHSGMDRLWVLWQELQK	3158
HepoHaN	DDYCHFEIMLEFLHNRIHFLIGGTETYSMSTLDYSAFDPIFMIVHSGMDRLWVLWQELQK	3157
HeluHaD	DNFCDFEVQYEVLHNEIHALIGGNGTYSMATLDFSAFEPFFMIHHSSLDRIWIIWQELQK	3155
ApcaH1	DSFCDFEIQYEILHNEIHGLIGGHGTYSMSTLDYSAFDPLFMIHHSSIDRIWAIWQQLQK	3158
LystH1	DNYCDFEVQFEVLHNEIHADIGGSGTYSMATLDYSAFDPFFMIHHSSIDRIWVIWQELQK	3161
PhacH1	DNYCDFEVQFEVLHNEIHATIGGSGQYSMATLDYSAFDPFFMIHHSSIDRIWVIWQELQK	3163
PocaH1	VNFCDFLVQMDLLHVRVHALVGGKEAFSMATMEHAAFDPLFWLHAANVDRLWQAWQELQK	3147
MetuMH	TSYCEFEVQFEMLHNAVHALVGGTKTHSMATLEWSAFDPFFMVHHSSIDRIWRIWQELQR	3170
LisaH1	NNYCDFEVQYEMLHNAVHELVGGSRKYGMSTLEYSAFDPFFMIHHSSIDRIWKIWQTLQK	3159
**CrfoH1**	**TNYCDFEVQYEMLHNAVHELIGGGNTYSMSTLEYSAFDPFFMVHHASIDRIWQIWQSLQK**	**3151**
RaveH1	TNYCDFEVQYEMLHNAVHELVGGPHKYSMSTLEYSAFDPFFMVHHSSIDRIWQIWQTLQK	3166
OcvuHA	--	2896
TopaH1	--	3314
EuscH1	--	3342
SeofH1	--	3342
SemaH	--	3343
LijaH2	DDYCDFEVQFEIFHNTIHAWVGGADPRSLSTLEYSAFDPLFLIHHSNMDRLWIIWQELQK	3077
NunuH1	DNYCDFEVQFEVLHNAMHCFIGGTGAHSMNTLDYSAFDPFFIIHHSSIDRIWIIWNELQK	3130
MecrH1	DNYCDFEVQYEILHNEVHALIGGAEKYSMSTLEYSAFDPYFMIHHASLDKIWIIWQELQK	3137
HacrH1	NSYCDFEVQYEILHNAVHAYLGGAGKYSMSTLEYSAYDPVFMIHHSSLDRIWILWQQLQK	3144
HarufH1	NSYCDFEVQYEILHNAVHAWLGGAGKYSMSTLEYSAYDPVFMIHHSSLDRIWILWQQLQK	3144
HadishaH1	NSFCDFEVQYEILHNAVHAWLGGAGKYSMSTLEYSAYDPVFMIHHSSLDRIWILWQQLQK	3144
HadivH1	NSFCGFEVQYEILHSAVHAWLGGAGKYSMSTLEYSAYDPVFMIHHSSLDRIWILWQQLQK	3059
HaasH1	NSYCDFEVQYEILHNAVHAWLGGSGKYSMSTLEYSAFDPVFMIHHSSLDRIWILWQKLQK	3143
HarubH1	NSYCDFEVQYEILHNAIHAWLGGPGMYSMSTLEYSAFDPVFMIHHSSLDRIWILWQKLQK	3144

CoasHaN	LRRKPFVAIECGEK-SLHEPLHPFDYD-INTISLTRENAVPDSIFDHHHLQYDYDTTEIS	3216
HepoHaN	LRRKPFVAIECGEK-SLHEPLHPFDYD-INTIALTREHAVPDTLFDHHLLGYEYDTTEIS	3215
HeluHaD	LRHKPFNYAHCGGH-VLDDPLHPFSFGEINKNDLTRLNSQPSSVFDYTHFGYEFDKLELN	3214
ApcaH1	LRRKPFNSARCGGA-IMEEPLQPFSYSQINTNDFTRMNSQPSKVFDYAHLGYEFDNLELN	3217
LystH1	LRHKPFNGASCAGH-IMERPLQPFSYPEVNKNEFTRLNSVPNVVFDSERLGYKYDKLELN	3220
PhacH1	LRHKPFNSARCAGH-IMERPLQPFSYPEVNHNEFTRLNSVPNLVFDSERFGYQYDNLELN	3222
PocaH1	LRRRSYHSGSCARV-TEDTPMLPFSSETLNPNPVTHANARPVQSVEVDKFRYSYDHLDFN	3206
MetuMH	LRKKPFNVARCAMR-YLRKPLEPFSYASVNTDEVARTNSRPIDIFDTAKFHYDFDNLDLG	3229
LisaH1	LRHKPFNFARCAGR-SLFKPMEPFAYESVNTDPITRANAEPVHVFDTEKFHYHYDSLELN	3218
**CrfoH1**	**LRHKPFNYASCASR-SLYKPLEPFSYTSLNADPLTLNNAQPVHIFDTAKFHYHYDSLDLN**	**3210**
RaveH1	LRHKPFNYAVCAAR-SMYKPLEPFSYESINPDPLTRENSKPAQIFDTHKFHYHYDNLNLN	3225
OcvuHA	--	2896
TopaH1	--	3314
EuscH1	--	3342
SeofH1	--	3342
SemaH	--	3343
LijaH2	LRGKPYNSAPCAGR-KMSLPMQPFHFDSLNHNTLTKENSRAINAFDHHAFGYEYDSLDLN	3136

NunuH1 LRHKSFSYAECTKH-HLDRPLHPFNYASVNHNELTRTHSMPNQAADQSAFGYWYDNLDMN 3189
MecrH1 RRVKPAHAGSCAGD-IMHVPLHPFNYESVNNDDFTRENSLPNAVVDSHRFNYKYDNLNLH 3196
HacrH1 RRMKPYYAADCAGD-LMKVPMHPFSYKSENEDEFTRVNSVPNIVFDHYKFNYDYDNMRIR 3203
HarufH1 RRMKPYYAADCAGD-LMKFPMHPFSYKSENEDEFTRVNSVPNVVFDHYKFNYDYDNMRIR 3203
HadishaH1 RRMKPYYAADCAGD-LMKFPMHPFSYKSENEDEFTRVNSVPNIVFDHYKFNYDYDNMRIR 3203
HadivH1 RRMKLYYAADCAGD-LMKFPMHPFSYKSENEDEFTRVNSVPNIVFDHYKFNYDYDNMRIR 3118
HaasH1 RRMKPYYALDCAGDRLMKAPLHPFNYESVNEDEFTRANSYPNSVFDHYRFNYEYDNLRIR 3203
HarubH1 RRMKPYYAIDCAGDRLMKAPLHPFNYETVNEDEFTRTNSYPNIVFDHYRFNYEYDNLRVR 3204

CoasHaN GHDAAEVLEIIRRRHSETRVYLGGAAQGYGESYRTETWVLNDA--GEKFSVGTNFLLGSA 3274
HepoHaN EHDAAEVLEIIRRRHSETRVYLGGAAYGHGQSFRTAAWVLNDA--GEEFDAGTNFLLGSS 3273
HeluHaD GHDVQGIDDIIHNLRHENRVYLGFVLFGQQSSLEYKIDLIDDA--GQAHTAGSFHLLGGE 3272
ApcaH1 GHDVNDLNNIINRLRDQDRVYLAFNNCGKQGSFAFDVHFETAE--NNVVPLGRFYVLGGE 3275
LystH1 GNSVEEINNIIKNLHHQERIFVGILAFGHQKSLTIHISLINDN--DEAFDGGNIHILGGE 3278
PhacH1 GKNVQEIHEIINKLNQQDRVFIGFVIGGLQQSLTLDISLLLND--NGVAAAGAVHVLGGE 3280
PocaH1 RRSVSELLEATQSLRVKDRLFAAFLLSGVHTSARLHVTLQTGS-DEGAVEVGSIYLLGGL 3265
MetuMH GHSIGEVNKMINDMRSKTRLFVGFVLSGIETSATVKIEMDDSQ--GNSHEVGTFYVLGGH 3287
LisaH1 GHSVKQLQEMVDSMRNSPRVFAGFLLHGVGTSASVHVDVVAAD--GSKVNAGNFYVLGGE 3276
CrfoH1 GHGVHELHDMIEDMQATSRIFAGFVLSGISTSARVHVDVTRGE---DTVSVGNFYVLGGS 3267
RaveH1 GHSVAELNTMIHAMQAQSRTFAGFVLSGISTSARVHVDIYKGD---EVASVGNFFVLGGP 3282
OcvuHA -- 2896
TopaH1 -- 3314
EuscH1 -- 3342
SeofH1 -- 3342
SemaH -- 3343
LijaH2 DLGVNQLLAYIKEIKQHDRFFVGFNTHGFSASANVDFFICRRNDNECNNYAGSFSVLGGP 3196
NunuH1 HHTVAELAEEINSLRNSERVFAGFVLHGFGASATRKVFVN-------DHYAGTFNVLGDR 3242
MecrH1 GHNIEELEEVLRSLRLKSRVFAGFVLSGIRTTAVVKVYIKSG-TDSDDEYAGSFVILGGA 3255
HacrH1 GHDINELEAIINDLRNKDRIFAGFVLSGIRITATVKVFIHETGVTDHEEFAGKFAILGGE 3263
HarufH1 GHDINELEAIINELRNKDRIFAGFVLSGIRTTATVKVFIHGTGATDHEEFAGKFAILGGE 3263
HadishaH1 GHDINELEAIINELRNKDRIFAGFVLSGIRITATVKVFIHGTGATDHEEFAGKFAILGGE 3263
HadivH1 GHDINELEAIINELRNKDRIFAGFVLSGIRITATVKVFIHGTGATDHEEFAGKFAILGGE 3178
HaasH1 GHDIQEVEEILRELRNRDRVFAGFVLSGIRITAMVKVFIHSKNETRHEEYAGEFAILGGE 3263
HarubH1 GHDIHELEEVLKELRNKDRIFAGFVLSGLQISAPVKVYIHSKNETIHEEYAGEFAVLGGE 3264

CoasHaN KEMPWSNEILWKFDITNAVRQAGVST-DKNIKFHFIAETYNGTL-HHD-QVAYGIFVIRE 3331
HepoHaN KEMPWANEILWKFDITTAVHQAGVST-DKNIKFHFKIETYNGTL-FAS-ESAYAIFLVRE 3330
HeluHaD REMPWAYERLFKYDITDVAKKYDITT-DHPIKVKVTSTYYNGEP-HQEYT-DEIVAVERH 3329
ApcaH1 REMPWCFERVFKYDVAEVLQANNVDI-HKPVKIGGQLFRYDGEH-VQNFTTYYATFIERP 3333
LystH1 KEMPWAYERIMKLDVTEAIRKGKIST-DHAVKARFTSTDYQGNL-NHQ-DTDYAIIVERH 3335
PhacH1 KEMPWAYERLMKIDITETVKASSIDV-SHAVKARFTFTDYTGSK-IKE-AEDYAIFVERR 3337
PocaH1 QERRWAHERAYKLDVTEAAARLELDP-YSTFDFNVSLFDYTGQP-LPY-TLPYPLVLYRP 3322
MetuMH NEMPWAYERVYKYEITEAAKKYGLDH-DSVFNFHHKVVKYDGQE-LSA-QFKAPILVERP 3344
LisaH1 EEMPWAYERPYKLDITDAISKAGLAH-GDTVHLSTSVARYDGTP-LNV-TFPSSLIIERP 3333
CrfoH1 SEMPWAYERIYKLDMTEAASKLGLSS-ESTFHFKLTVTKYDGTA-LNV-TFPDPVIVKRA 3324
RaveH1 SEMPWAYERIYKLDMTEAAKKLGLSG-SSHFDFKLTVTKYDGSA-LDV-KFPDPVIVKRR 3339
OcvuHA -- 2896
TopaH1 -- 3314
EuscH1 -- 3342
SeofH1 -- 3342
SemaH -- 3343
LijaH2 TEMPWAFERPFKYEVTELFNKLGYKY-TDDIKVRTEVKATNGSA-L-NIEFPLPVIVYRP 3253
NunuH1 KEMPWAYERLFKYDITEVLRENNLNAGHNDITFRCEVTALDGTTALDPNTLSDPVLVLRP 3302
MecrH1 KEMPWAYERLYRFDITETVHN--LNLTDDHVKFRFDLKKYDHTE-LDASVLPAPIIVRRP 3312
HacrH1 KEMPWAYERLLKLDITDAVHR--LHLKDEEIRFRMEVTAYNGVP-VS-TKLADPLIVHRP 3319
HarufH1 KEMPWAYERLLKLDITDAVHR--LHLKDEEIRFRMEVTAYNGVP-VS-TKLADPLIVHRP 3319
HadishaH1 KEMPWAYERLLKLDITDAVHH--LHLKDEEIRFRMEVTAYNGVP-VS-TKLADPLIVHRP 3319
HadivH1 KEMPWAYERLLKLDITDAVHH--LHLKDEEIRFRMEVTAYNGVP-VS-TKLADPLIAHRP 3234
HaasH1 KEMPWAYERMLKLDITDAVNK--LHVKDEDIRFRMEVTAYNGDI-VT-TKLSQPFIVHRP 3319
HarubH1 KEMPWAYERMLKLDITDAVNK--LHLKDEDIRFRMDVTAYNGDI-VT-TQLSQPFIVHRP 3320

CoasHaN AYTDYLTLNIPVGG-NLPLPSKLVVPKKTHVRFLPVSEDY---NV-PIENLVSYTNYNKC 3386
HepoHaN AYTDYLTLNIPVGV-GYPLPSKLVVPKKTHVRFLPVSEDY---NI-PIENLVSYTNYNKC 3385
HeluHaD ADTDYDIVVIPVSK-DNTLVPKIVVKKGTRIEFVT--SDL---TD-PLEDLGSYTTMHKC 3382
ApcaH1 AGVDYDIINIPVMR-NSSINPKTVVKKGTRVRFVT--DGV---ET-PMENLGSFTNFHFC 3386
LystH1 AEQDYDVVEIPVGR-KYPLPPKIVLKKGSRVKFYPGSDTF---NS-PLENLGSYTSFSKC 3390
PhacH1 ANTDYDVVNIPIGR-EQPLPPKISLKKGTRVRFIPVSDSF---TK-PMEDLGSYTSYNRC 3392
PocaH1 ASVDFDVLVYPLYV-DKALPPKVTVRRGTKIRFHAADPSL---QGRRIRTFGSYTLFIKC 3378
MetuMH AGVDYDVAIFQLKK-DNDLAPKVVLPRGTRIRFHSAEDGV---SS-VLREMGSYTNAVYC 3399
LisaH1 ANADYDVLVVQFGA-EQAKDYKVRVKRGTRVRFHSTLEGY---VG-QVKELGTYTNSQLC 3388
CrfoH1 ANSQHDVLVLPLSV-ANQLPPKIVVRQGTQVVFHASESGV----S-SLREVGSYTNSVHC 3378
RaveH1 TNAEFDELILPIRK-ENKLPPKIVVRRGTRVLFHPTEEGI---LG-PIRELGSFTNSKLC 3394
OcvuHA -- 2896
TopaH1 -- 3314

```
EuscH1         ------------------------------------------------------------      3342
SeofH1         ------------------------------------------------------------      3342
SemaH          ------------------------------------------------------------      3343
LijaH2         PISENDVYVISIGQ-GRTPAHKIVAKRGTRFIVNPFDAESE--GKRYIMDLSSYTPFYRC      3310
NunuH1         ARVNYDVIIFQMAPTNHNPPAKVIVKKGTKLQYHPHAAIVQMGLSDAVAELGSYTSWSKC      3362
MecrH1         NNAVFDIIEIPIGK-DVNLPPKVVVKRGTKIMFMSVDEA----VTTPMLNLGSYTAMFKC      3367
HacrH1         AHASHDILVIPVGM-GHELPPKVVVKSGTKIEFTPIDSS----VDRAMVELGSFTAMAKC      3374
HarufH1        AHASHDILVIPVGK-GHQLPPKVVVKSGTKIEFTPIDSS----VDRAMVELGSFTAMAKC      3374
HadishaH1      AHASHDILVIPVGK-GHELPPKVVVKSGTKIEFTPIDSS----VDRAMVELGSFTAMAKC      3374
HadivH1        AHASHDILVIPVGK-GHELPPKVVVKSGTKIEFTPIDSS----VDRAMVELGSFTAMAKC      3289
HaasH1         AHVSHDILVIPVGA-GHDLPPKVVVKSGTKIEFTPIDSS----VNRPMVELGSFTAMAKC      3374
HarubH1        AHVSHDILVIPVGA-GHALPPKVVVKGGTKIEFTPIDSS----VDRSMVELGSFTALAKC      3375

CoasHaN        SLPPFAFDSYAVNVVHALQPGDYYFTSFS--KEHCTKGN-KIYIHVQDH--  3432
HepoHaN        SLPPFAFDSYAVNVVHALQPGDYYFTSFN--KEYCTQDK-KIYIHVQDH--  3431
HeluHaD        KIPPFSNNSYALNTVYKLSPGDYFFVPKN--VDLCNAGR-RIQITVEDD--  3428
ApcaH1         NIPPFSYHSFPFGEVHALSPGDYFFVNRD--VDLCNSGR-KLQITVADE--  3432
LystH1         SIPPFSYQSYALGVVHTLQPGDYFFVPKD--KALCEAGK-RIQITVEDE--  3436
PhacH1         AVPPFSYQSYALNTVHSLEPGEHFFVAAD--KALCAEGK-RVIIAVEDE--  3438
PocaH1         EALPGNADALSLDVTYSLNPGEYYLALDGDLPGKCLEAG-RTILVIDEE--  3426
MetuMH         SIPPGDANTYKLEVEYTLEPGDYFFVSND--EEKCKQGD-RIQISIDEE--  3445
LisaH1         VIPPGLANSYELEMEHKLEPGDYFFTDFD--RSGCLAGH-RIMISVDQDWN  3436
CrfoH1         AIPPGQANLYDLDVAYSLEAGDYYFTSSD--KTKCQQGS-RIQITVDDE--  3424
RaveH1         AIPPGHAHAYELDRLHVLEPGDYYFVSNN--VESCKAGS-RIQISVDEE--  3440
OcvuHA         ---------------------------------------------------  2896
TopaH1         ---------------------------------------------------  3314
EuscH1         ---------------------------------------------------  3342
SeofH1         ---------------------------------------------------  3342
SemaH          ---------------------------------------------------  3343
LijaH2         DVPEFGFQKYNFNQEYTLAPGTYYFTTAD--SETCKQGA-KFILEIAE---  3355
NunuH1         IVPPFGYRRYQFNTVYSLSPGNYYFSAPT--VEMCKQNR-KLILAVEEE--  3408
MecrH1         KVPPFSFHAFELGKMYSVESGDYFMTAST--TELCNDNNLRIHVHVDDE--  3414
HacrH1         IVPPFTYNAFELNKVYSVDHGDYYITAGT--HELCEK-NVRLNVHVEEE--  3420
HarufH1        IVPPFTYNAFELNKVYSVDHGDYYITAGT--HELCEQ-NVRLNVHVEEE--  3420
HadishaH1      IVPPFTYNAFELNKVYSVDHGDYYITAGT--HELCEQ-NVRLNVHVEEE--  3420
HadivH1        IVPPFTYNAFELNKVYSVDHGDYYITAGT--HELCEQ-NVRLNVHVEDE--  3335
HaasH1         IVPPFSYNGFELDKVYSVEHGDYYITAGT--TGLCEQ-NLKLNIHVEHE--  3420
HarubH1        IVPPFTYHGFELNKVYSVHHGDYYITAGT--KELCKQ-NLRLNIHVEHE--  3421
```
